# Induction of Three New Secondary Metabolites by the Co-Culture of Endophytic Fungi *Phomopsis asparagi* DHS-48 and *Phomopsis* sp. DHS-11 Isolated from the Chinese Mangrove Plant *Rhizophora mangle*

**DOI:** 10.3390/md22080332

**Published:** 2024-07-24

**Authors:** Jingwan Wu, Jingjing Ye, Juren Cen, Yuanjie Chen, Jing Xu

**Affiliations:** 1 Collaborative Innovation Center of Ecological Civilization, School of Chemistry and Chemical Engineering, Hainan University, Haikou 570228, China; 20081700110009@hainanu.edu.cn (J.W.); 2021110817000019@hainanu.edu.cn (J.Y.); 2 School of Life and Health Sciences, Hainan University, Haikou 570228, China; cenjr@hainanu.edu.cn (J.C.); 22220860000042@hainanu.edu.cn (Y.C.)

**Keywords:** mangrove endophytic fungi, *Phomopsis asparagi*, *Phomopsis* sp., co-culture

## Abstract

Co-cultivation is a powerful emerging tool for awakening biosynthetic gene clusters (BGCs) that remain transcriptionally silent under artificial culture conditions. It has recently been used increasingly extensively to study natural interactions and discover new bioactive metabolites. As a part of our project aiming at the discovery of structurally novel and biologically active natural products from mangrove endophytic fungi, an established co-culture of a strain of *Phomopsis asparagi* DHS-48 with another *Phomopsis* genus fungus DHS-11, both endophytes in mangrove *Rhizophora mangle*, proved to be very efficient to induce the production of new metabolites as well as to increase the yields of respective target metabolites. A detailed chemical investigation of the minor metabolites produced by the co-culture of these two titled fungal strains led to the isolation of six alkaloids (**1**–**6**), two sterols (**7**, **8**), and six polyketides (**9**–**14**). In addition, all the compounds except **8** and **10**, as well as three new metabolites phomopyrazine (**1**), phomosterol C (**7**), and phomopyrone E (**9**), were not present in discrete fungal cultures and only detected in the co-cultures. The structures were elucidated on the basis of spectroscopic analysis, and the absolute configurations were assumed by electronic circular dichroism (ECD) calculations. Subsequently, the cytotoxic, immunosuppressive, and acetylcholinesterase inhibitory properties of all the isolated metabolites were determined in vitro. Compound **8** exhibited moderate inhibitory activity against ConA-induced T and LPS-induced B murine splenic lymphocytes, with IC_50_ values of 35.75 ± 1.09 and 47.65 ± 1.21 µM, respectively.

## 1. Introduction

Endophytic fungi from special ecological niches such as mangrove ecosystems are one of the most pivotal and promising sources of bioactive natural products, presumably owing to their intriguing structural skeleton and the promising pharmacological effect of their secondary metabolites, making them attractive repositories for structurally unique secondary metabolites endowed with numerous biological activities [1,2,3,4,5]. Until 2020, at least 1090 new structures have been reported from mangrove fungal endophytes, including polyketides, terpenes, alkaloids, and peptides representing the main chemotypes [6,7,8,9,10]. Nevertheless, owing to the rise in whole-genome sequences, most mangrove endophytic fungi were demonstrated to possess significantly more biosynthetic gene clusters (BGCs) than the number of compounds they produce previously expected [11,12]. The co-culturing of two or more different fungi within a confined vessel in a manner that approximates what they are forced to do in nature may trigger the expression of silent biosynthetic pathways and uncover unprecedented chemical diversity. These stimulated secondary metabolites are probably being used by those fungi to help fight for their survival [13].

In our efforts to identify new bioactive secondary metabolites from mangrove-derived fungi, we previously investigated the secondary metabolites of two strains of the fungal genus *Phomopsis*, namely *P. asparagi* DHS-48 and *Phomopsis* sp. DHS-11, from which three new dimeric xanthones phomoxanthones L-N have been isolated and the yields of respective target metabolites enhanced simultaneously [14].

**Figure 1:**
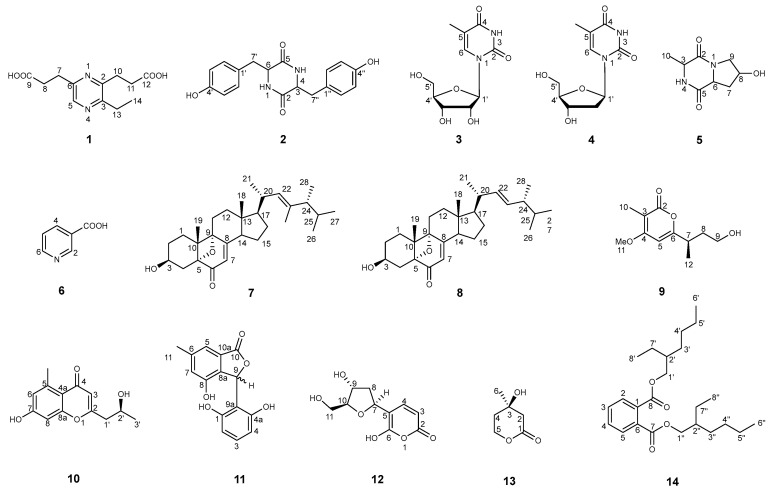
Structures of the isolated compounds **1**–**14**.

To highlight the metabolite modifications that are caused by fungal interactions, an HPLC chromatogram with UV detection (HPLC-UV) and molecular networking were adapted to the analysis of the crude EtOAc extract of a 30-day solid rice medium as previously reported [14]. Comparing the HPLC profiles (Figure S1a) of the whole co-culture to those from the monocultures demonstrated that compounds **1**, **3**–**5**, **7**, **9**, **12,** and **14** were induced metabolites that were not produced when the two fungi were cultured alone. These observations were further confirmed by the UPLC-ESI-MS/MS-based molecular networking (Figure S1b) generated through the online Global Natural Products Social Molecular Networking (GNPS) platform; nevertheless, some minor components cannot be detected by this technique. The chemical investigation of the minor metabolites obtained following the co-cultivation of these two titled fungal strains, including a new alkaloid (**1**), a new sterol (**7**), a new pyranone (**9**), as well as 11 known compounds such as *cyclo*(L-Tyr-L-Tyr) (**2**) [15], 5-methyl-uridine (**3**) [16], thymidine (**4**) [17], *cyclo*(L-Hyp-L-Ala) (**5**) [18], nicotine acid (**6**) [19], 3*β*-hydroxy-5,9-epoxy-(22*E*,24*R*)-ergosta-7,22-dien-6-dione (**8**) [20], 2-(2′-hydroxypropyl)-5-methyl-7-hydroxychromone (**10**) [21], 3-(2,6-dihydroxyphenyl)-4-hydroxy-6-methyl-isobenzofuran-1(3*H*)-one (**11**) [22], 3-(2-deoxy-*β*-*erythro*-pentofuranosyl)-6-hydroxy-2*H*-pyran-2-one (**12**) [23], (*R*)-mevalonolactone (**13**) [24], and *bis*(2-ethylhexyl) phthalate (**14**) [25], were isolated from the co-culture extracts of DHS-48 and DHS-11 (Figure 1). Herein, we report on the phylogenetic analysis of fungal strains DHS-48 and DHS-11, followed by the isolation, structure elucidation, and biological activities of these compounds.

## 2. Results and Discussion

### 2.1. Phylogenetic Analysis of Fungal Strains DHS-48 and DHS-11

**Figure 2:**
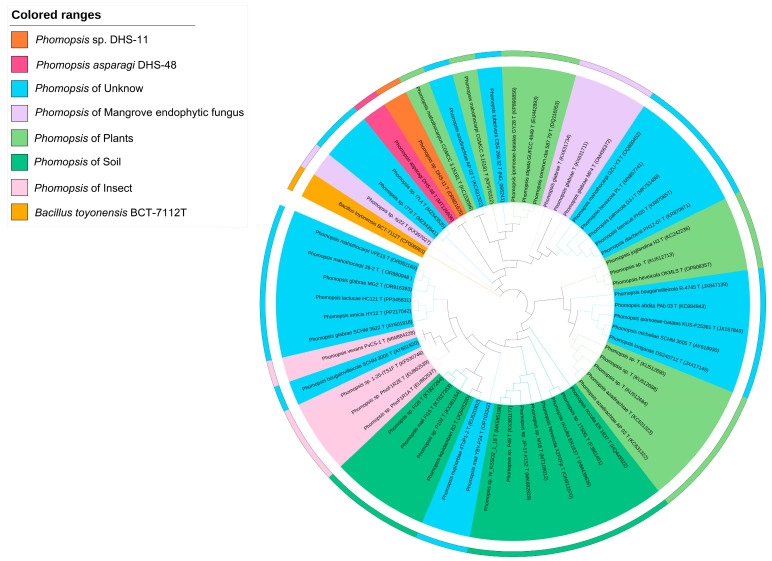
Unrooted neighbor-joining phylogenetic tree based on the ITS gene sequences showing the taxonomic positions of DHS-48, DHS-11, and type strains of closely related *Phomopsis* taxa. The values at each node represent the bootstrap values from 1000 replicates, and the scale bar represents 0.05 substitutions per nucleotide. *Bacillus toyonensis* BCT-7112T served as an outgroup.

The strains DHS-48 and DHS-11 were isolated from the Chinese mangrove plant *Rhizophora mangle* as endophytes and identified using the ITS region. BLAST search results indicated that DHS-48 and DHS-11 were found to have 100% identity to the type strain *Phomopsis asparagi* strain A0640 (KF498860) and *Phomopsis* sp. strain CBS:123 (OR801625), respectively. Based on the ITS gene sequences, a total of 60 *Phomopsis*-type strains originated from different ecosystems (including mangrove habitats, plants, soil, insects, and unknown origin) were retrieved from Genbank to construct the phylogenetic tree using unrooted neighbor-joining (NJ) algorithm. Phylogenetic and correlation analysis showed that the ITS sequence of DHS-48 clustered with other *Phomopsis* species from different ecological niches, e.g., DHS-11 from the same host, mangroves (3 strains), plants (5 strains), and unknown origin (10 strains), within a mono phylogenetic group in maximum parsimony with bootstrap support >75% (Figure 2). Amongst them, the ITS gene sequence of DHS-48 most closely resembled DHS-11 and formed a sister clade with 98% bootstrap support. Considering the impact of the taxonomic criteria and ecological impact, we selected *Phomopsis asparagi* DHS-48 and *Phomopsis* sp. DHS-11 belonging originally to the same habitat for the prioritization of co-cultivation to mimic the co-existing occurring interactions in naturally ecological situations to induce the production of new natural products derived from fungal interactions.

### 2.2. Structure Elucidation of New Compounds

**Figure 3:**
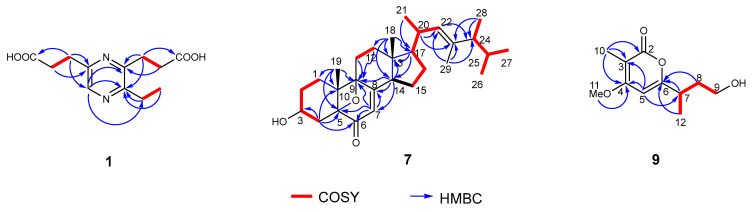
Key COSY and HMBC correlations of compounds **1**, **7**, and **9**.

Phomopyrazine (**1**) was obtained as a colorless amorphous powder. HRESIMS gave an [M + Na]^+^ ion peak at *m*/*z* 275.1002 (calcd for [M + Na]^+^ 275.1008), supporting a molecular formula of C_12_H_16_N_2_O_4_. The 1D NMR data (Table 1) of **1** in combination with distortionless enhancement by polarization transfer (DEPT) and heteronuclear single quantum coherence (HSQC) spectrum revealed the existences of a methyl [*δ*_H_ 1.28, (t, *J* = 7.5 Hz), *δ*_C_ 13.2, q, 14-CH_3_], five methylenes [*δ*_H_ 3.02 (t, *J* = 7.5 Hz), *δ*_C_ 31.7, t, CH_2_-7; *δ*_H_ 2.64 (t, *J* = 7.5 Hz), *δ*_C_ 36.2, t, CH_2_-8; *δ*_H_ 3.08 (t, *J* = 7.5 Hz), *δ*_C_ 30.4, t, CH_2_-10; *δ*_H_ 2.66 (t, *J* = 7.5 Hz), *δ*_C_ 35.3, t, CH_2_-11; *δ*_H_ 2.87 (q, *J* = 7.5 Hz), *δ*_C_ 28.2, t, CH_2_-13], an olefinic methine at *δ*_H_ (8.23, 1H, s, CH-5), and five quaternary carbons [including two carboxylic carbonyl at *δ*_C_ 179.0 (C-9) and *δ*_C_ 179.1 (C-12)]. The ^1^H-^1^H COSY correlations (Figure 3) suggested the presence of the fragments CH_2_(7)-CH_2_(8), CH_2_(10)-CH_2_(12), and CH_2_(13)-CH_3_(14) incorporating the HMBC correlations of H_2_-7/C-5, C-6, and C-9; H-5/C-3 and C-13; and H_2_-10/C-3 and C-4 and indicated the existence of the 2,6-pyrazinedipropanoic acid group. An additional ethyl moiety located at C-3 was corroborated by the HMBC correlations of H_2_-13/C-2, C-3, and H_3_-14/C-3. Thus, the structure of **1** was established and named as phomopyrazine.

**Figure 4:**
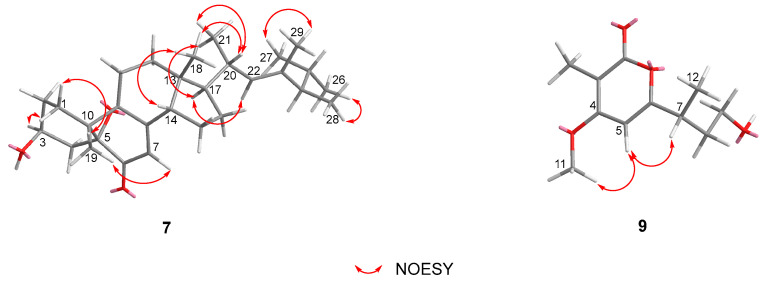
Key NOESY correlations of compounds **7** and **9**.

**Figure 5:**
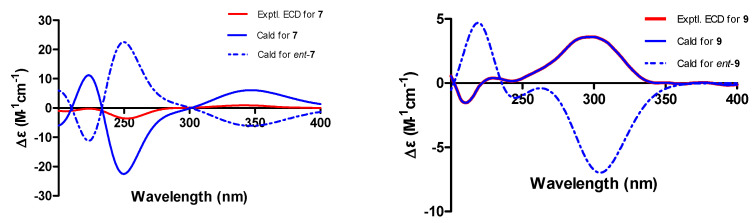
Experimental and calculated electronic circular dichroism (ECD) spectra of **7** and **9**.

Phomosterol C (**7**) was obtained as a colorless amorphous powder, and its molecular formula was established as C_29_H_44_O_3_ based on HRESIMS data (*m*/*z* 441.3363 [M + H]^+^, calcd for C_29_H_45_O_3_ 441.3362) indicating eight indices of unsaturation. The ^1^H NMR spectrum (Table 2) showed the presence of a series of characteristic signals for three methyl singlets (*δ*_H_ 0.68, s, 18-CH_3_; 0.99, s, 19-CH_3_; 1.53, s, 29-CH_3_), four methyl doublets [*δ*_H_ 0.98 (d, *J* = 6.8 Hz), 21-CH_3_; *δ*_H_ 0.80 (d, *J* = 6.6 Hz), 26-CH_3_; *δ*_H_ 0.87 (d, *J* = 6.6 Hz), 27-CH_3_; 0.98 (d, *J* = 6.8 Hz), 28-CH_3_], an oxymethine (*δ*_H_ 3.92, m, CH-3), and two olefinic proton [*δ*_H_ 5.58 (d, *J* = 2.0 Hz), CH-7; *δ*_H_ 4.94 (d, *J* = 9.6 Hz), CH-22]. The ^13^C NMR and HSQC spectral data of **7** exhibited 29 carbon signals, including seven methyls, seven methylenes, eight methines (two olefinic and one oxygenated), and seven non-hydrogenated carbons (one carbonyl, two olefinic, and two oxygenated). These observed data suggested that **7** was an ergostane-type pentacyclic steroid, and closely resembled those of the co-isolated 3*β*-hydroxy-5,9-epoxy-(22*E*,24*R*)-ergosta-7,22 dien-6-one (**8**) previously isolated from the endophytic fungus *Chaetomium* sp. M453 [20]. The main difference between the two compounds is the presence of a methyl group (*δ*_H_ 1.53, s, *δ*_C_ 13.4, q, 29-CH_3_) located at C-23 (*δ*_C_ 137.3, s) in **7** instead of olefinic methine (CH-23) in **8**. Confirming evidence was obtained from the ^1^H-^1^H COSY correlation (Figure 3) of H_3_-21/H-20/H-22 and H_3_-28/H-24/H-25/H_3_-26/H_3_-27, and HMBC correlations from H_3_-29 to C-22 (*δ*_C_ 132.5, d), C-23, and C-24 (*δ*_C_ 51.8, d) (Figure 2). The relative stereochemistry of **7** was determined by the analysis of the NOESY spectrum (Figure 4). Diagnostic correlations positioned H_b_-1, H_3_-19, H-7, H-14 and H_3_-18, and H-20 on the *α*-face and H_a_-1, H-3, H-17, and H_3_-28 on the *β*-face of **7**, whereas the configuration of the double bond at *Δ*22 was deduced to be *E* by the comparison of the chemical shifts with those of the same positions of **8** and the observed NOE correlations (Figure 4) between H_3_-29/H_3_-26 and H-22/H_3_-28/H_3_-27. The absolute configuration was assigned by the comparison of the experimental and simulated electronic circular dichroism (ECD) spectra generated by the time-dependent density functional theory (TDDFT) calculations at the B3LYP/6-31+G(d,p) level using the Gaussian 09 program. The calculated and experimental curves showed a high degree of coincidence (Figure 5). Therefore, the structure of **7** was elucidated as 3*β*-hydroxy-3*S*,5*R*,9*R*,10*R*,13*R*,14*S*,17*R*,20*R*,22*E*,24*R*-5,9-epoxy-23-methyl-ergosta-7,22 dien-6-one.

Phomopyrone E (**9**) was obtained as a colorless amorphous powder. The molecular formula of **9** was established as C_11_H_16_O_4_ based on the HR-ESI-MS peak at *m*/*z* 235.0941 [M + Na]^+^ (calcd for C_11_H_16_O_4_ Na 235.0941), requiring four degrees of unsaturation. The ^1^H and ^13^C NMR data of **9** (Table 1) and the ^1^H-^1^H COSY spectrum (Figure 3) revealed the presence of one methoxy group (*δ*_H_ 3.94, s, *δ*_C_ 57.3, q, OCH_3_-11), two methyl groups (*δ*_H_ 1.84, s, *δ*_C_ 8.4, q, CH_3_-10; *δ*_H_ 1.26 (d, *J* = 7.0 Hz), *δ*_C_ 18.8, q, 12-CH_3_), two methylene groups (*δ*_H_ 1.90, m, H-8a, 1.73, m, H-8b, *δ*_C_ 36.6, t, CH_2_-8; *δ*_H_ 3.55, m, *δ*_C_ 60.3, t, CH_2_-9, oxygenated), a methine group (*δ*_H_ 2.86, m, *δ*_C_ 36.6, d, CH-7), and an olefinic methine (*δ*_H_ 6.43 brs, *δ*_C_ 95.8, d, CH-5, aromatic). The typical ^13^C NMR data at *δ*_C_ 168.3 (C-2), 101.2 (C-3), 169.1 (C-4), 95.8 (C-5), and 169.2 (C-6) suggested **9** was an *α*-pyrone. The COSY correlations between H_3_-12/H-7, H-7/H_2_-8, and H_2_-8/H_2_-9, as well as the HMBC correlations from H-7 to C-5 and C-6 (*δ*_C_ 169.2), and from H_2_-8 and H_3_-12 to C-6 indicated the presence of a 4-hydroxybutan-2-yl group at C-6 of the pyrone core. Furthermore, key HMBC correlations from H_3_-10 to C-2, C-3, and C-4, and from H_3_-11 to C-4 allowed the connection of CH_3_-10 and OCH_3_-11 at C-3 and C-4 positions, respectively. The proposed planer structure is identical to phomopyronol [26] isolated from the medicinal plant *Erythrina crista-galli*. Interestingly, the measured optical rotation value ${\left[\alpha \right]}_{D}^{20}$ + 60 (*c* 0.0001, MeOH) of **9** reported in this work does not correspond with those reported for phomopyronol ${\left[\alpha \right]}_{D}$ − 11 (*c* 0.5, MeOH). The absolute configuration of C-7 was assigned to be *R* in **9** by the comparison of its calculated and experimental ECD spectrum (Figure 5). As a result, the structure of **9** was determined and considered to be a new *dextro* optical isomer of the known (−)-phomopyronol.

### 2.3. Bioactivities of Isolated Compounds

All of the isolated compounds (**1**–**14**) were evaluated for their cytotoxic, immunosuppressive, and acetylcholinesterase (AChE) inhibitory activities. The results indicated that compounds **7**, **8,** and **14** showed slight in vitro cytotoxicity against human liver cells HepG2 (IC_50_ values ranging from 65.97 to 73.37 μM) and cervical cancer cells Hela (IC_50_ values ranging from 72.02 to 87.30 μM) (Table 3). Compounds **1** and **8**–**10** exhibited weak to moderate immunosuppressive activity, of which **8** was most potent against the proliferation of ConA-induced (T-cell) and LPS-induced (B-Cell) murine splenic lymphocytes with the IC_50_ values of 35.75 ± 1.09 and 47.65 ± 1.21 µM, respectively (Table 4). Nonetheless, only compound **11** showed weak inhibition of AChE with an IC_50_ value of 86.11 ± 1.56 μM (Table 5).

## 3. Materials and Methods

### 3.1. General Procedures

The optical rotations were acquired using the ATR-W2 HHW5 digital Abbe refractometer (Shanghai Physico-optical Instrument Factory, Shanghai, China). The UV spectra were obtained by using a Shimadzu UV-2600 PC spectrophotometer (Shimadzu Corporation, Tokyo, Japan), while the ECD spectra were measured on a JASCO J-715 spectra polarimeter (Japan Spectroscopic, Tokyo, Japan). All the LC/MS data were collected by the LCMS-IT-TOF instrument (Shimadzu Corporation, Tokyo, Japan) with an ESI source. The ^1^H, ^13^C, and 2D NMR spectra were acquired on a Bruker AV 400 NMR spectrometer (Bruker Corporation, Fällanden, Switzerland) using TMS as an internal standard. TLC and column chromatography (CC) were executed on silica gel (200–400 mesh, Qingdao Marine Chemical Inc., Qingdao, China) or a Sephadex-LH-20 (18–110 µm, Merck, Darmstadt, Germany), respectively. Semi-preparative HPLC was obtained on an Agilent Technologies 1200LC with a C18 column (Agilent Technologies, California, United States, 10 mm × 250 mm). High-speed centrifugation was performed at a TGL-16B Anting centrifugal machine (Anting Scientific Instrument Factory, Shanghai, China). The constant temperature water bath was in HH-2 thermostat water baths (Hervey Biotechnology Corporation, Jinan, China). All the crude extracts were eluted with a flow rate of 0.8 mL·min^−1^ over a 50 min gradient (solvents: A, H_2_O; B, MeOH), as follows: 0–5 min, 25% B; 5–15 min, 25–30% B; 15–30 min, 30–55% B; 30–40 min, 55–75% B; 40–50 min, 70–90% B; and 50–60 min, 90–100% (Figure S1).

### 3.2. Fungal Material

Endophytic fungi *Phomopsis asparagi* and *Phomopsis* sp. were isolated from the fresh root of the mangrove plant *Rhizophora mangle* collected in the Dong Zhai Gang-Mangrove Garden on Hainan Island, China, in October 2015. The fungi were identified as *Phomopsis asparagi* (strain no. DHS-48) and *Phomopsis* sp. (strain no. DHS-11) by ITS gene sequence (GenBank Accession No. MT126606 and No. OR801625). Two voucher strains were deposited at one of the authors’ laboratories (J.X.).

### 3.3. Phylogenetic Analysis

The available homologs were searched in the GenBank database (http://ncbi.nlm.nih.gov, accessed on 12 February 2024) using the BLASTN algorithm (http://www.ncbi.nlm.nih.gov/BLAST, accessed on 12 February 2024). Multiple alignments were made using the CLUSTAL_X tool in MEGA version 7.0. [27]. A phylogenetic tree based on the neighbor-joining method (NJ) was used to infer the evolutionary history of the fungi under Kimura’s two-parameter model [28], and the bootstrapping was carried out using 1000 replications. Tree visualization was carried out via the Interactive Tree of Life (iTOL) web service [29].

### 3.4. Interaction between Phomopsis asparagi, Phomopsis sp., and Co-Cultivation

A morphological investigation of the co-culture interaction between *Phomopsis asparagi* and *Phomopsis* sp. was conducted on a 90 mm PDA plate using different inoculation amounts. The circular pieces of actively growing mycelium agar from each fungus (1 cm, 0.5 cm, and 0.2 cm in diameter) were placed 5 cm from each other on a new agar plate. The plates were sealed with parafilm and incubated at 28 °C for 15 days, and the diameter of the mycelium was measured every day. Growth characteristics such as overgrowth, contact inhibition, and distance inhibition of the fungal organisms were visually observed.

### 3.5. Preparation of Phomopsis asparagi, Phomopsis sp., Co-Cultivation, Large-Scale Fermentation, and Extracts

The two fungi were independently cultivated on PDA at 28 °C for 14 days. After that, the two fungi colonies were simultaneously inoculated into an autoclaved rice solid-substrate medium in Erlenmeyer flasks (130 × 1 L), each containing 100 g of rice and 100 mL of 0.3% saline water, and fermented at 28 °C for 30 days. Following the fermentation process, the co-cultured fermentation mixes were extracted three times with EtOAc, and the filtrate was then distilled under reduced pressure to obtain 30 g of crude extract.

### 3.6. Isolation of Compounds

Using stepped gradient elution with CH_2_Cl_2_-MeOH (0–100%) on silica gel column chromatography (CC), the crude extracts were separated into nine fractions (Fr. 1–Fr. 9). The fraction Fr. 3 was subjected to open silica gel CC using gradient elution with CH_2_Cl_2_-MeOH (100:0–1:1, *v*/*v*) to obtain 6 fractions (Fr. 3.1–Fr. 3.6). Fr. 4 was subjected to open silica gel CC using gradient elution with CH_2_Cl_2_-MeOH (100:0–1:2, *v*/*v*) to obtain 7 fractions (Fr. 4.1–Fr. 4.7). The subfraction Fr. 4.3 was applied to ODS CC with the gradient elution of MeOH/H_2_O mixtures (*v*/*v*, 1:4, 3:7, 2:3, 1:1, 3:2, 7:3, 4:1, 0:1) and obtained five subfractions (Fr. 4.3.1–Fr. 4.3.5). Then, Fr. 4.3.4 were purified by semi-preparative reversed-phase HPLC using the isocratic elution of MeOH-H_2_O (60:40, *v*/*v*, 2 mL/min, UV λ_max_ 210 nm) to afford compound **9** (6 mg, *t*_R_ = 45 min). Fr. 5 was subjected to open silica gel CC using gradient elution with CH_2_Cl_2_-MeOH (100:2–1:2, *v*/*v*) to obtain 5 fractions (Fr. 5.1–Fr. 5.4). Fr. 5.2 was subjected to open silica gel CC using gradient elution with CH_2_Cl_2_-MeOH (100:2–1:2, *v*/*v*) to obtain 5 fractions (Fr. 5.2.1–Fr. 5.2.5). Then, Fr. 5.2.2 were purified by semi-preparative reversed-phase HPLC using the isocratic elution of MeOH-H_2_O (60:40, *v*/*v*, 2 mL/min, UV λ_max_ 210 nm) to afford compound **10** (5 mg, *t*_R_ = 24 min). Fr. 6 was subjected to open silica gel CC using gradient elution with CH_2_Cl_2_-MeOH (100:3–1:2, *v*/*v*) to yield 5 fractions (Fr. 6.1–Fr. 6.4). Fr. 6.2 was purified by semi-preparative reversed-phase HPLC using isocratic elution MeOH-H_2_O (70:30, *v*/*v*, 2 mL/min, UV λ_max_ 210 nm) to afford compound **13** (5 mg, *t*_R_ = 38 min). Fr. 7 was subjected to open silica gel CC using gradient elution with CH_2_Cl_2_-MeOH (100:2–1:2, *v*/*v*) to obtain 5 fractions (Fr. 7.1–Fr. 7.5). Fr. 7.2 was chromatographed on a Sephadex LH-20 CC by eluting with MeOH to yield three fractions (Fr. 7.2.1–Fr. 7.2.3). Then, Fr. 7.2.2 were purified by semi-preparative reversed-phase HPLC using the isocratic elution of MeOH-H_2_O (60:40, *v*/*v*, 2 mL/min, UV λ_max_ 210 nm) to afford compound **1** (6 mg, *t*_R_ = 35 min). Fr. 8 was applied to ODS CC with the gradient elution of MeOH/H_2_O mixtures (*v*/*v*, 1:4, 3:7, 2:3, 1:1, 3:2, 7:3, 4:1, 0:1) and obtained five subfractions (Fr. 8.1–Fr. 8.5). Fr. 8.2 was chromatographed on a Sephadex LH-20 CC by eluting with MeOH to yield three fractions (Fr. 8.2.1–Fr. 8.2.3). Then, Fr. 8.2.2 were purified by semi-preparative reversed-phase HPLC using the isocratic elution of MeOH-H_2_O (70:30, *v*/*v*, 2 mL/min, UV λ_max_ 210 nm) to afford compound **11** (6 mg, *t*_R_ = 32 min) and compound **6** (6 mg, *t*_R_ = 39 min). Fr. 8.5 was purified by HPLC using the isocratic elution of (MeOH/H_2_O, 70:30, *v*/*v*; 2 mL/min, UV λ_max_ 210 nm) to yield compound **8** (6 mg, *t*_R_ = 14 min) and compound **7** (6 mg, *t*_R_ = 37 min). Fr. 9 was subjected to open silica gel CC using gradient elution with CH_2_Cl_2_-MeOH (100:4–1:2, *v*/*v*) to obtain 5 fractions (Fr. 9.1–Fr. 9.5). The subfraction Fr. 9.3 was applied to ODS CC with the gradient elution of MeOH/H_2_O mixtures (*v*/*v*, 1:4, 3:7, 2:3, 1:1, 3:2, 7:3, 4:1, 0:1) and obtained five subfractions (Fr. 9.3.1–Fr. 9.3.5). Then, Fr. 9.3.2 were purified by semi-preparative reversed-phase HPLC using the isocratic elution of MeOH-H_2_O (20:80, *v*/*v*, 2 mL/min, UV λ_max_ 210 nm) to afford compound **12** (5 mg, *t*_R_ = 62 min), compound **3** (5 mg, *t*_R_ = 37 min), compound **2** (5 mg, *t*_R_ = 25 min), and compound **4** (8 mg, *t*_R_ = 40 min).

Fr. 9.3.3 were purified by semi-preparative reversed-phase HPLC using the isocratic elution of MeOH-H_2_O (20:80, *v*/*v*, 2 mL/min, UV λ_max_ 210 nm) to afford compound **5** (5 mg, *t*_R_ = 46 min) and compound **14** (5 mg, *t*_R_ = 28 min).

Phomopyrazine (**1**): colorless amorphous residue (MeOH); [α]^20^_D_ 0 (c 0.0001, MeOH); UV (MeOH) λ_max_ 209, 278, and 304 nm (the absorptions due to aromatic rings); HRESIMS *m*/*z* 275.1002 [M + Na]^+^ (calcd for C_12_H_16_N_2_O_4_ Na 275.1002), *m*/*z* 251.1037 [M − H]^−^ (calcd for C_12_H_15_N_2_O_4_ 251.1037).

Phomosterol C (**7**): colorless amorphous residue (MeOH); [α]^20^_D_ + 10 (c 0.0001, MeOH); UV (MeOH) λ_max_ 202, 259, and 263 nm (the absorptions due to aromatic rings); HRESIMS *m*/*z* 441.3363 [M + H]^+^ (calcd for C_29_H_45_O_3_ 441.3362).

Phomopyrone E (**9**): colorless amorphous residue (MeOH); [α]^20^_D_ + 60 (c 0.0001, MeOH); UV (MeOH) λ_max_ 213, 299, 303 nm (the absorptions due to aromatic rings); HRESIMS *m*/*z* 235.0941 [M + Na]^+^ (calcd for C_11_H_16_O_4_ Na 235.0941).

### 3.7. Theory and Calculation Details

Detailed Monte Carlo conformational analyses were performed utilizing Spartan’s 14 software (v1.1.4) using the Merck molecular force field (MMFF). The conformers exceeding a Boltzmann population of 0.4% were selected for electronic circular dichroism (ECD) calculations as presented in Tables S1–S4. Subsequently, these conformers underwent initial optimization at the B3LYP/6-31G(d) level in the gas phase, complemented by the polarizable conductor calculation model based on the polarizable continuum model (PCM). The stable conformations identified at the B3LYP/6-31G(d) level were then used in magnetic shielding constants. The theoretical calculation of ECD was conducted in MeOH using the time-dependent density functional theory (TD-DFT) at the B3LYP/6-31+g (d,p) level for all the conformers of compounds **7** and **9**. The ECD spectra were generated with the aid of the SpecDis 1.6 program (University of Würzburg, Würzburg, Germany) and GraphPad Prism 5 (University of California, San Diego, CA, USA) through the conversion of dipole-length rotational strengths into band shapes modeled by Gaussian functions with a standard deviation of 0.3 eV.

### 3.8. Cytotoxicity Assay

The liver cancer cell line, HepG2, and the cervical cancer cell line, Hela, were obtained from the Type Culture Collection of the Chinese Academy of Sciences in Shanghai, China. The cells were cultivated using an RPMI-1640 culture medium. The 3-(4,5-dimethylthiazol-2-yl)-2,5-diphenyltetrazolium bromide (MTT) method was used to evaluate cytotoxicity against the HepG2 and HeLa cells, sourced from Sigma-Aldrich, St. Louis, MO, USA, and it was employed as described previously [30]. Additionally, adriamycin (from Shanghai Macklin Biochemical Co., Ltd., with a purity of 99.8%) (Shanghai, China) and 5-fluorouracil (5-FU) (from Beijing Solarbio Science and Technology Co., Ltd., with a purity of 99.8%) (Beijing, China) served as the positive controls.

### 3.9. Splenocyte Proliferation Assay

Spleen cells were collected from BALB/c mice under aseptic conditions, plated in a 96-well plate at a concentration of 1 × 10^7^ cells/mL per well, and activated by Con A (5 μg/mL) or LPS (10 μg/mL) in the presence of various concentrations of compounds or cyclosporine A (CsA) at 37 °C and 5% CO_2_ for 48 h. Then, 20 μL CCK-8 was added to each well 4 h before the end of the incubation. The absorbance at OD_450_ was measured on ThermoFisher Scientific Multiskan™ FC Microplate Photometer (ThermoScientific, Waltham, MA, USA), and the IC_50_ value was calculated from the correlation curve between the compound concentration and the OD_450_.

### 3.10. Acetylcholinesterase Inhibitory Activity Studies

In total, 20 μL (1.2 mM) of acetylthiocholine (ATCH, from Shanghai Macklin Biochemical Co., Ltd., with a purity of 99.8%) (Shanghai, China) as the enzyme reaction substrate was added to a 96-well plate, then 20 μL of the tested compounds at different concentrations (10 μM–200 μM) and 20 μL (0.025 U/mL) of acetylcholinesterase solution (from Shanghai Macklin Biochemical Co., Ltd., with a purity of 99.8%) (Shanghai, China), and finally 100 μL of PBS phosphate buffer. After 30 min of incubation at 37 °C, 20 μL of 4% sodium dodecyl sulfate (SDS, from Shanghai Macklin Biochemical Co., Ltd., with a purity of 99.8%) (Shanghai, China) was added to terminate the reaction, and finally, 20 μL of 0.6 mM DTNB colorimetric solution was added (from Shanghai Macklin Biochemical Co., Ltd., with a purity of 99.8%) (Shanghai, China), and using a microplate reader, the intensity of the developed color was measured at 450 nm.

### 3.11. Statistical Analysis

All the cell data are presented as the mean and standard deviation of the means (S.D.), and a one-way analysis of variance (ANOVA) was used to evaluate the statistical significance of the differences between the groups by GraphPad Prism 10.2.0.

## 4. Conclusions

In summary, the co-cultivation of *Phomopsis asparagi* DHS-48 and another *Phomopsis* genus fungus DHS-11 endophytes within the same mangrove host plant *Rhizophora mangle* demonstrated to be effective to stimulate biosynthetic gene clusters with the potential to produce bioactive compounds that remain dormant under the axenic monocultures, leading to the production of an array of alkaloids, sterols, and polyketides, with some having cytotoxic, immunosuppressive, and AChE inhibitory properties.

## Figures and Tables

**Table 1:** ^1^H (400 MHz) and ^13^C (100 MHz) NMR data of **1** and **9** in CD_3_OD.

Position	1			9		
	*δ*_C_ Type	*δ*_H_ Mult (*J* in Hz)	HMBC (H to C)	*δ*_C_ Type	*δ*_H_ Mult (*J* in Hz)	HMBC (H to C)
1						
2	152.6, C		C-5, 10, 11, 13	168.3, C		
3	157.0, C		C-5, 10, 13, 14	101.2, C		
4				169.1, C		
5	141.5, CH	8.23, s	C-2, 3, 7, 10, 13	95.8, CH	6.43, s	C-3, 4, 6, 7
6	154.5, C		C-5, 7, 8	169.2, C		
7	31.7, CH_2_	3.02, t, 7.5	C-5, 6, 8, 9	36.6, CH	2.86, m	C-6, 8, 9, 12
8	36.2, CH_2_	2.64, t, 7.5	C-6, 7, 9	38.2, CH_2_	1.90, m 1.73, m	C-6, 7, 9, 12
9	179.0, C		C-7, 8, 10, 11	60.3, CH_2_	3.53, m	C-7, 8
10	30.4, CH_2_	3.08, t, 7.5	C-2, 3, 11, 12	8.4, CH_3_	1.84, s	C-2, 3, 4, 5
11	35.3, CH_2_	2.66, t, 7.5	C-2, 10, 12	57.3, CH_3_	3.94, s	C-4
12	179.1, C			18.8, CH_3_	1.26, d, 7.0	C-6, 7, 8
13	28.2, CH_2_	2.87, q, 7.5	C-2, 3, 14			
14	13.2, CH_3_	1.28, t, 7.5	C-3, 13			

**Table 2:** ^1^H (400 MHz) and ^13^C (100 MHz) NMR data of **7** and **8** in CD_3_OD.

Position	7			8	
	*δ*_C_ Type	*δ*_H_ Mult (*J* in Hz)	HMBC	*δ*_C_ Type	*δ*_H_ Mult (*J* in Hz)
1	26.6, CH_2_	H_a_ 2.29, dt, 14.5, 4.5	C-2, 3, 5, 9, 10, 19	26.6, CH_2_	H_a_ 2.20, dt, 1.5, 13.4
		H_b_ 1.50, m			H_b_ 1.39, m
2	31.0, CH_2_	H_a_ 1.87, m	C-1, 3, 4, 10	31.0, CH_2_	H_a_ 1.78, m
		H_b_ 1.46, m			H_b_ 1.36, m
3	67.8, CH	3.92, m	C-1, 2, 4, 5	67.8, CH	3.83, m
4	37.2, CH_2_	H_a_ 2.02, m		37.1, CH_2_	H_a_ 1.92, m
		H_b_ 1.63, m	C-2, 3, 5, 10		H_b_ 1.52, m
5	80.2, C		C-1, 3, 4, 6, 7, 19	80.2, C	
6	200.2, C			200.1, C	
7	120.9, CH	5.58, d, 2.0	C-5, 6, 8, 9, 14	120.9, CH	5,49, s
8	165.1, C			165.0, C	
9	76.2, C			76.1, C	
10	42.8, C			42.8, C	
11	29.3, CH_2_	H_a_ 1.97, m H_b_ 1.76, m	C-8, 9, 10, 12, 13	29.1, CH_2_	H_a_ 1.70, m
					H_b_ 1.64, m
12	36.2, CH_2_	H_a_ 1.89, m H_b_ 1.72, m	C-9, 11, 13, 14, 18	36.2, CH_2_	H_a_ 1.80, m
					H_b_ 1.62, m
13	46.2, C			46.2, C	
14	52.8, CH	2.75, ddd, 11.6, 9.8, 2.6	C-7, 8, 9, 12, 13, 15, 18	52.8, CH	2.66, dd, 11.5, 7.6
15	23.4, CH_2_	H_a_ 1.60, m H_b_ 1.52, m	C-19, 25, 24	23.4, CH_2_	H_a_ 1.52, m H_b_ 1.42, m
16	28.5, CH_2_	H_a_ 1.87, m H_b_ 1.30, m	C-13, 14, 15, 17, 20	29.3, CH_2_	H_a_ 1.89, m H_b_ 1.36, m
17	58.2, CH	1.46, m	C-13, 14, 15, 16, 20, 21, 22	57.4, CH	1.34, m
18	12.7, CH_3_	0.68, s	C-12, 13, 14, 17	20.6, CH_3_	0.90, s
19	20.6, CH_3_	0.99, s	C-1, 5, 9, 10	12.6, CH_3_	0.57, s
20	35.9, CH	2.41, m	C-13, 16, 17, 21, 22, 23	41.7, CH	1.97, m
21	21.2, CH_3_	0.98, d, 6.8	C-17, 20, 22	21.6, CH_3_	0.96, d, 6.6
22	132.5, CH	4.94, d, 9.6	C-17, 20, 21, 23, 24, 29	133.6, CH	5.13, dd, 15.2, 8.2
23	137.3, C		C-20, 22, 24, 25, 28	136.7, CH	5.19, dd, 15.2, 7.6
24	51.8, CH	1.68, m	C-22, 23, 25, 26, 27, 28, 29	44.4, CH	1.76, m
25	32.0, CH	1.55, m	C-23, 24, 26, 27, 28	34.4, CH	1.39, m
26	22.2, CH_3_	0.8, d, 6.6	C-24, 25, 27	20.5, CH_3_	0.77, d, 6.8
27	20.6, CH_3_	0.87, d, 6.6	C-24, 25, 26	20.1, CH_3_	0.75, d, 6.8
28	17.5, CH_3_	0.97, d, 6.8	C-23, 24, 25	18.2, CH_3_	0.85, d, 6.8
29	13.4, CH_3_	1.53, s	C-22, 23, 24		

**Table 3:** Cytotoxicity of compounds **1**–**14**.

Compound	IC_50_ (µM) ^a^	
	HepG2	Hela
**7**	65.97 ± 2.56	72.34 ± 2.03
**8**	77.41 ± 4.12	72.02 ± 2.89
**14**	73.37 ± 2.25	87.30 ± 0.74
**1–6**, **9–13**	-	-
Adriamycin ^b^	\	0.88 ± 0.71
Fluorouracil ^c^	179.03 ± 25.82	\

^a^ data are presented as mean ± SD from three separate experiments. ^b^ Hela cell positive control. ^c^ HepG2 cell positive control. ‘-’ stands for no inhibitory at 10 µg/mL. ‘\’stands for not tested.

**Table 4:** Immunosuppressive activity of compounds **1**–**14**.

Compound	IC_50_ (µM) ^a^	
	ConA-Induced T-Cell Proliferation	LPS-Induced B-Cell Proliferation
**1**	125.1 ± 1.12	133.87 ± 3.43
**2–7**	-	-
**8**	35.75 ± 1.09	47.65 ± 1.21
**9**	108.21 ± 1.32	112.76 ± 2.11
**10**	111.01 ± 1.02	123.84 ± 1.25
**11–14**	-	-
cyclosporin A ^b^	4.39 ± 0.02	25.11 ± 0.43

^a^ data are presented as mean ± SD from three separate experiments. ^b^ positive control. ‘-’ stands for no inhibitory effect at 200 µM.

**Table 5:** Acetylcholinesterase inhibitory activity of compounds **1**–**14**.

Compound	IC_50_ (µM) ^a^
**11**	86.11 ± 1.56
**1–10**, **12–14**	-
Donepezil ^b^	0.25 ± 0.76

^a^ data are presented as mean ± SD from three separate experiments. ^b^ positive control. ‘-’ stands for no inhibitory effect at 200 µM.

## Data Availability

The original data presented in the study are included in the article/Supplementary Materials; further inquiries can be directed to the corresponding author.

## Supplementary Materials

The following supporting information can be downloaded at: https://www.mdpi.com/article/10.3390/md22080332/s1, Figure S1: Chemical profiles of EtOAc extracts deriving from the whole co-culture of DHS-48 and DHS-11 and the monocultures of DHS-48 and DHS-11. Figure S2: ^1^H-NMR of (**1**). Figure S3: ^13^C-NMR of (**1**). Figure S4: DEPT of (**1**). Figure S5: ^1^H-^1^H COSY of (**1**). Figure S6: HSQC of (**1**). Figure S7: HMBC of (**1**). Figure S8: NOSEY of (**1**). Figure S9: HR-ESI-MS of (**1**). Figure S10: ^1^H-NMR of (**2**). Figure S11: ^13^C-NMR of (**2**). Figure S12: HR-ESI-MS of (**2**). Figure S13: ^1^H-NMR of (**3**). Figure S14: ^13^C-NMR of (**3**). Figure S15: HR-ESI-MS of (**3**). Figure S16: ^1^H-NMR of (**4**). Figure S17: ^13^C-NMR of (**4**). Figure S18: HR-ESI-MS of (**4**). Figure S19: ^1^H-NMR of (**5**). Figure S20: ^13^C-NMR of (**5**). Figure S21: HR-ESI-MS of (**5**). Figure S22: ^1^H-NMR of (**6**). Figure S23: ^13^C-NMR of (**6**). Figure S24: HR-ESI-MS of (**6**). Figure S25: ^1^H-NMR of (**7**). Figure S26: ^13^C-NMR of (**7**). Figure S27: DEPT of (**7**). Figure S28: ^1^H-^1^H COSY of (**7**). Figure S29: HSQC of (**7**). Figure S30: HMBC of (**7**). Figure S31: NOSEY of (**7**). Figure S32: HR-ESI-MS of (**7**). Figure S33: ^1^H-NMR of (**8**). Figure S34: ^13^C-NMR of (**8**). Figure S35: HR-ESI-MS of (**8**). Figure S36: ^1^H-NMR of (**9**). Figure S37: ^13^C-NMR of (**9**). Figure S38: DEPT of (**9**). Figure S39: ^1^H-^1^H COSY of (**9**). Figure S40: HSQC of (**9**). Figure S41: HMBC of (**9**). Figure S42: NOSEY of (**9**). Figure S43: HR-ESI-MS of (**9**). Figure S44: ^1^H-NMR of (**10**). Figure S45: ^13^C-NMR of (**10**). Figure S46: HR-ESI-MS of (**10**). Figure S47: ^1^H-NMR of (**11**). Figure S48: ^13^C-NMR of (**11**). Figure S49: HR-ESI-MS of (**11**). Figure S50: ^1^H-NMR of (**12**). Figure S51: ^13^C-NMR of (**12**). Figure S52: HR-ESI-MS of (**12**). Figure S53: ^1^H-NMR of (**13**). Figure S54: ^13^C-NMR of (**13**). Figure S55: HR-ESI-MS of (**13**). Figure S56: ^1^H-NMR of (**14**). Figure S57: ^13^C-NMR of (**14**). Figure S58: HR-ESI-MS of (**14**). Table S1: Gibbs free energies^a^ and equilibrium populations^b^ of low-energy conformers of phomosterol C (**7**). Table S2: Cartesian coordinates for the low-energy reoptimized MMFF conformers of phomosterol C (**7**) at B3LYP/6-31G(d,p) level of theory in gas. Table S3: Gibbs free energies^a^ and equilibrium populations^b^ of low-energy conformers of phomopyrone E (**9**). Table S4: Cartesian coordinates for the low-energy reoptimized MMFF conformers of phomopyrone E (**9**) at B3LYP/6-31G(d,p) level of theory in gas.
