# Supplementary material for: Induction of Three New Secondary Metabolites by the Co-Culture of Endophytic Fungi Phomopsis asparagi DHS-48 and Phomopsis sp. DHS-11 Isolated from the Chinese Mangrove Plant Rhizophora mangle
Source: Mar Drugs. 2024 Jul 24;22(8):332. doi: 10.3390/md22080332 (PMC11355877; doi:10.3390/md22080332)
Supplement: Supplementary file 1 [file marinedrugs-22-00332-s001.zip › marinedrugs-3081146-supplementary.pdf]

Electronic Supplementary Information

Induction of Three New Secondary Metabolites by Co-culture of Endophytic Fungi *Phomopsis asparagi* DHS-48 and *Phomopsis* sp. DHS-11 Isolated from the Chinese Mangrove Plant *Rhizophora mangle*

Jingwan Wu[1], Jingjing Ye[1], Juren Cen[2], Dandan Chen[1], Qing Li[2], Yuanjie Chen[2] and Jing Xu [1,2,*]

[1] Collaborative Innovation Center of Ecological Civilization, School of Chemistry and Chemical Engineering, Hainan University, Haikou 570228, China

[2] School of Life and Health Sciences, Hainan University, Haikou 570228, China

[*] To whom correspondence should be addressed.

Prof. Dr. Jing Xu, happyjing3@163.com; Tel.: +86-898-6627-9226; Fax: +86-898-6627-9010

Contents

Figure S1. Chemical profiles of EtOAc extracts of deriving from the whole co-culture of DHS-48 and DHS-11 and the monocultures of DHS-48 and DHS-11. -- 4

Figure S2. ^{1}H-NMR of (**1**) (400 MHz, $CDOD_3$) -- 5

Figure S3. ^{13}C-NMR of (**1**) (400 MHz, $CDOD_3$)-- 5

Figure S4. DEPT of (**1**) (400 MHz, $CDOD_3$) -- 6

Figure S5. ^{1}H-^{1}H COSY of (**1**) (400 MHz, $CDOD_3$) --- 6

Figure S6. HSQC of (**1**) (400 MHz, $CDOD_3$) --- 7

Figure S7. HMBC of (**1**) (400 MHz, $CDOD_3$) -- 7

Figure S8. NOSEY of (**1**) (400 MHz, $CDOD_3$)-- 8

Figure S9. HR-ESI-MS of (**1**) (400 MHz, $CDOD_3$) --- 8

Figure S10. ^{1}H-NMR of (**2**) (400 MHz, DMSO)-- 9

Figure S11. ^{13}C-NMR of (**2**) (400 MHz, DMSO) -- 9

Figure S12. HR-ESI-MS of (**2**) (400 MHz, DMSO) ---10

Figure S13. ^{1}H-NMR of (**3**) (400 MHz, DMSO)---10

Figure S14. ^{13}C-NMR of (**3**) (400 MHz, DMSO) ---11

Figure S15. HR-ESI-MS of (**3**) (400 MHz, DMSO) ---11

Figure S16. ^{1}H-NMR of (**4**) (400 MHz, DMSO)---12

Figure S17. ^{13}C-NMR of (**4**) (400 MHz, DMSO) ---12

Figure S18. HR-ESI-MS of (**4**) (400 MHz, DMSO) ---13

Figure S19. ^{1}H-NMR of (**5**) (400 MHz, DMSO)---13

Figure S20. ^{13}C-NMR of (**5**) (400 MHz, DMSO) ---14

Figure S21. HR-ESI-MS of (**5**) (400 MHz, DMSO) ---14

Figure S22. ^{1}H-NMR of (**6**) (400 MHz, $CDOD_3$)---15

Figure S23. ^{13}C-NMR of (**6**) (400 MHz, $CDOD_3$) ---15

Figure S24. HR-ESI-MS of (**6**) (400 MHz, $CDOD_3$) ---16

Figure S25. ^{1}H-NMR of (**7**) (400 MHz, $CDOD_3$)---16

Figure S26. ^{13}C-NMR of (**7**) (400 MHz, $CDOD_3$) ---17

Figure S27. DEPT of (**7**) (400 MHz, $CDOD_3$) ---17

Figure S28. ^{1}H-^{1}H COSY of (**7**) (400 MHz, $CDOD_3$)---18

Figure S29. HSQC of (**7**) (400 MHz, $CDOD_3$)--18

Figure S30. HMBC of (**7**) (400 MHz, $CDOD_3$) ---19

Figure S31. NOSEY of (**7**) (400 MHz, $CDOD_3$) --19

Figure S32. HR-ESI-MS of (**7**) (400 MHz, $CDOD_3$) ---20

Figure S33. ^{1}H-NMR of (**8**) (400 MHz, $CDOD_3$)---20

Figure S34. ^{13}C-NMR of (**8**) (400 MHz, $CDOD_3$) ---21

Figure S35. HR-ESI-MS of (**8**) (400 MHz, $CDOD_3$) --21
Figure S36. ^{1}H-NMR of (**9**) (400 MHz, $CDOD_3$)--22
Figure S37. ^{13}C-NMR of (**9**) (400 MHz, $CDOD_3$) --22
Figure S38. DEPT of (**9**) (400 MHz, $CDOD_3$) ---23
Figure S39. ^{1}H-^{1}H COSY of (**9**) (400 MHz, $CDOD_3$)--23
Figure S40. HSQC of (**9**) (400 MHz, $CDOD_3$)--24
Figure S41. HMBC of (**9**) (400 MHz, $CDOD_3$) ---24
Figure S42. NOSEY of (**9**) (400 MHz, $CDOD_3$) --25
Figure S43. HR-ESI-MS of (**9**) (400 MHz, $CDOD_3$) ---25
Figure S44. ^{1}H-NMR of (**10**) (400 MHz, $CDOD_3$) ---26
Figure S45. ^{13}C-NMR of (**10**) (400 MHz, $CDOD_3$)---26
Figure S46. HR-ESI-MS of (**10**) (400 MHz, $CDOD_3$)--27
Figure S47. ^{1}H-NMR of (**11**) (400 MHz, $CDOD_3$) ---27
Figure S48. ^{13}C-NMR of (**11**) (400 MHz, $CDOD_3$)---28
Figure S49. HR-ESI-MS of (**11**) (400 MHz, $CDOD_3$)--28
Figure S50. ^{1}H-NMR of (**12**) (400 MHz, DMSO) --29
Figure S51. ^{13}C-NMR of (**12**) (400 MHz, DMSO) ---29
Figure S52. HR-ESI-MS of (**12**) (400 MHz, DMSO) --30
Figure S53. ^{1}H-NMR of (**13**) (400 MHz, $CDCl_3$)--30
Figure S54. ^{13}C-NMR of (**13**) (400 MHz, $CDCl_3$) ---31
Figure S55. HR-ESI-MS of (**13**) (400 MHz, $CDCl_3$) ---31
Figure S56. ^{1}H-NMR of (**14**) (400 MHz, DMSO) --32
Figure S57. ^{13}C-NMR of (**14**) (400 MHz, DMSO) ---32
Figure S58. HR-ESI-MS of (**14**) (400 MHz, DMSO) --33
Table S1. Gibbs free energies[a] and equilibrium populations[b] of low-energy conformers of phomosterol C (**7**)--- 33
Table S2. Cartesian coordinates for the low-energy reoptimized MMFF conformers of phomosterol C (**7**) at B3LYP/6-31G(d,p) level of theory in gas--33
Table S3. Gibbs free energies[a] and equilibrium populations[b] of low-energy conformers of phomopyrone E (**9**)-- 39
Table S4. Cartesian coordinates for the low-energy reoptimized MMFF conformers of phomopyrone E (**9**) at B3LYP/6-31G(d,p) level of theory in gas---------------------------------------39

(a)

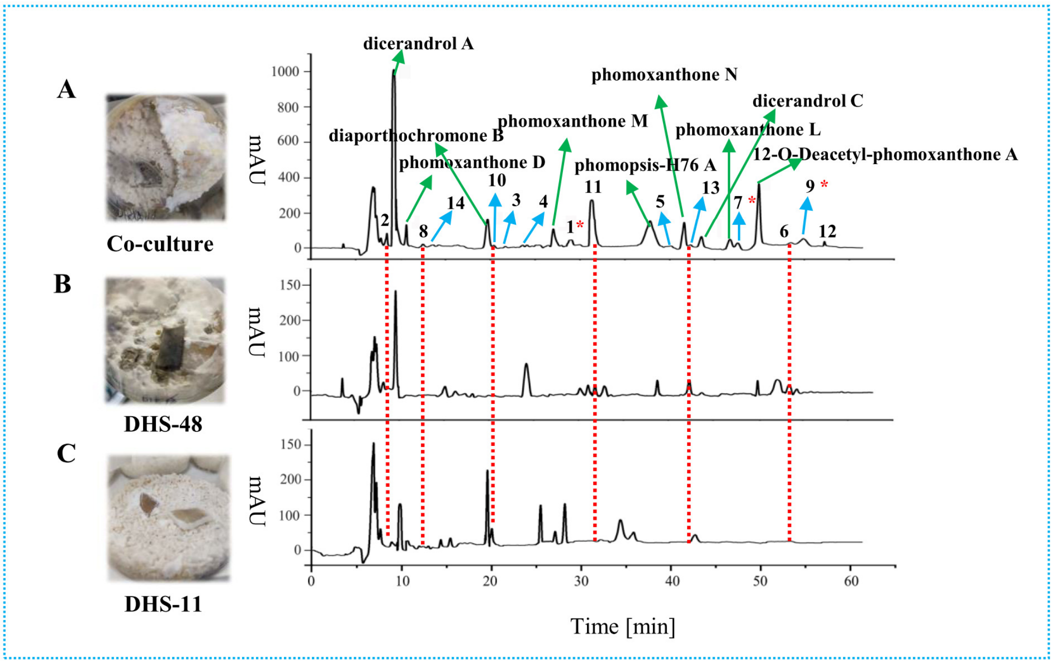

(b)

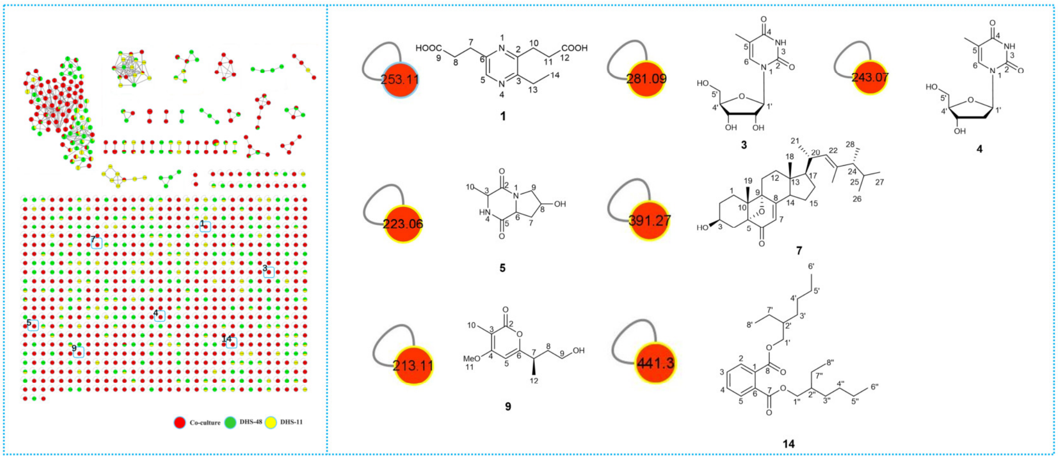

Figure S1. Chemical profiles of EtOAc extracts of deriving from the whole co-culture of DHS-48 and DHS-11 and the monocultures of DHS-48 and DHS-11. **a**. HPLC chromatograms of the EtOAc extracts deriving from (**A**) the whole co-culture of DHS-48 and DHS-11 and the monocultures of (**B**) DHS-48 and (**C**) DHS-11. **b**. Molecular network (MN) of DHS-48 (green), DHS-11 (yellow) and their co-culture (red).

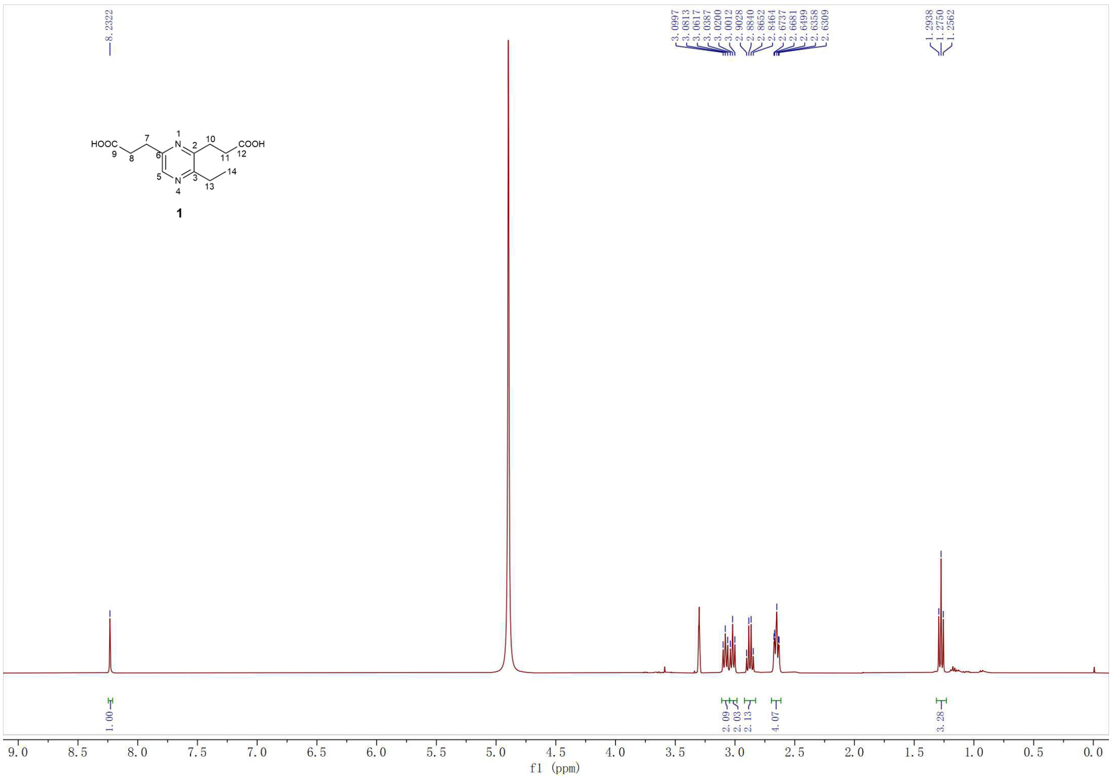

Figure S1. ^{1}H-NMR of (**1**) (400 MHz, $CDOD_3$)

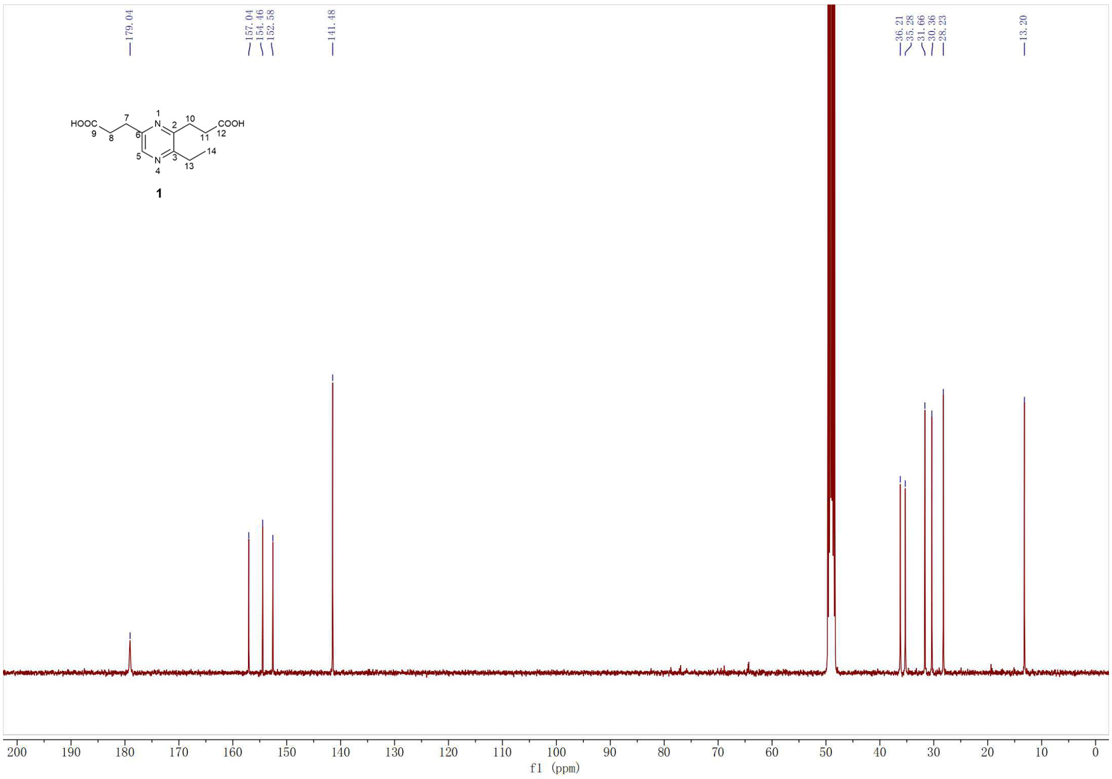

Figure S2. ^{13}C-NMR of (**1**) (400 MHz, $CDOD_3$)

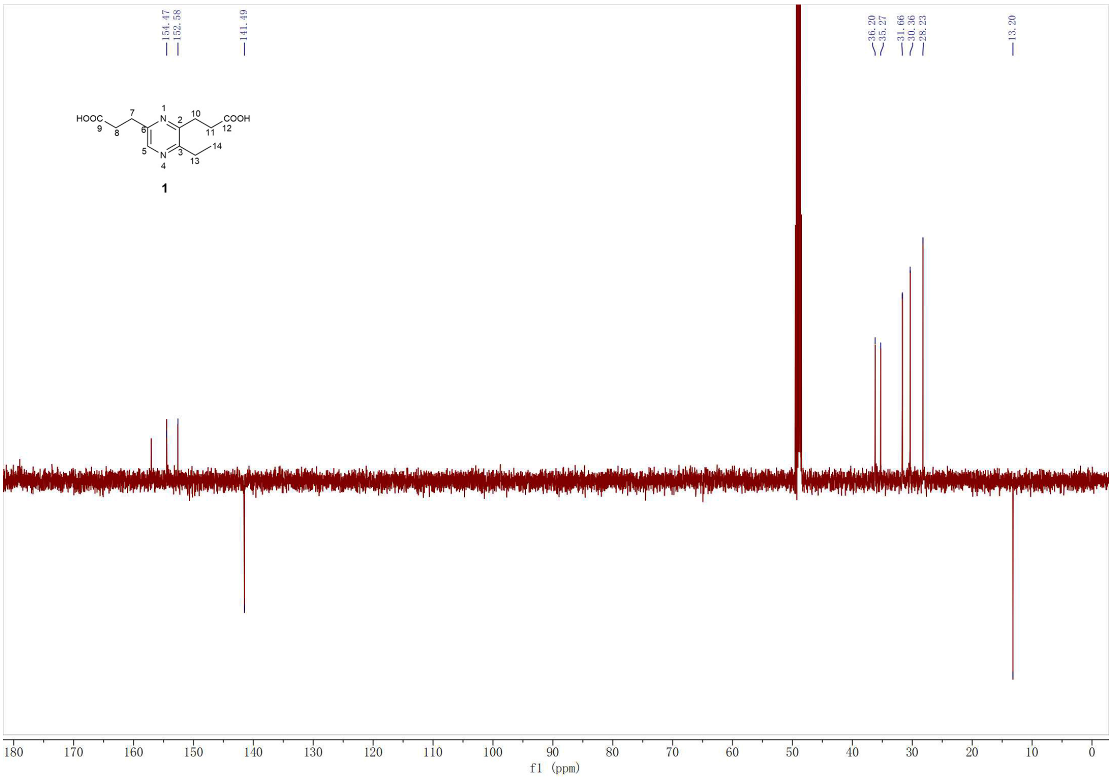

Figure S3. DEPT of (**1**) (400 MHz, $CDOD_3$)

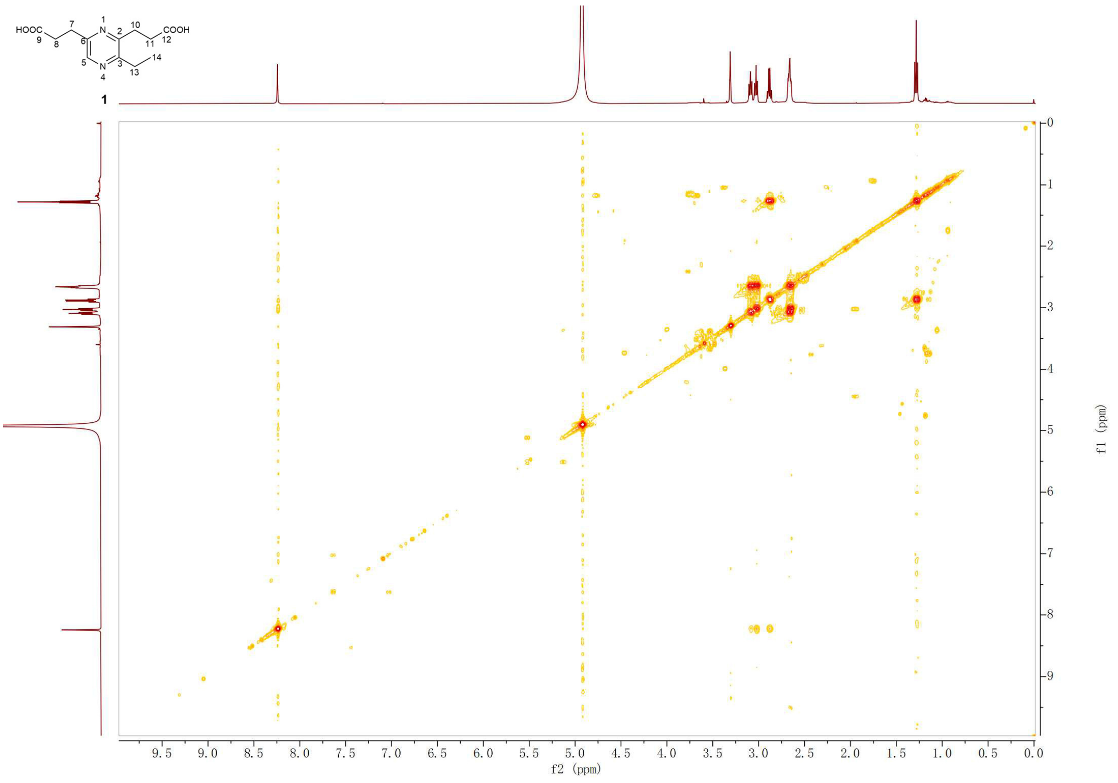

Figure S4. ^{1}H-^{1}H COSY of (**1**) (400 MHz, $CDOD_3$)

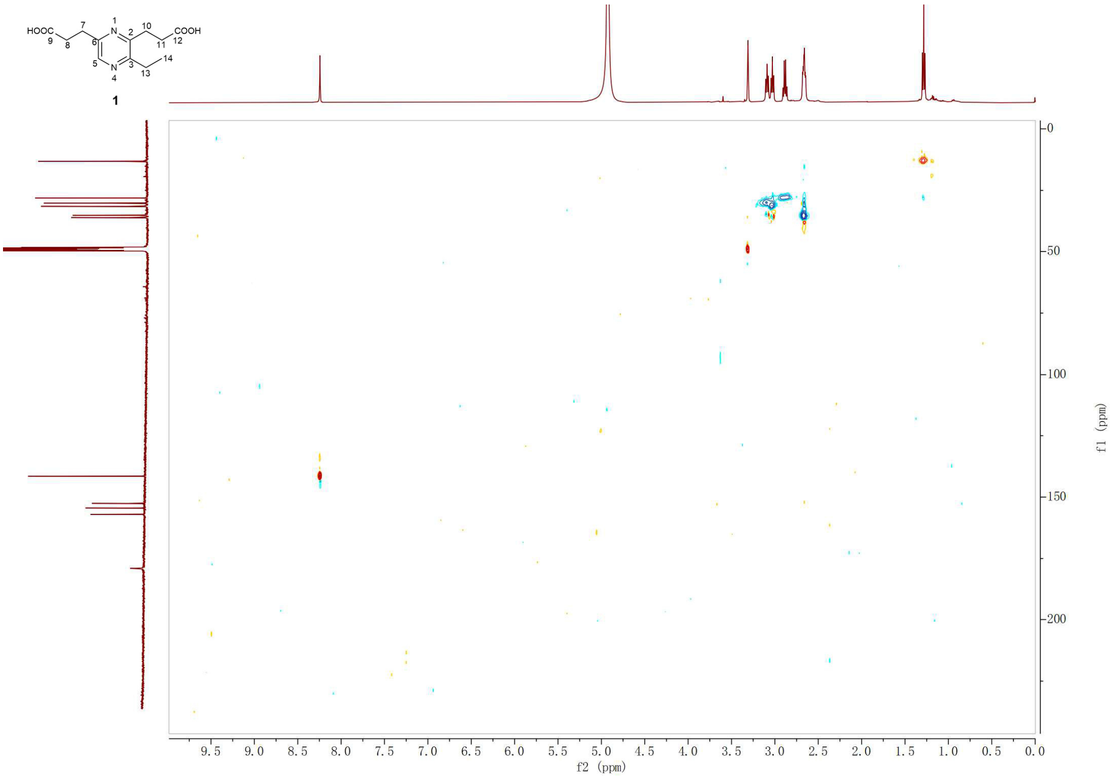

Figure S5. HSQC of (**1**) (400 MHz, $CDOD_3$)

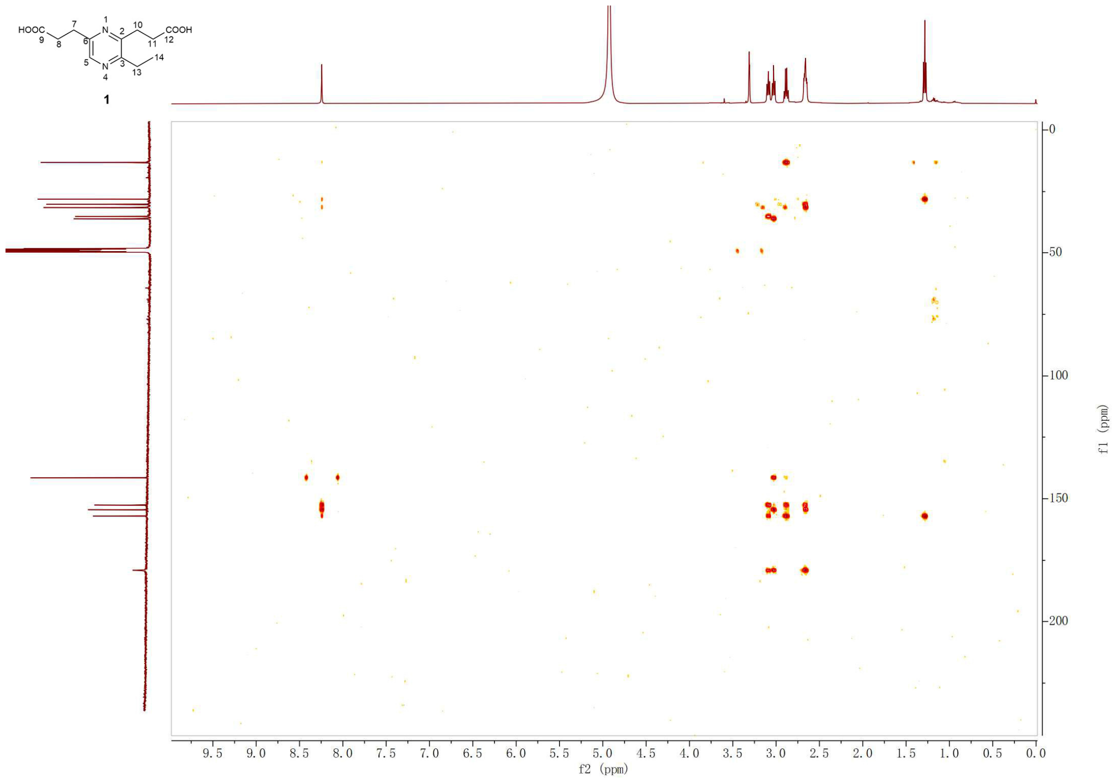

Figure S6. HMBC of (**1**) (400 MHz, CDOD3)

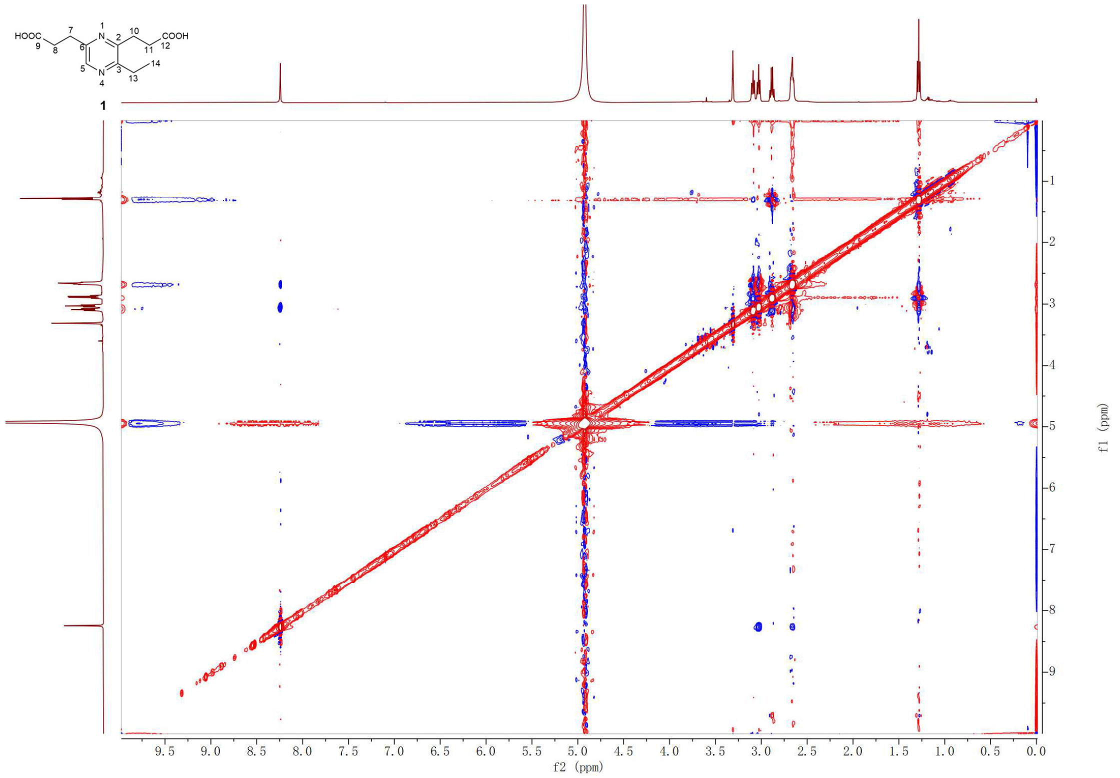

Figure S7. NOSEY of (**1**) (400 MHz, CDOD3)

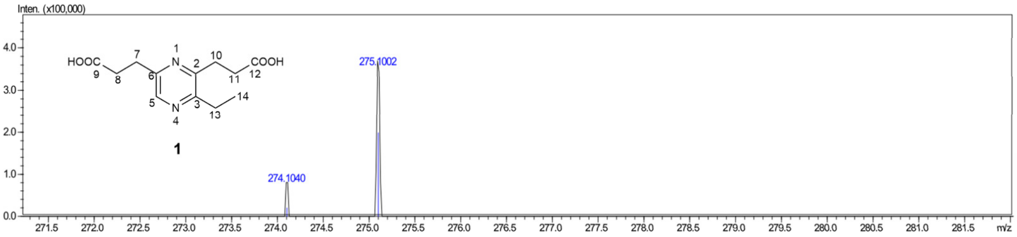

Figure S8. HR-ESI-MS of (**1**)

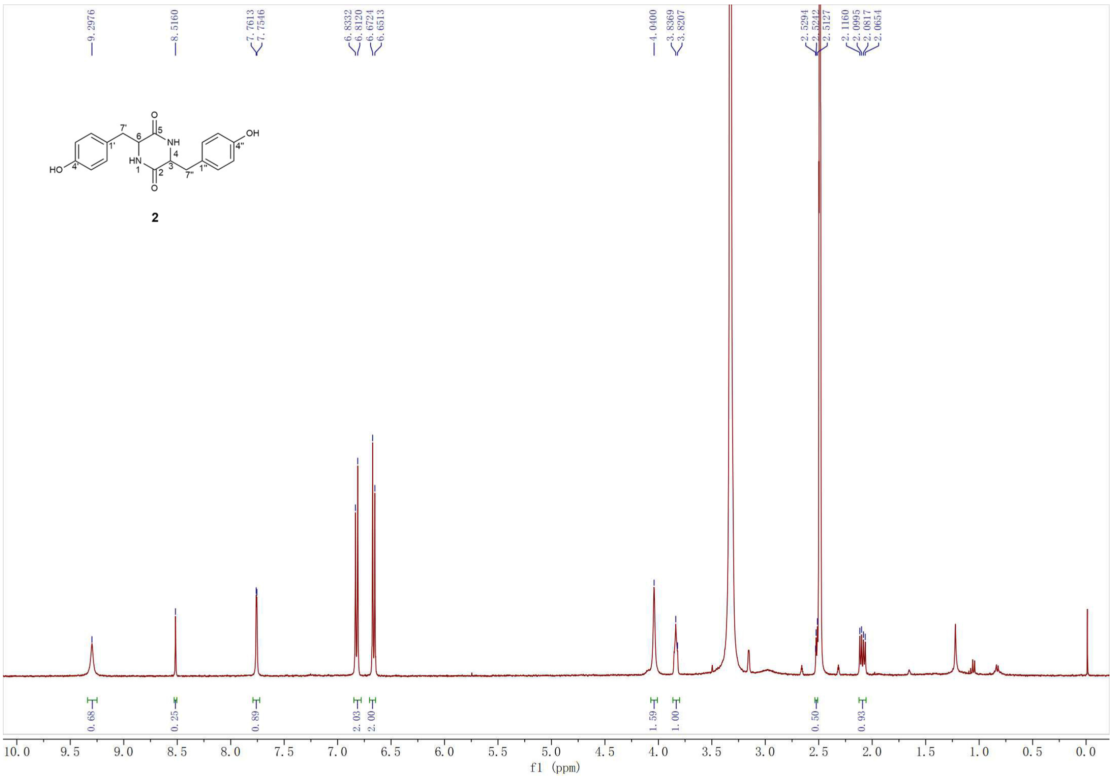

Figure S9. ¹H-NMR of (**2**) (400 MHz,DMSO)

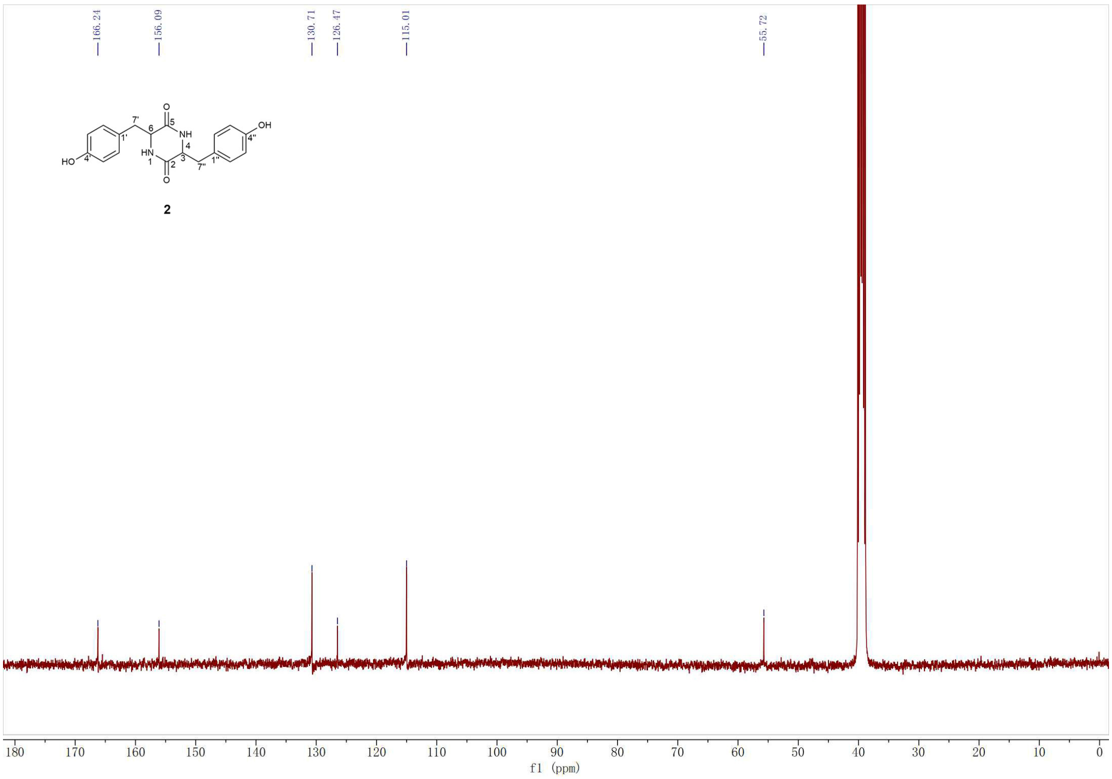

Figure S10. ^{13}C-NMR of (**2**) (400 MHz,DMSO)

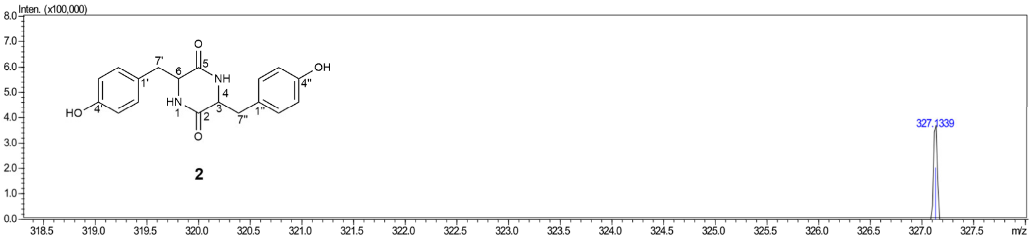

Figure S11. HR-ESI-MS of (**2**)

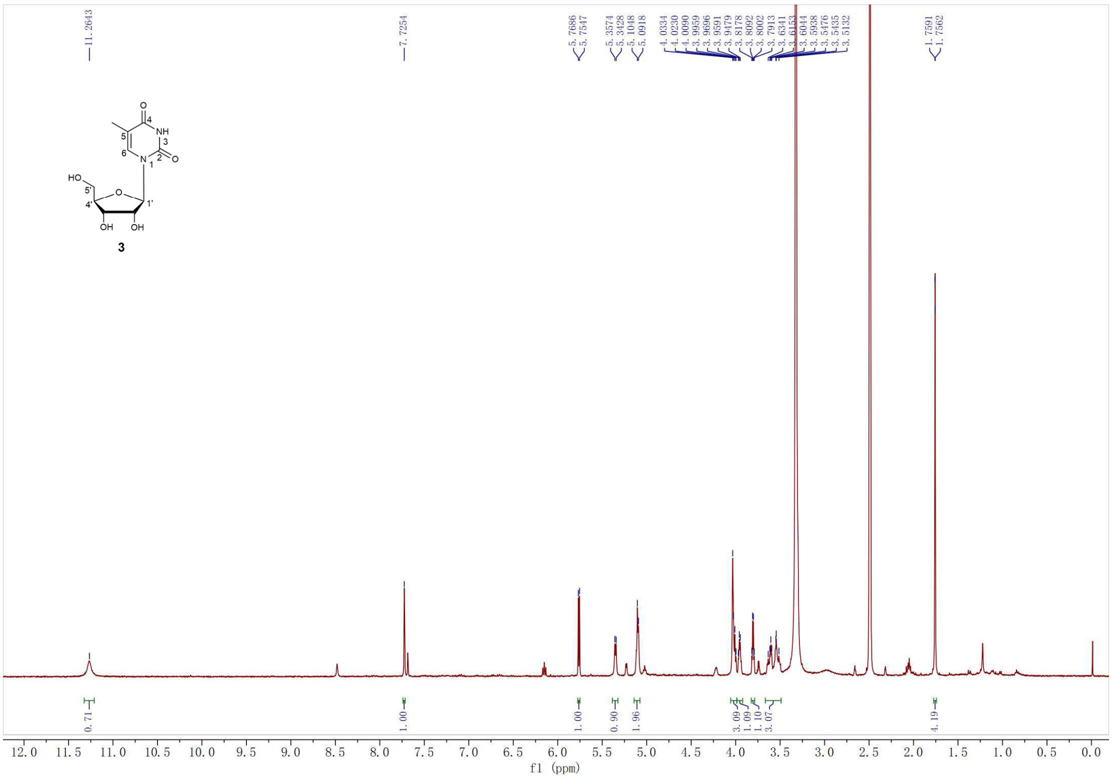

Figure S12. ^{1}H-NMR of (**3**) (400 MHz,DMSO)

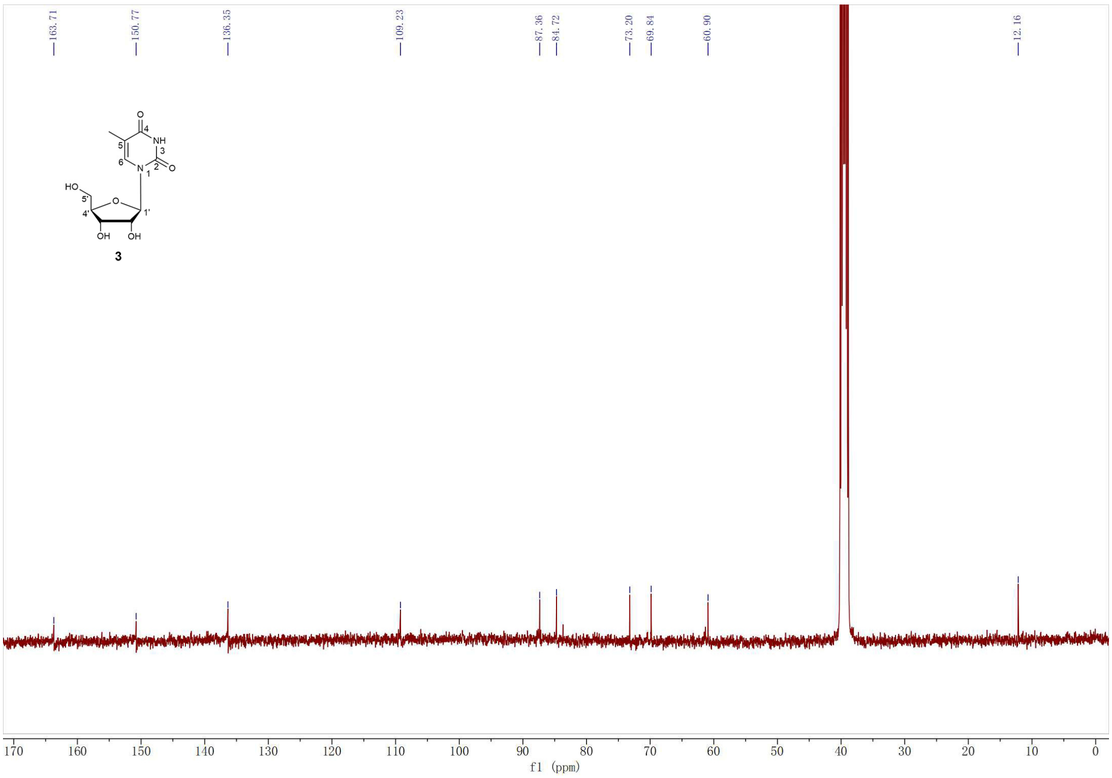

Figure S13. ^{13}C-NMR of (**3**) (400 MHz,DMSO)

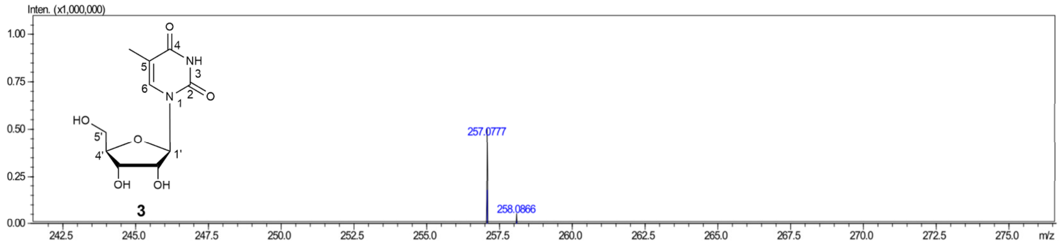

Figure S14. HR-ESI-MS of (**3**)

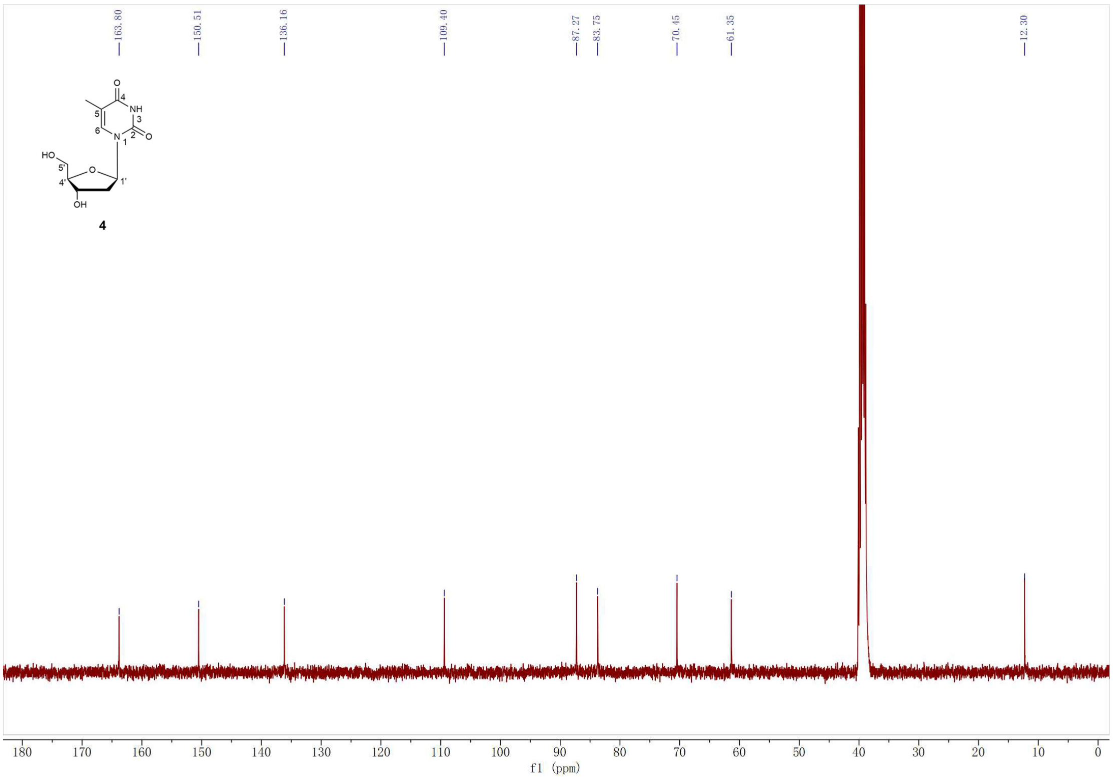

Figure S15. ^{1}H-NMR of (**4**) (400 MHz,DMSO)

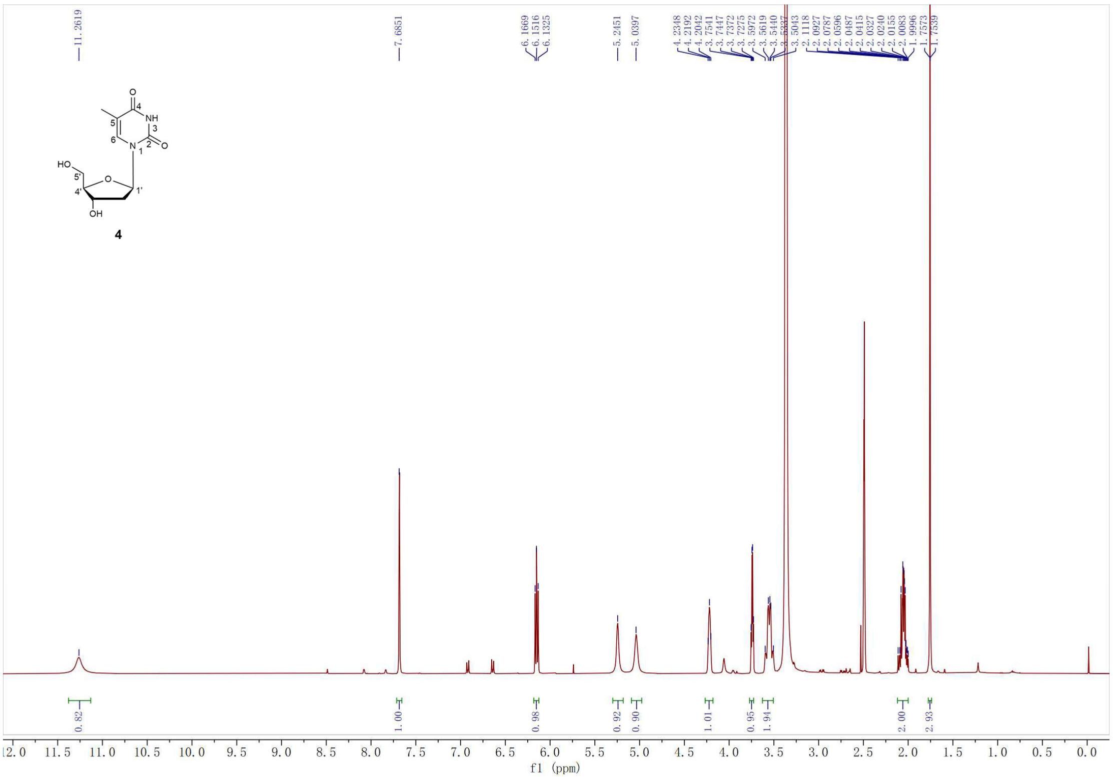

Figure S16. ^{13}C-NMR of (**4**) (400 MHz,DMSO)

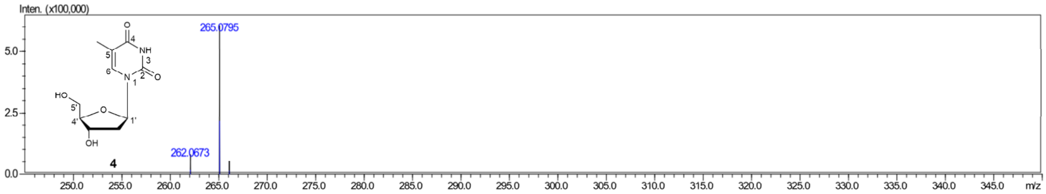

Figure S17. HR-ESI-MS of (**4**)

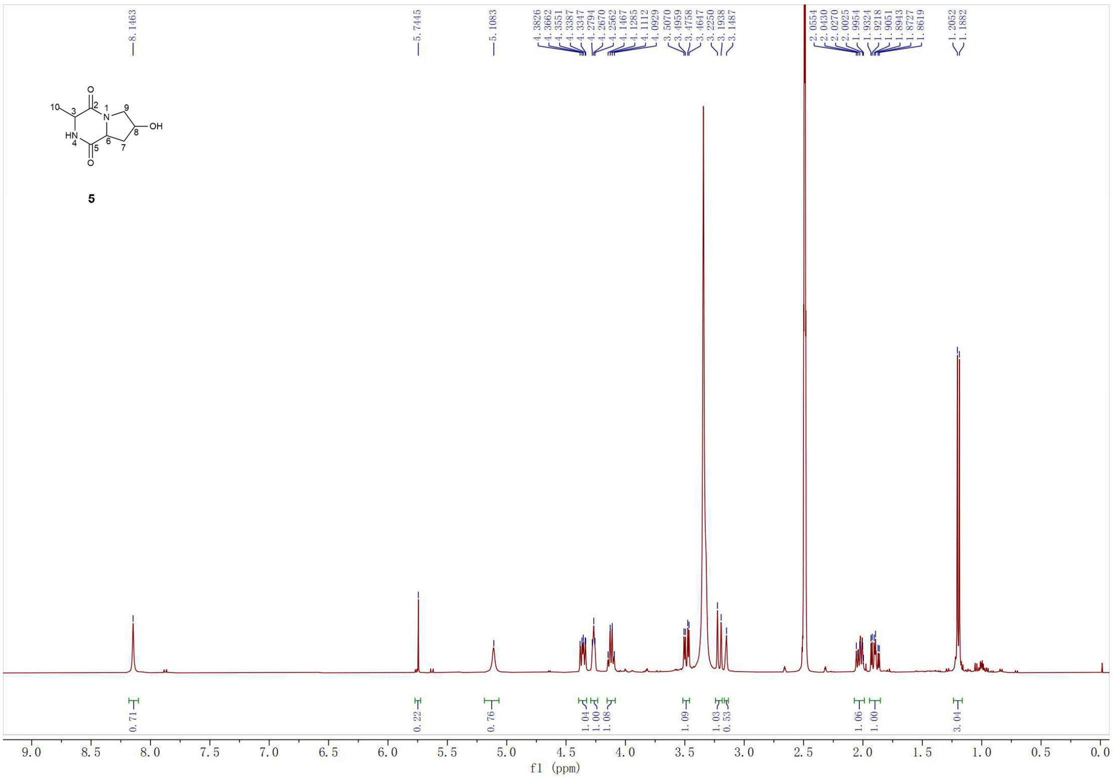

Figure S18. ^{1}H-NMR of (**5**) (400 MHz,DMSO)

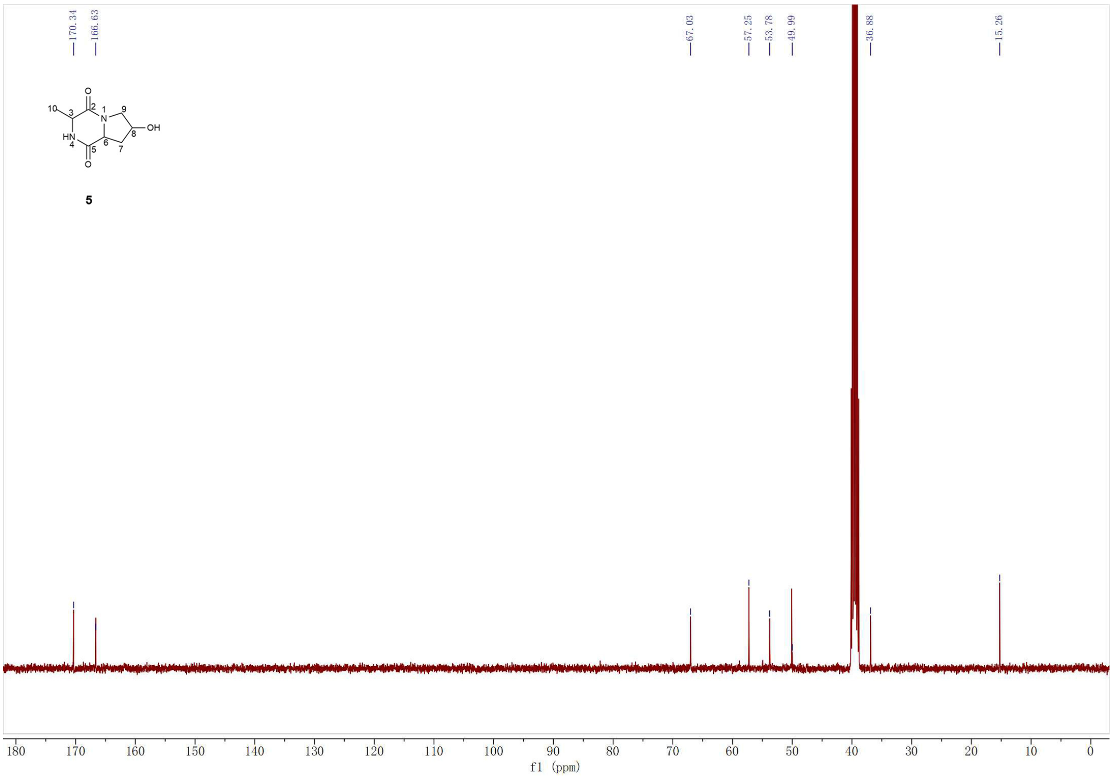

Figure S19. ^{13}C-NMR of (**5**) (400 MHz,DMSO)

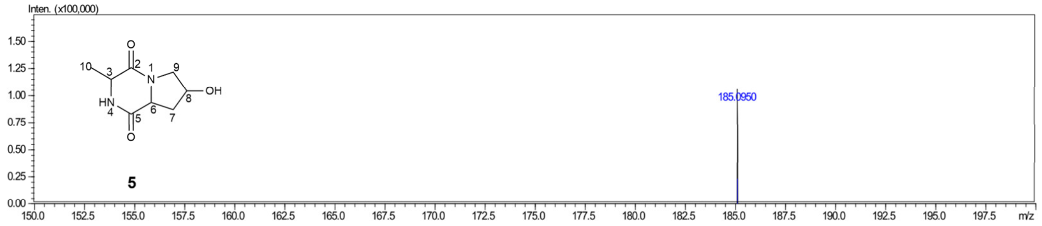

Figure S20. HR-ESI-MS of (**5**)

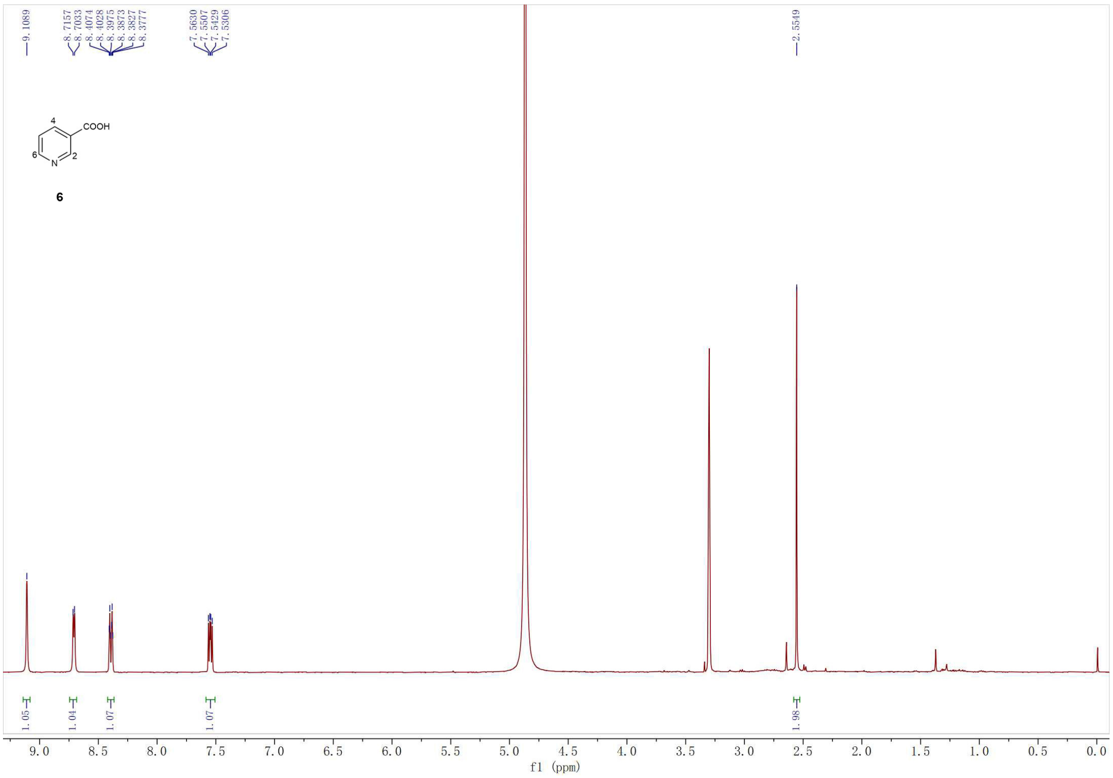

Figure S21. ¹H-NMR of (**6**) (400 MHz, $CDOD_3$)

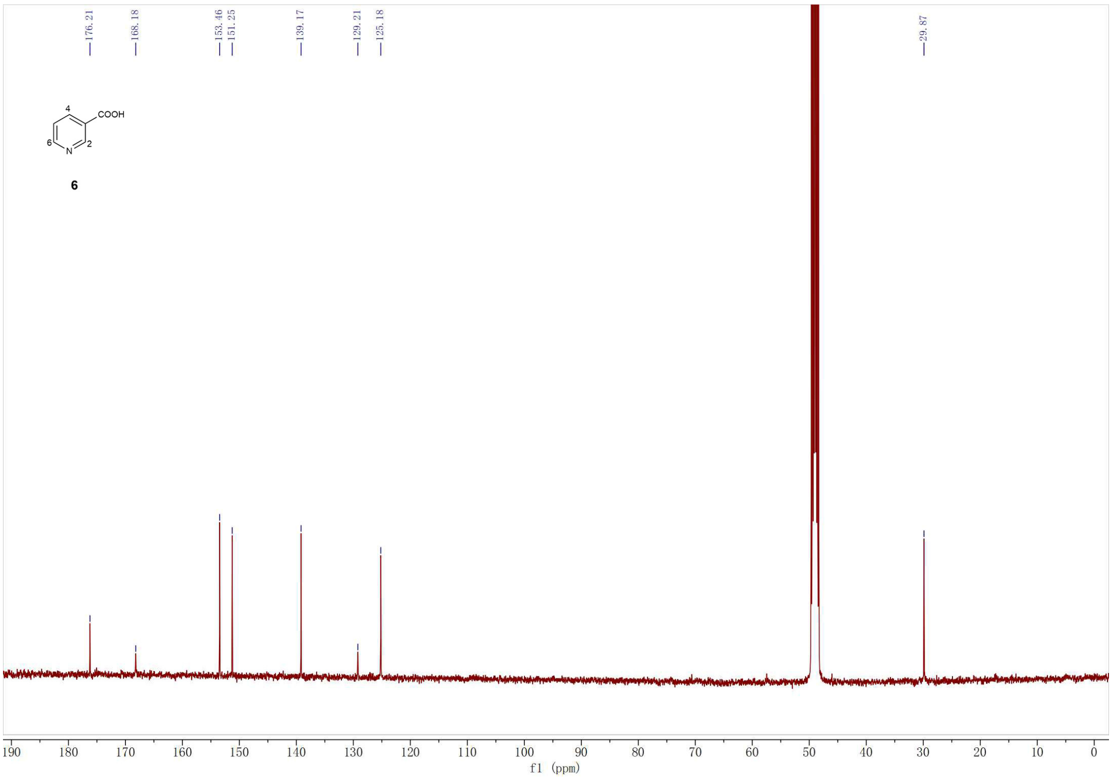

Figure S22. ^{13}C-NMR of (**6**) (400 MHz, $CDOD_3$)

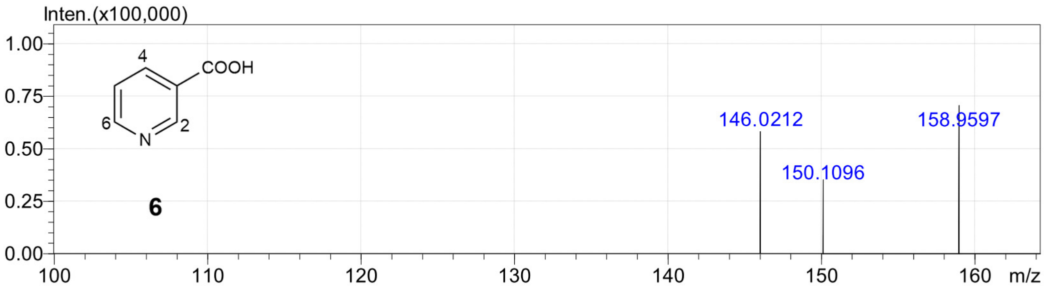

Figure S23. HR-ESI-MS of (**6**)

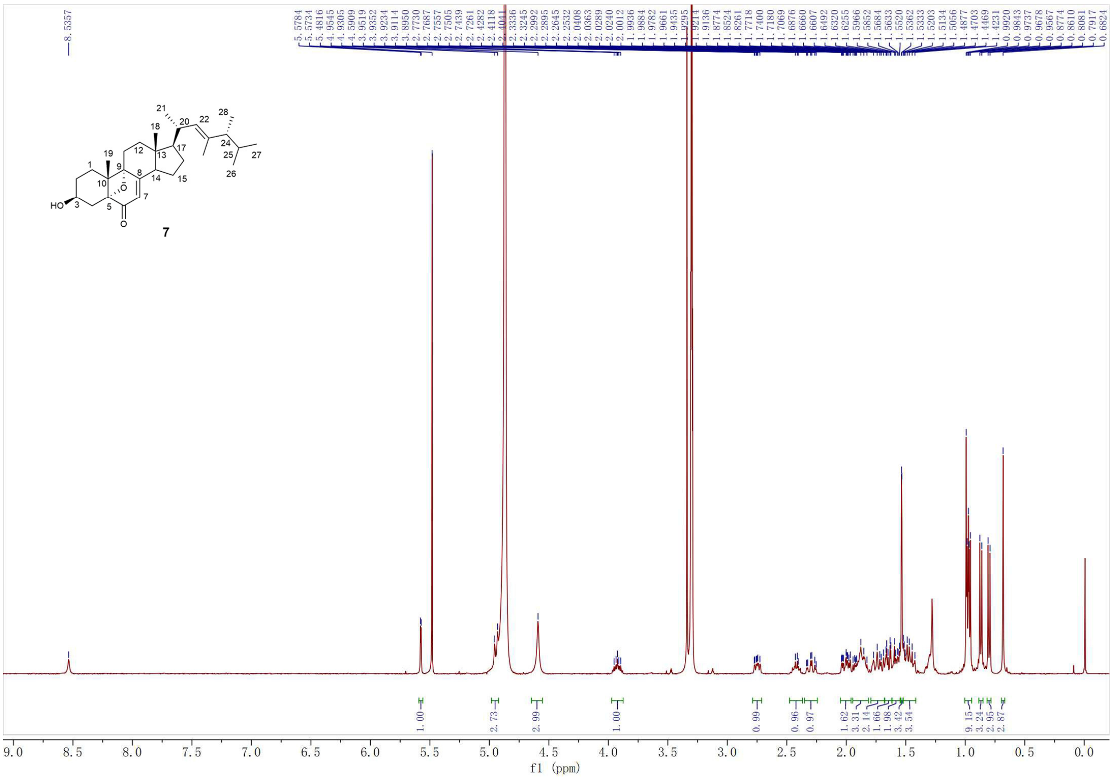

Figure S24. ^{1}H-NMR of (**7**) (400 MHz, CDOD$_3$)

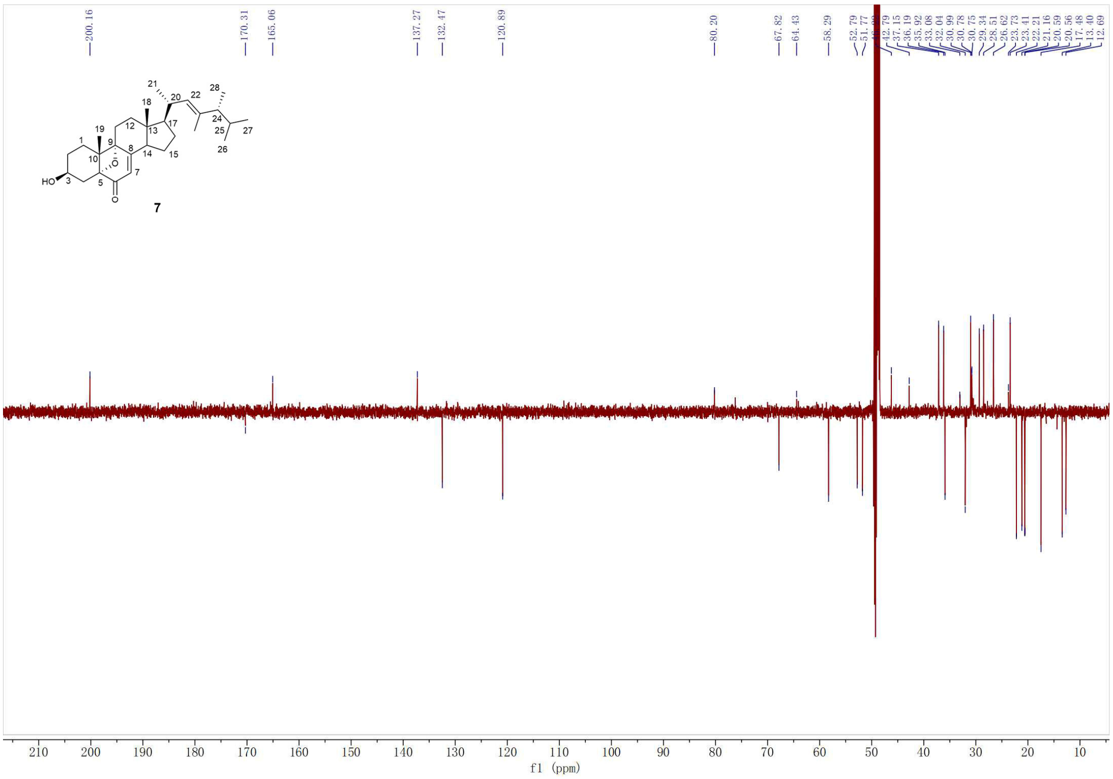

Figure S25. ^{13}C-NMR of (**7**) (400 MHz, CDOD$_3$)

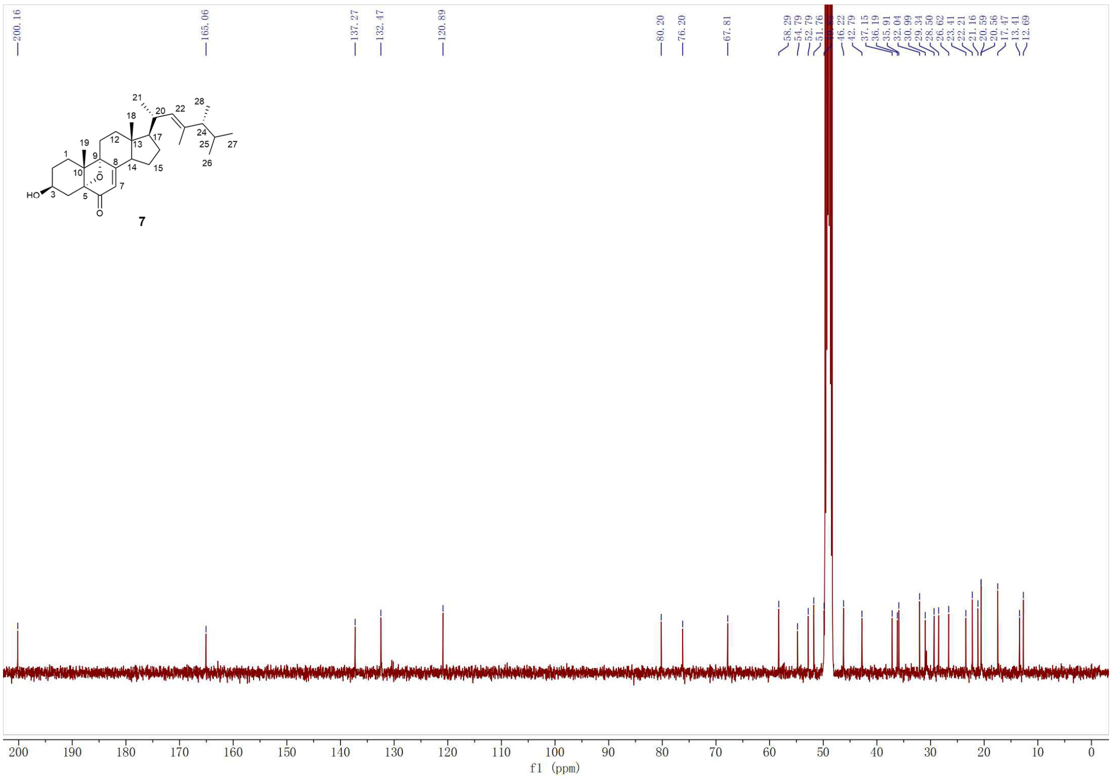

Figure S26. DEPT of (**7**) (400 MHz, CDOD3)

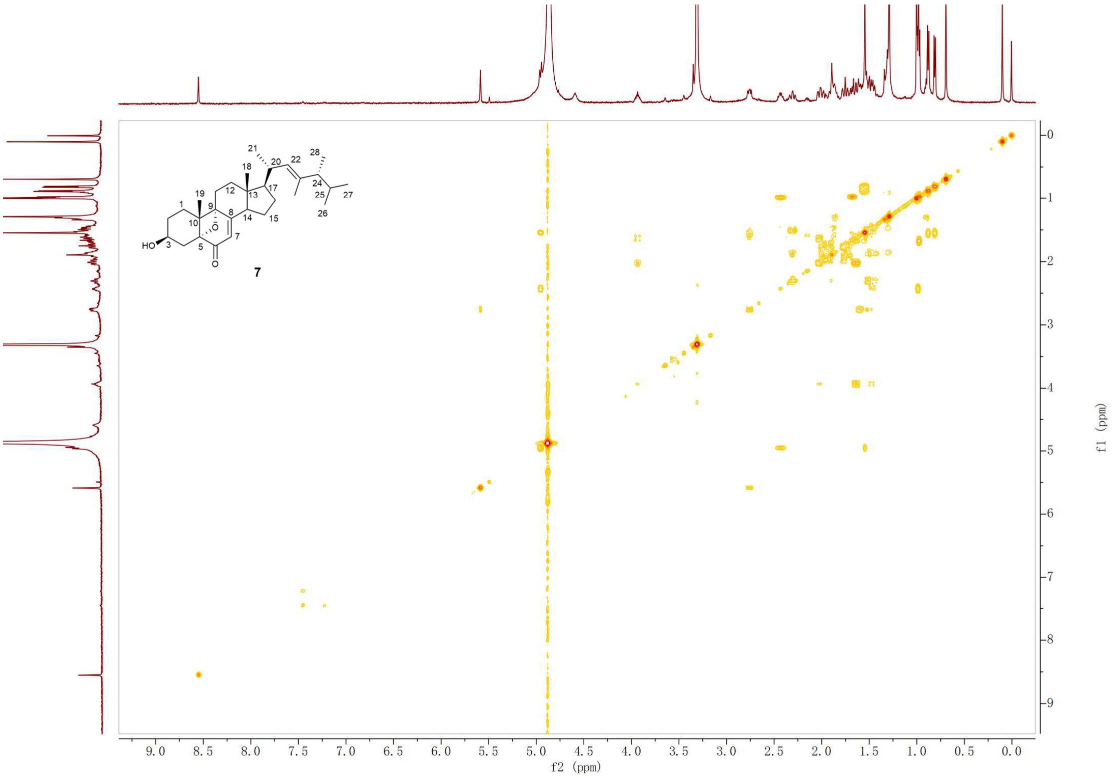

Figure S27. COSY of (**7**) (400 MHz, CDOD3)

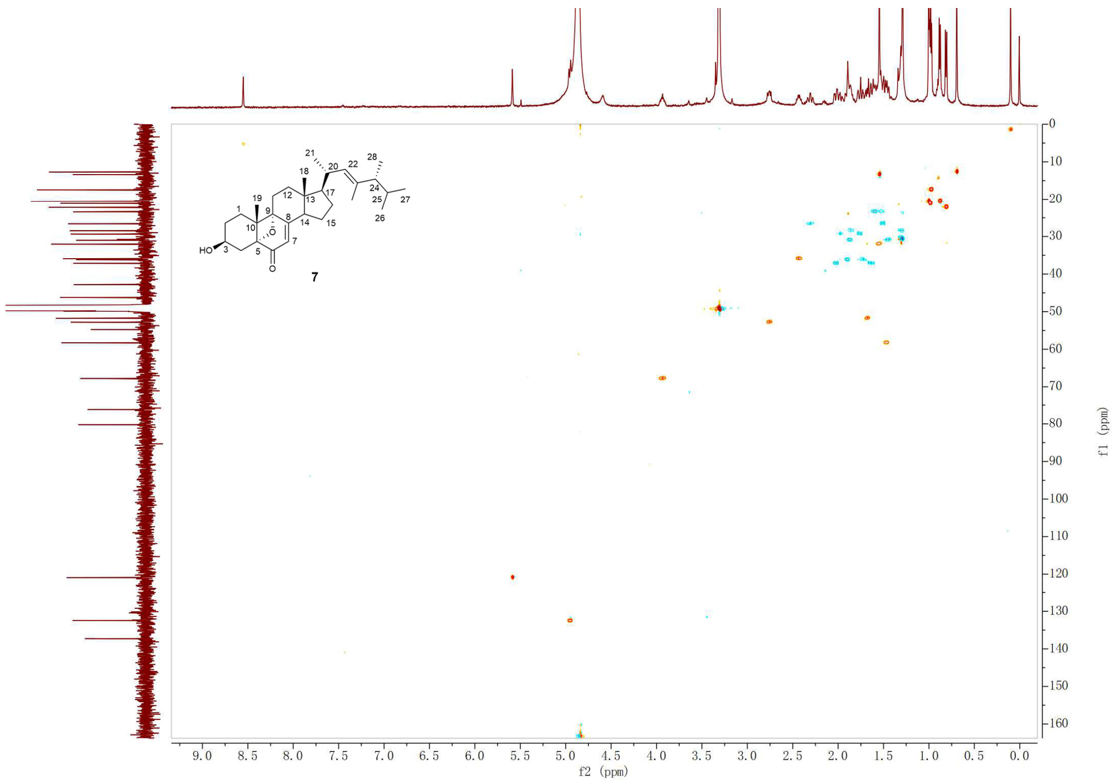

Figure S28.HSQC of (**7**) (400 MHz, CDOD3)

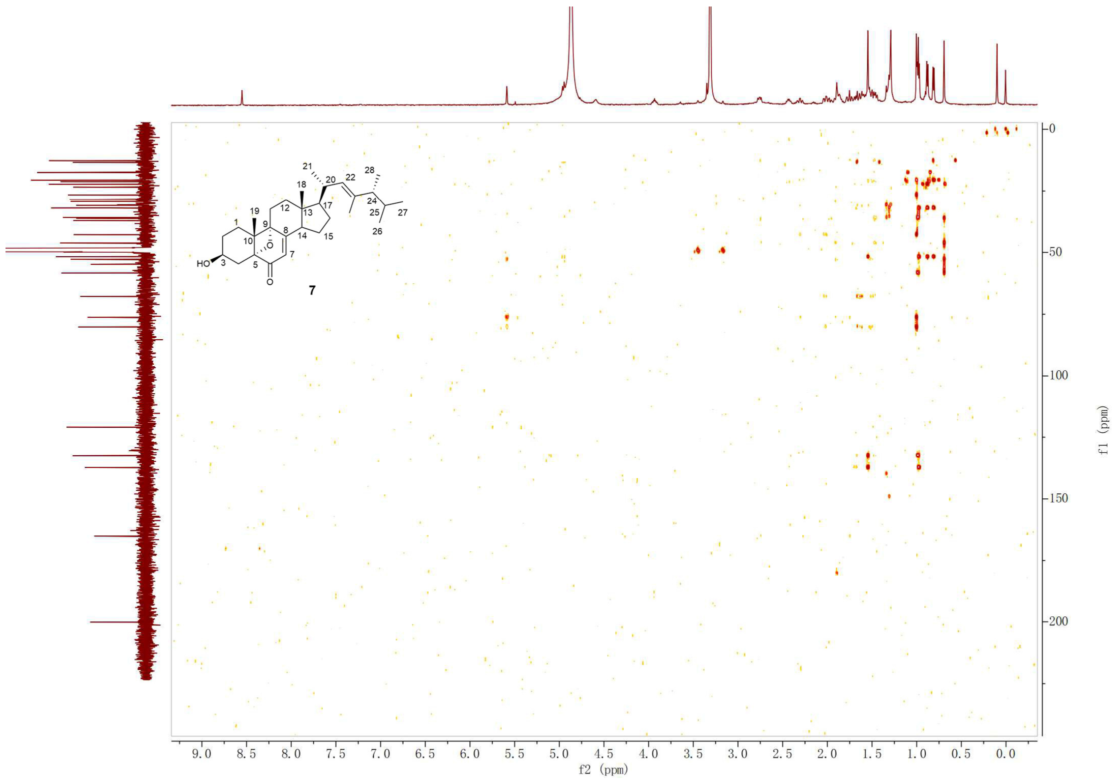

Figure S29.HMBC of (**7**) (400 MHz, CDOD3)

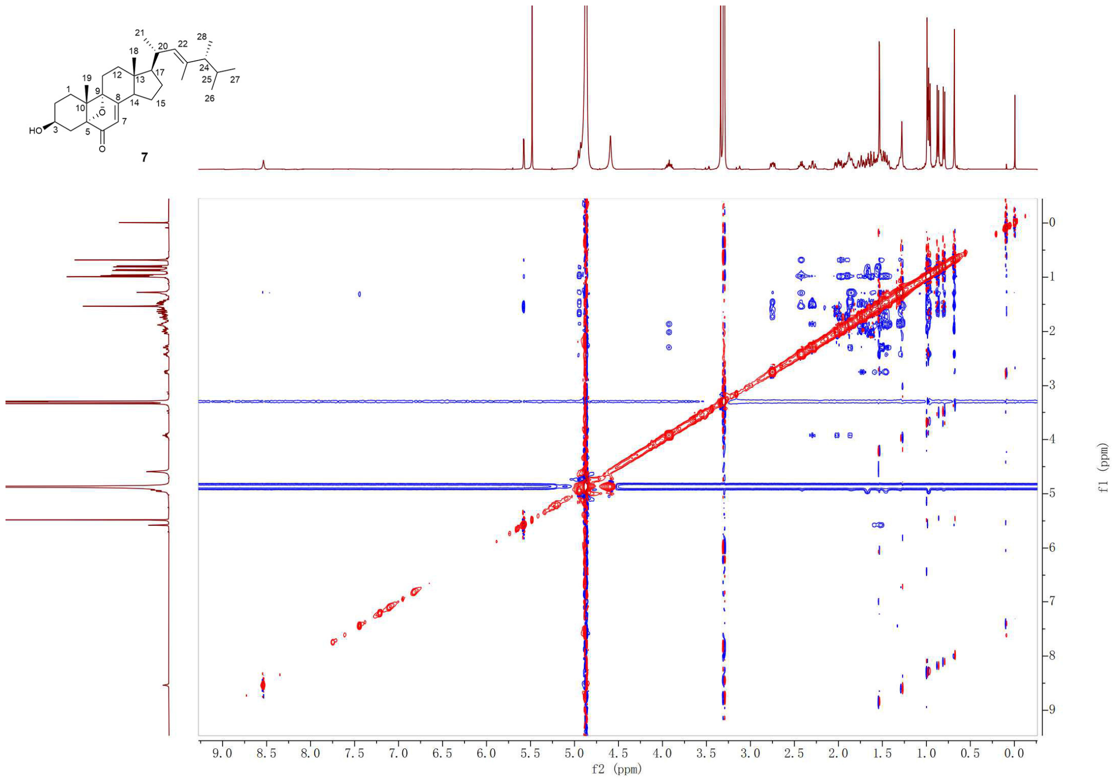

Figure S30.NOESY of (**7**) (400 MHz, CDOD3)

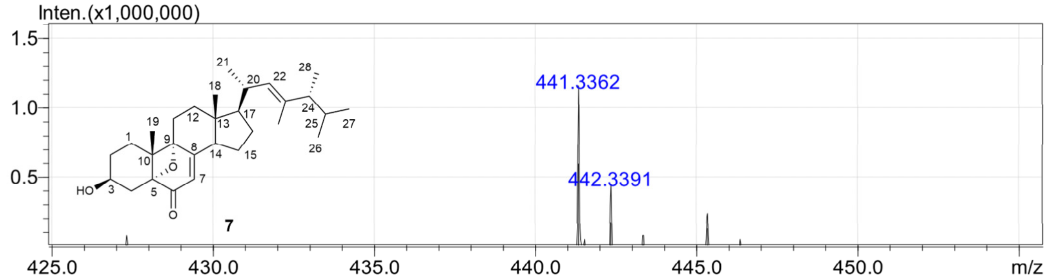

Figure S31. HR-ESI-MS of (**7**)

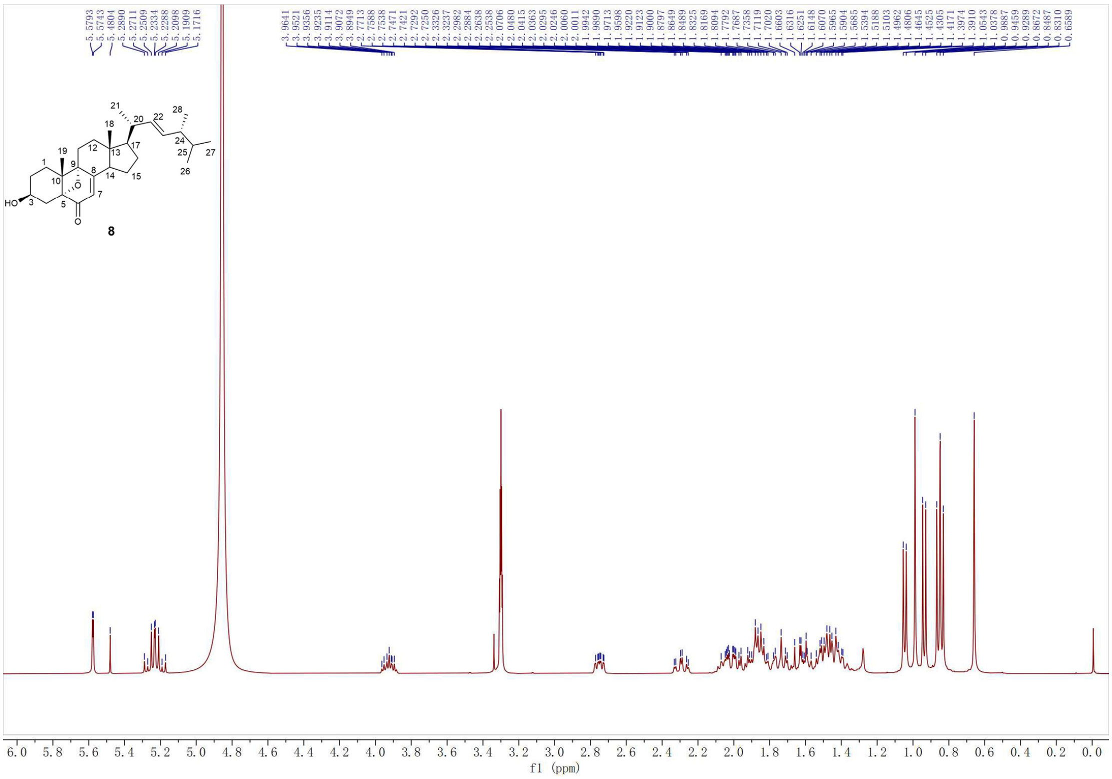

Figure S32. ^{1}H-NMR of (**8**) (400 MHz, CDOD$_3$)

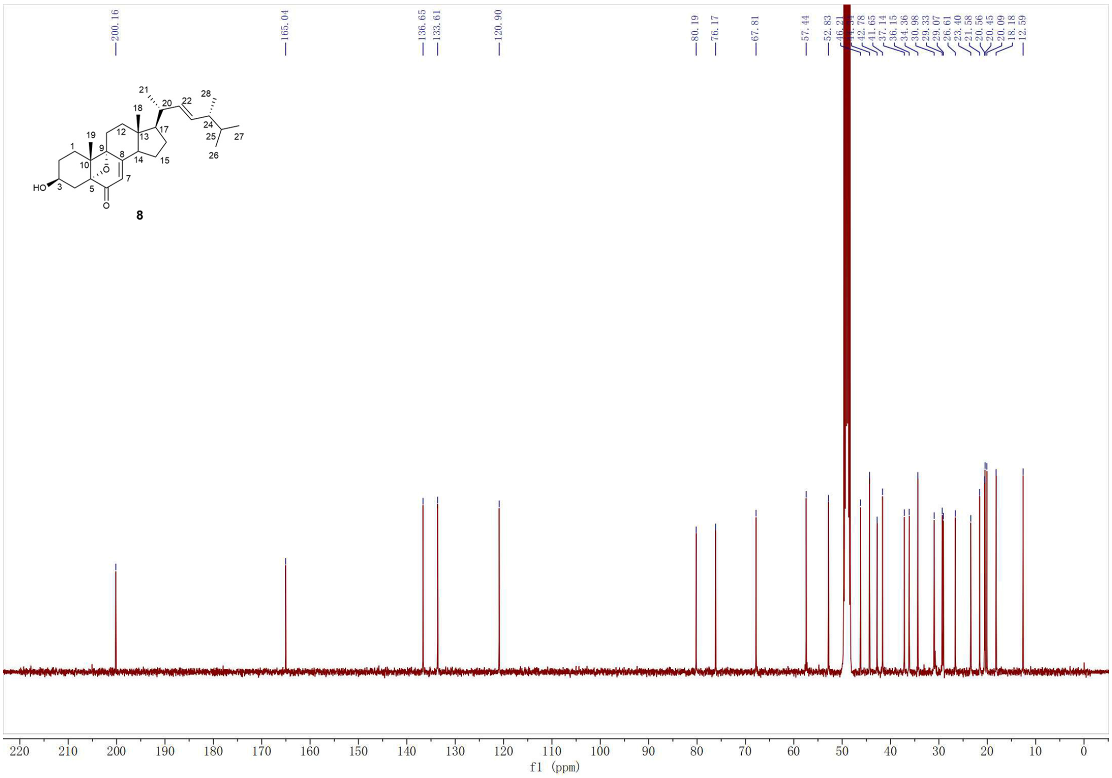

Figure S33. ^{13}C-NMR of (**8**) (400 MHz, CDOD$_3$)

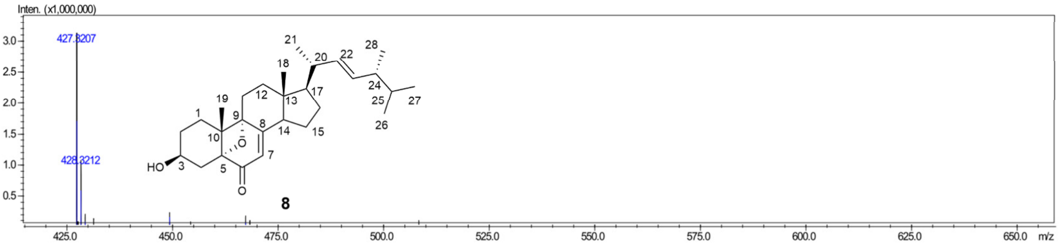

Figure S34. HR-ESI-MS of (**8**)

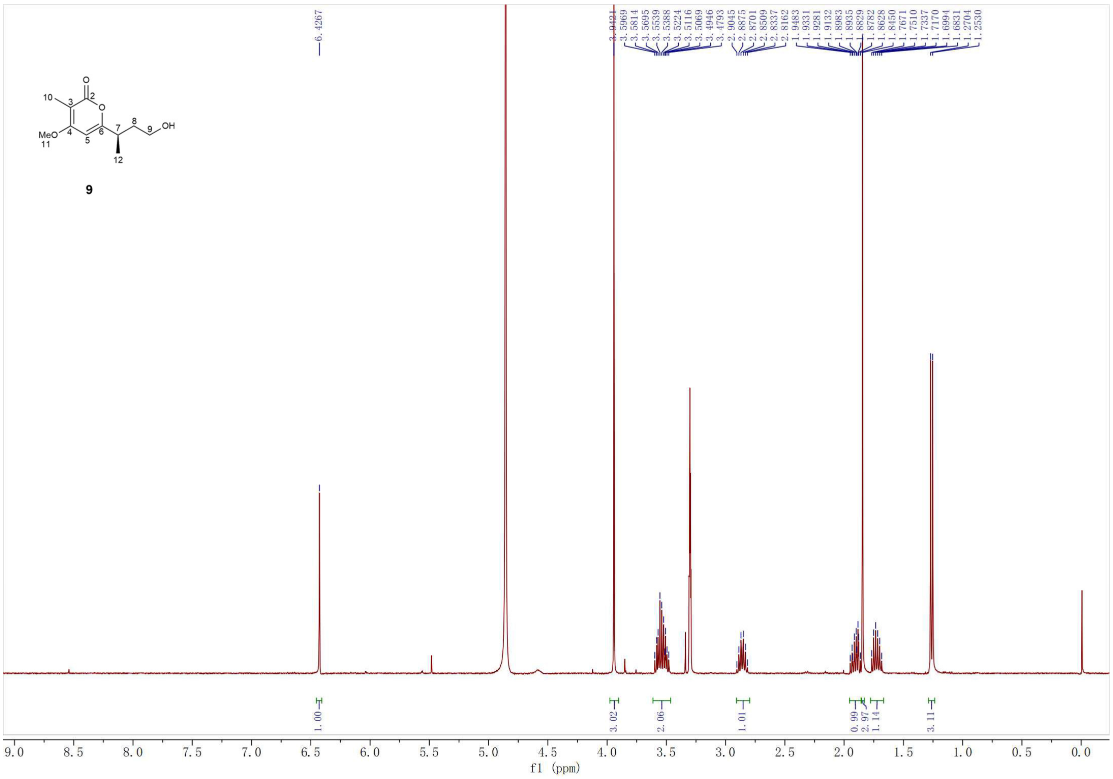

Figure S35. ^{1}H-NMR of (**9**) (400 MHz, $CDOD_3$)

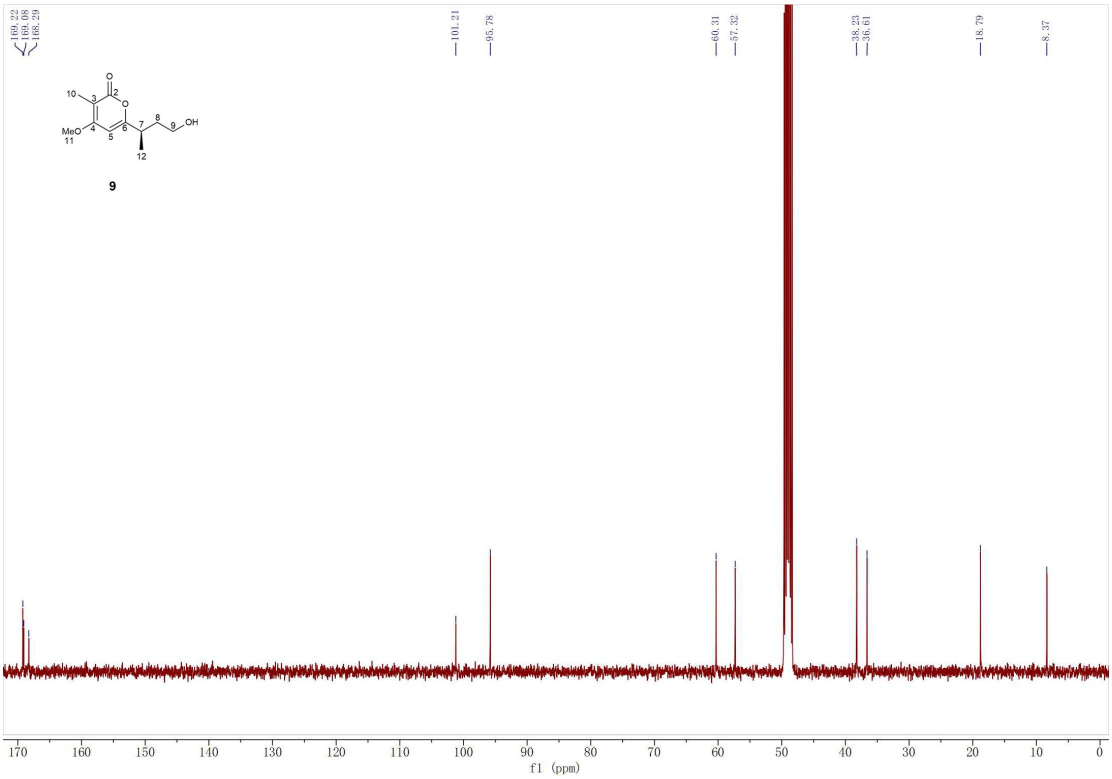

Figure S36. ^{13}C-NMR of (**9**) (400 MHz, CDOD$_3$)

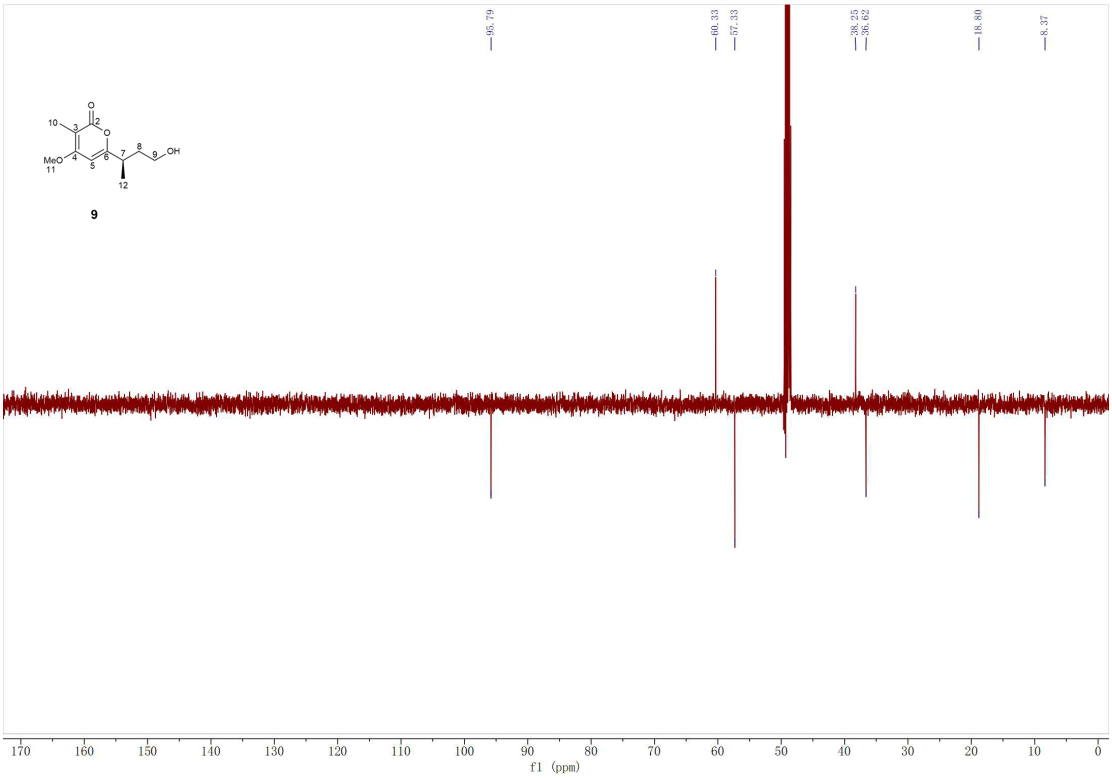

Figure S37. DEPT of (**9**) (400 MHz, CDOD$_3$)

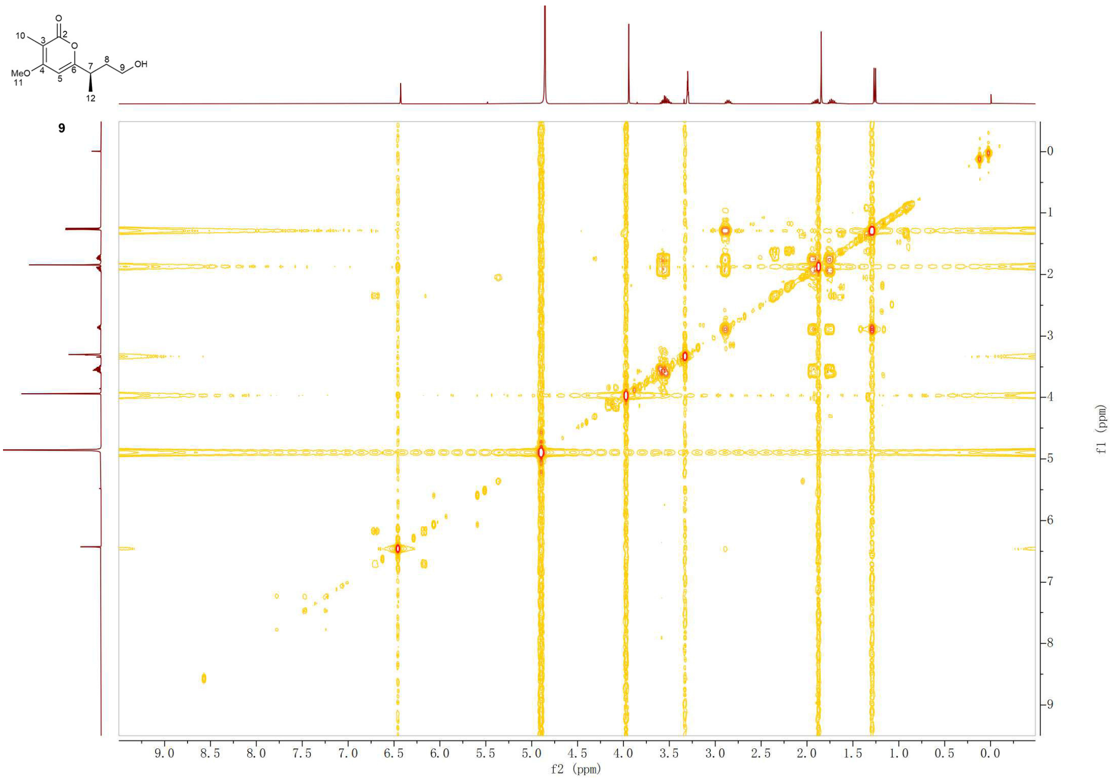

Figure S38. COSY of (**9**) (400 MHz, CDOD3)

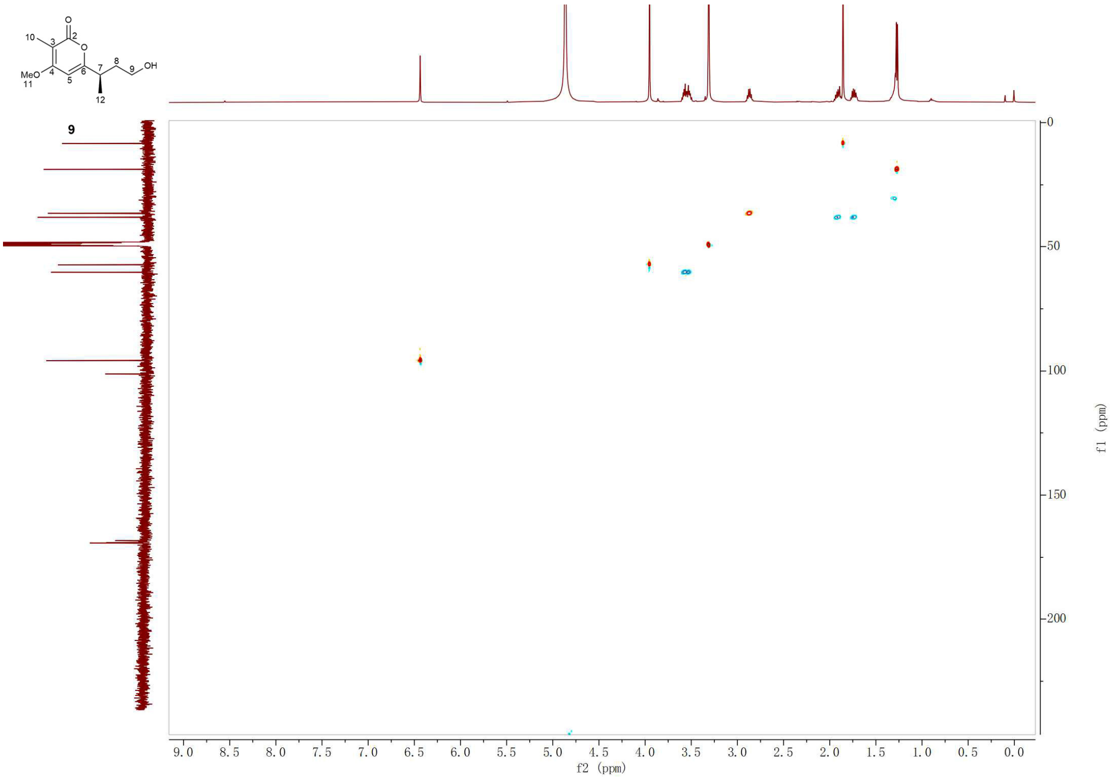

Figure S39. HSQC of (**9**) (400 MHz, CDOD3)

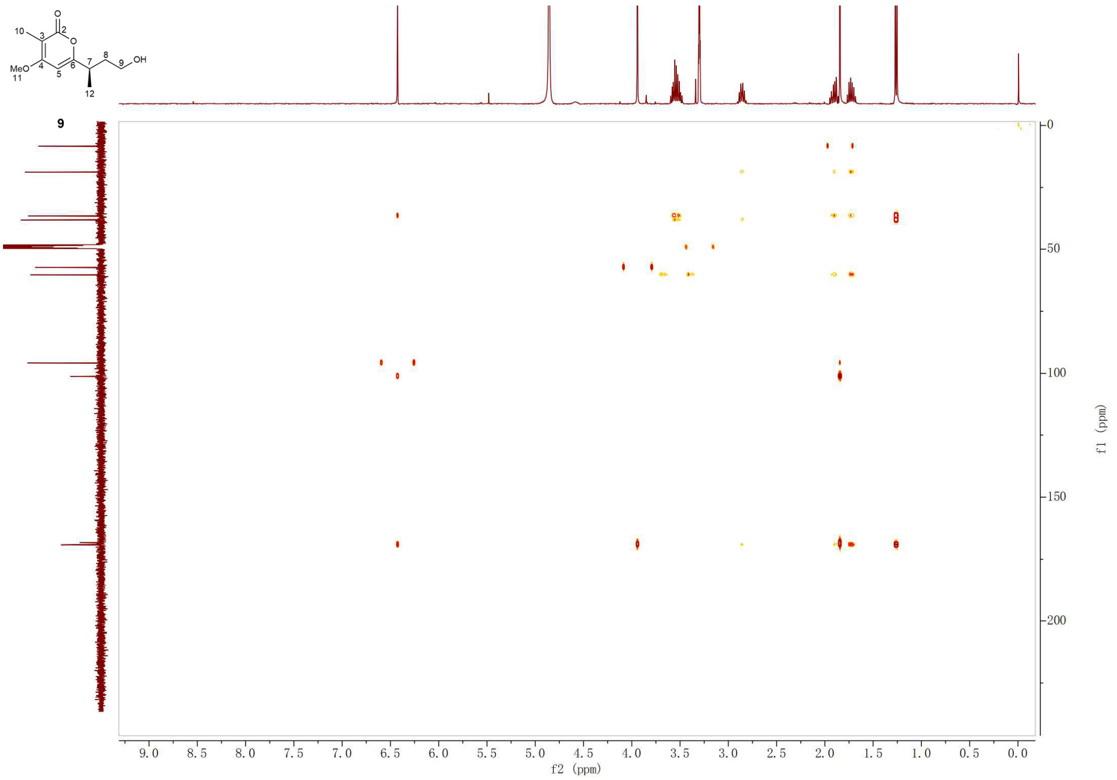

Figure S40. HMBC of (**9**) (400 MHz, CDOD3)

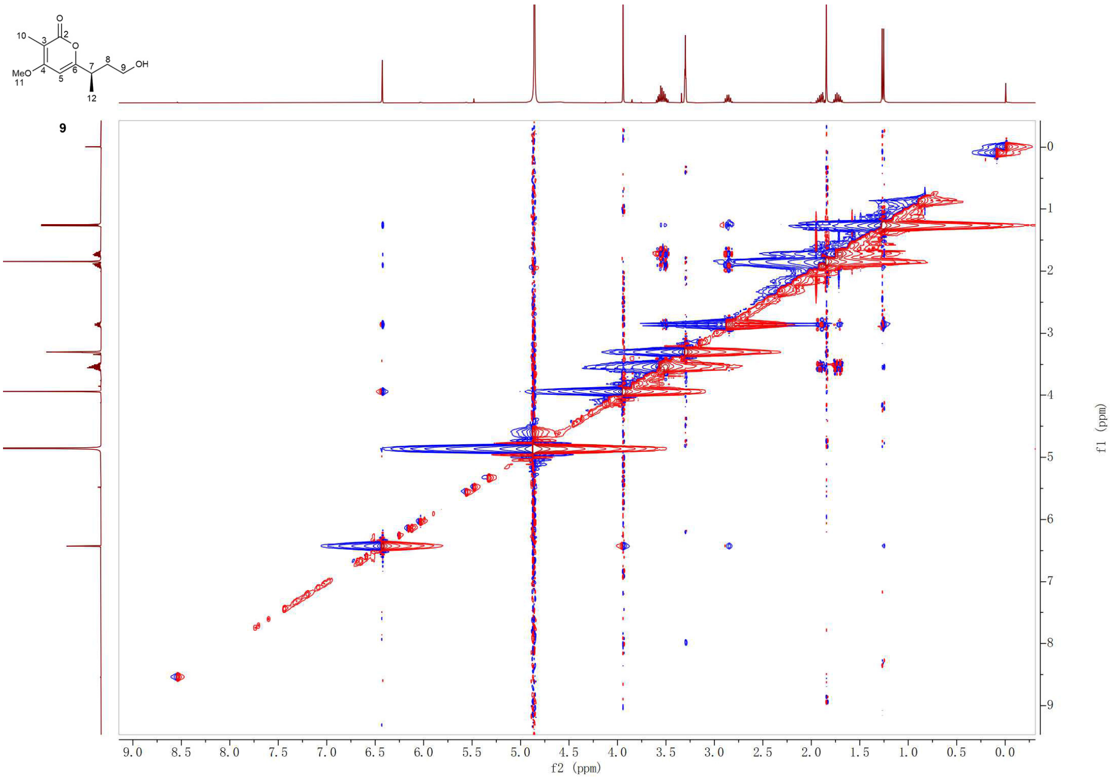

Figure S41. NOESY of (**9**) (400 MHz, CDOD3)

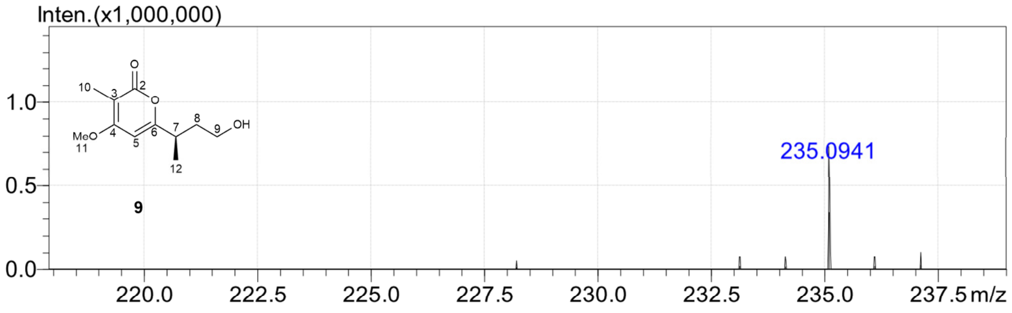

Figure S42. HR-ESI-MS of (**9**)

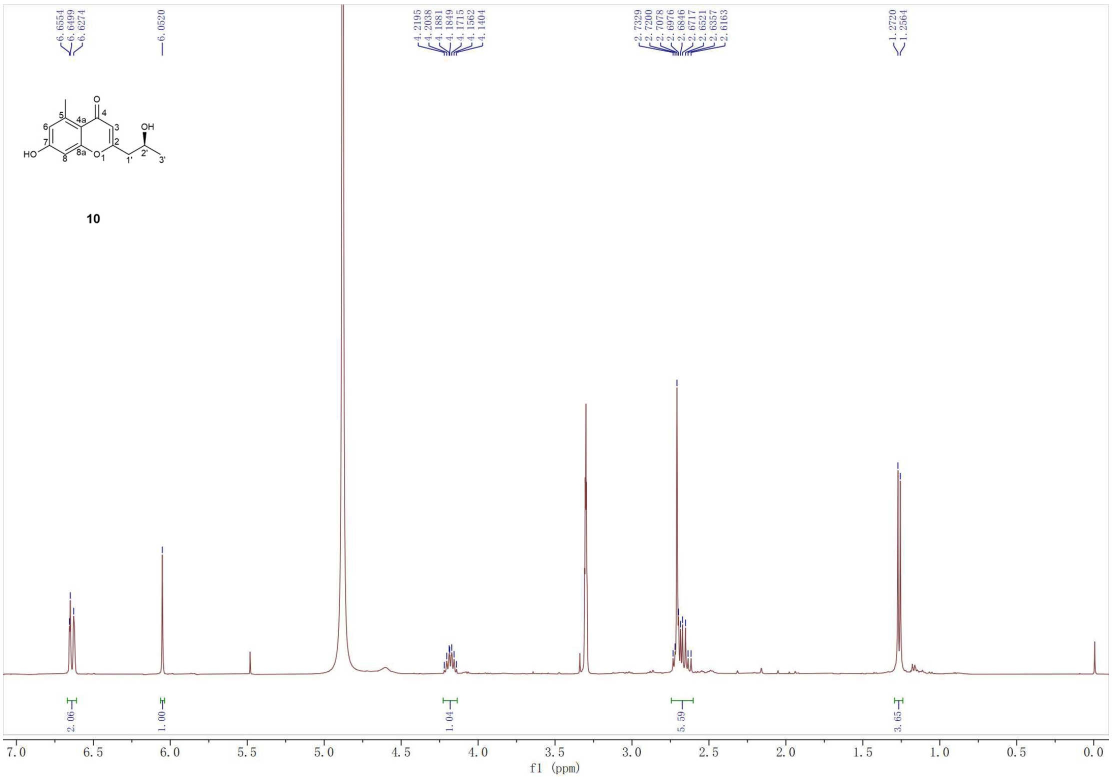

Figure S43. ^{1}H-NMR of (**10**) (400 MHz, $CDOD_3$)

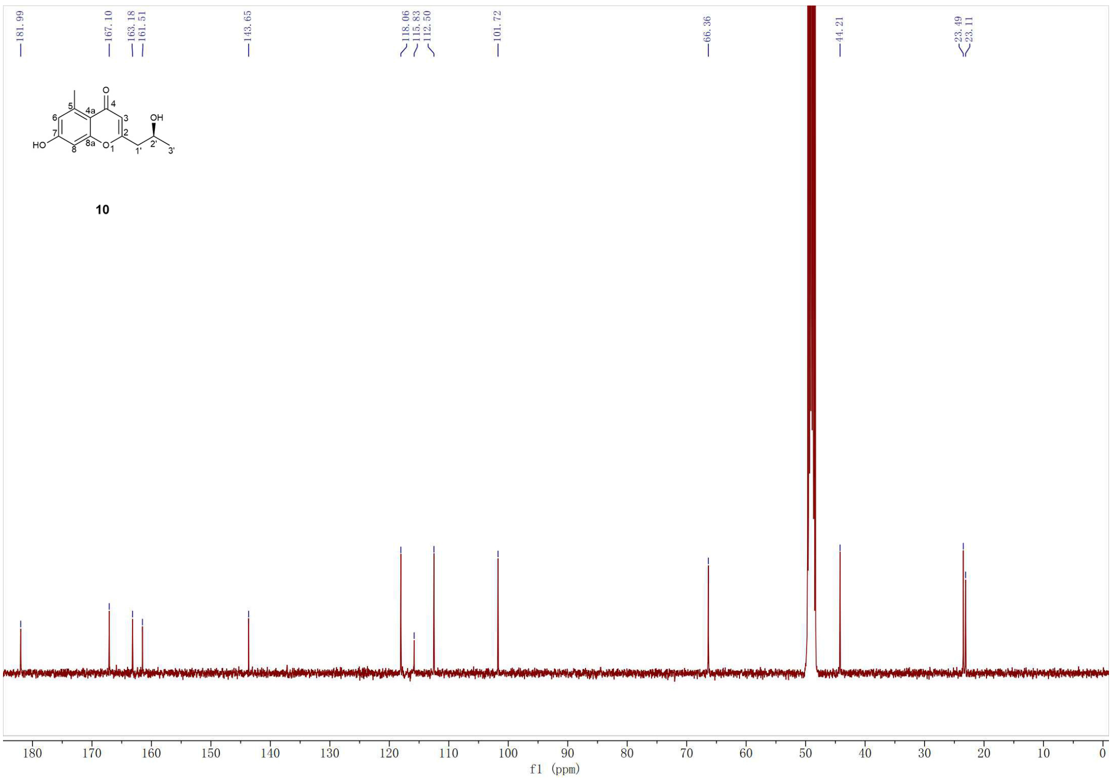

Figure S44. ^{13}C-NMR of (**10**) (400 MHz, $CDOD_3$)

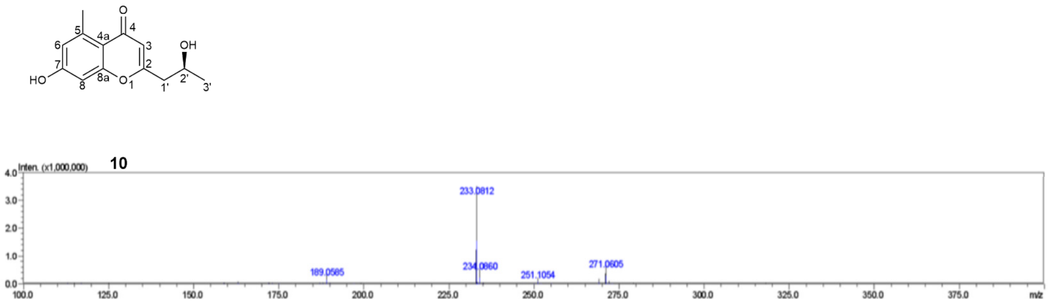

Figure S45. HR-ESI-MS of (**10**)

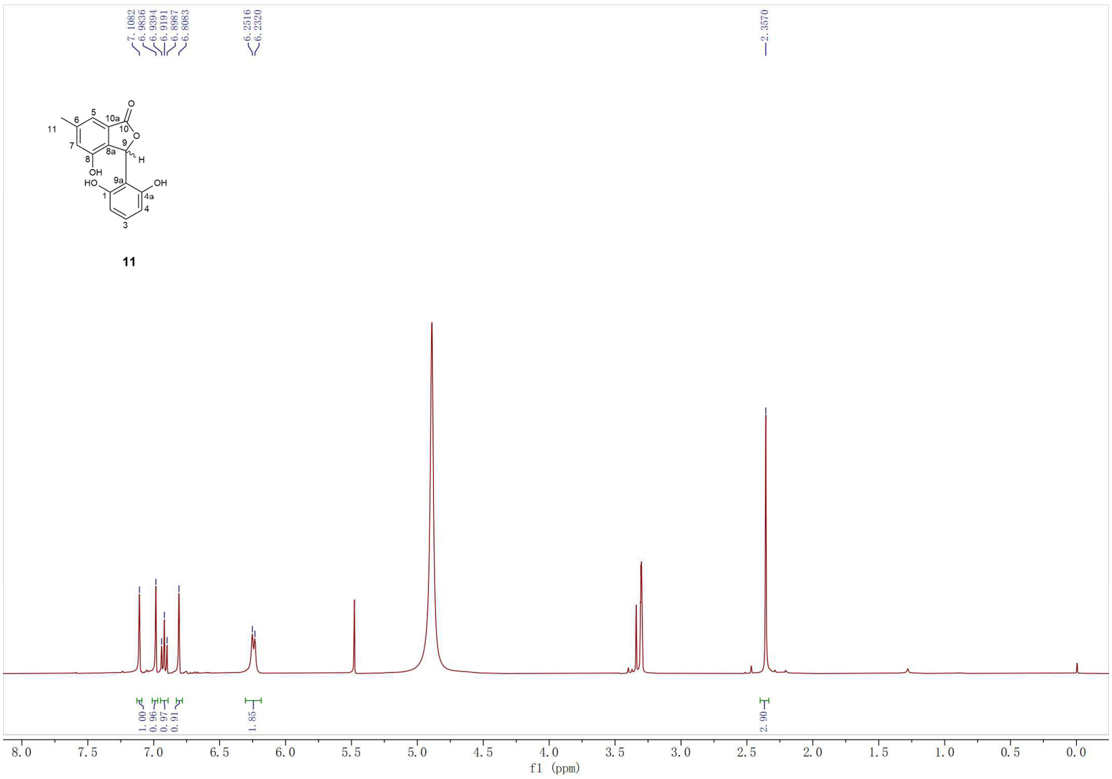

Figure S46. ^{1}H-NMR of (**11**) (400 MHz, CDOD$_3$)

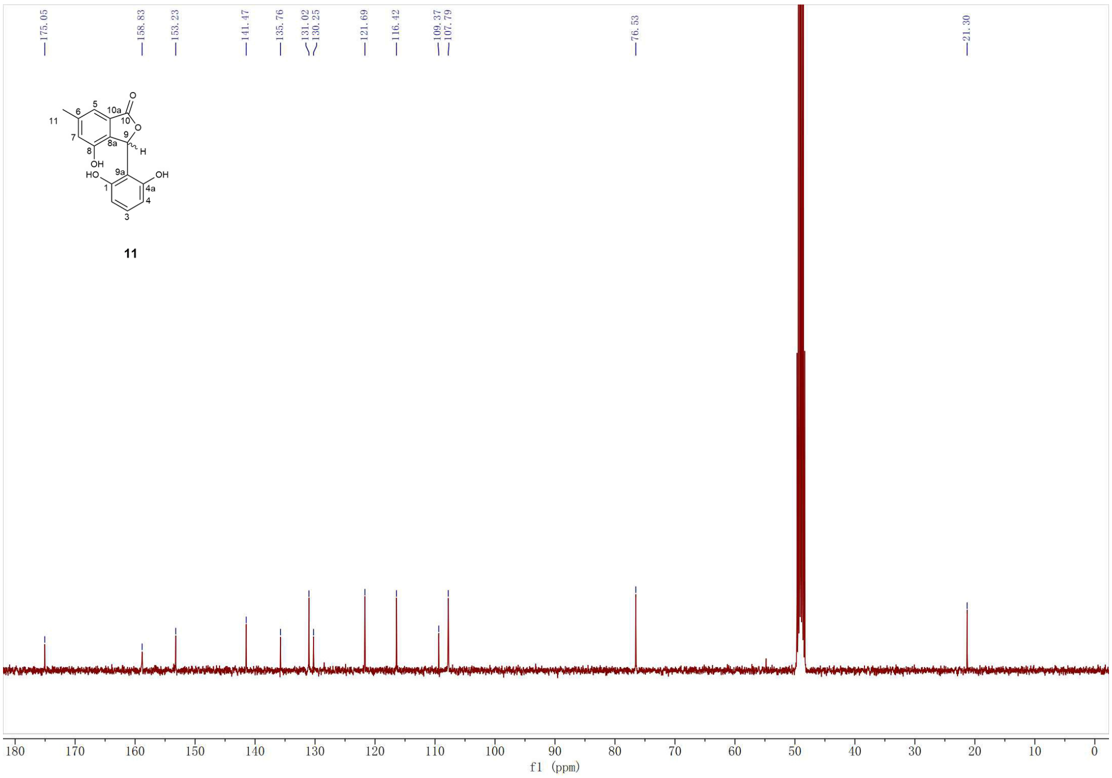

Figure S47. ^{13}C-NMR of (**11**) (400 MHz, CDOD$_3$)

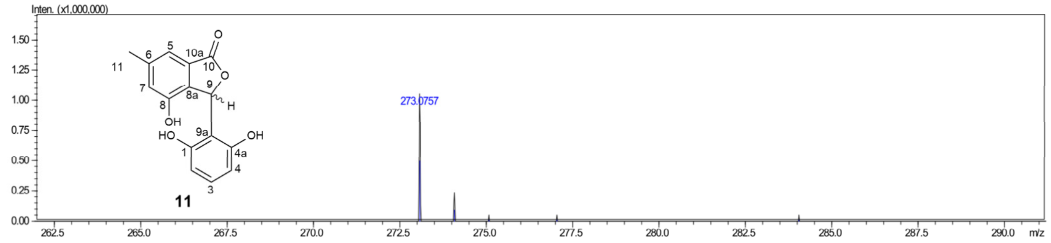

Figure S48. HR-ESI-MS of (**11**)

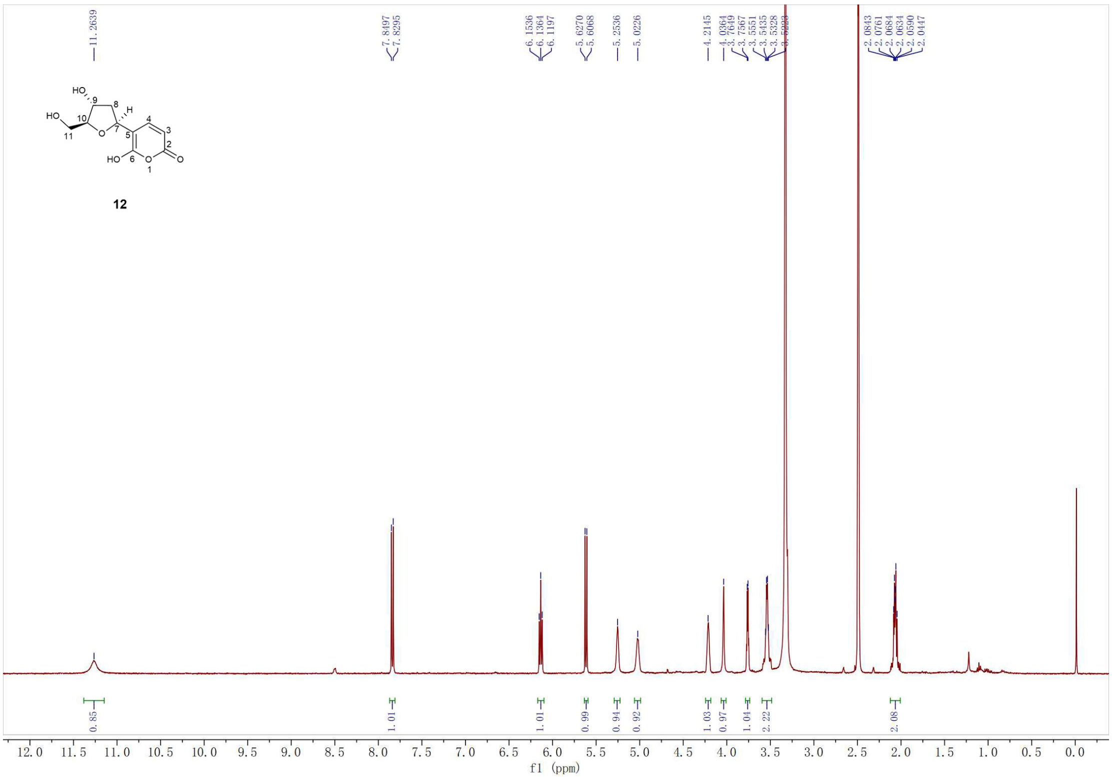

Figure S49. ^{1}H-NMR of (**12**) (400 MHz,DMSO)

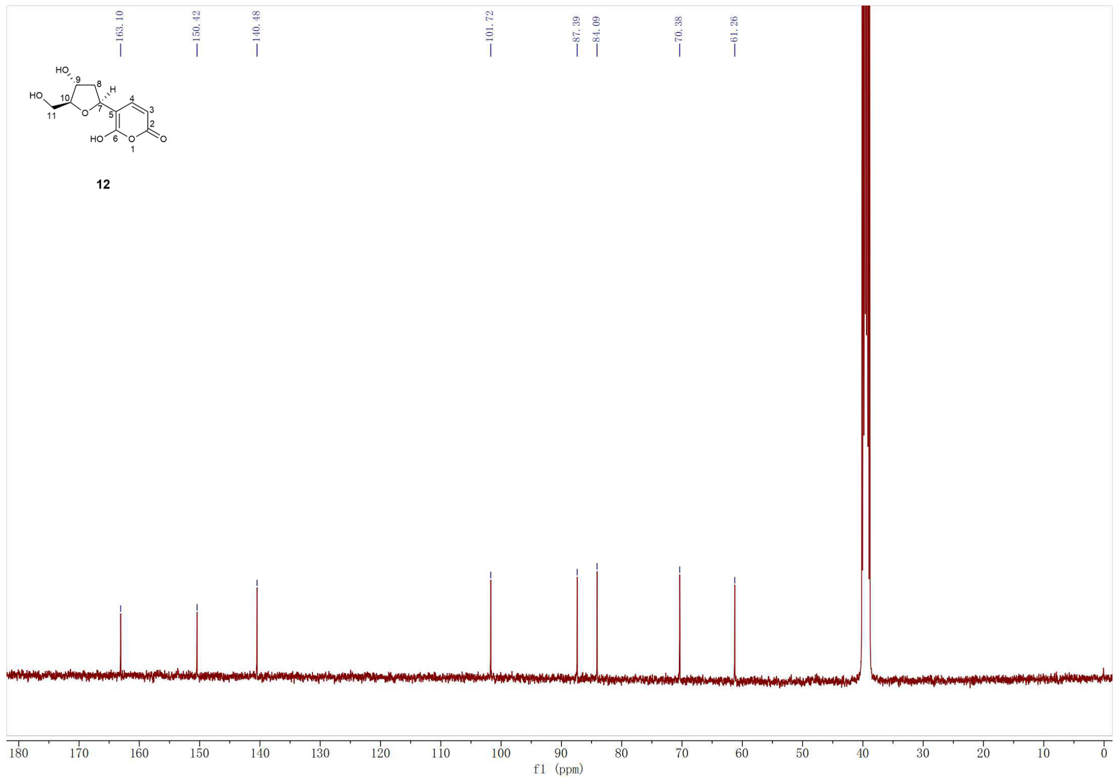

Figure S50. ^{13}C-NMR of (**12**) (400 MHz,DMSO)

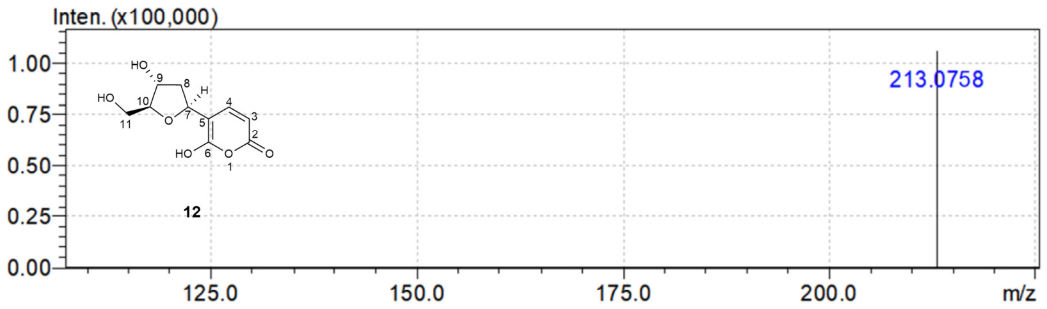

Figure S51. HR-ESI-MS of (**12**)

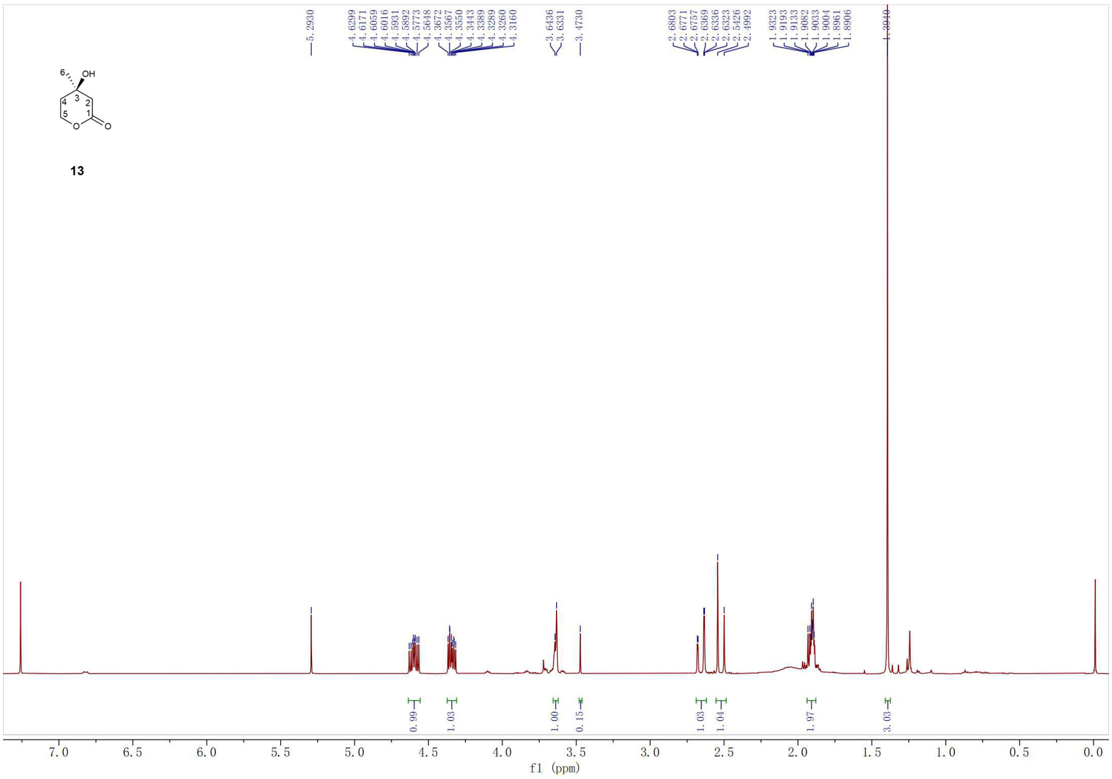

Figure S52. ^{1}H-NMR of (**13**) (400 MHz, $CDCl_3$)

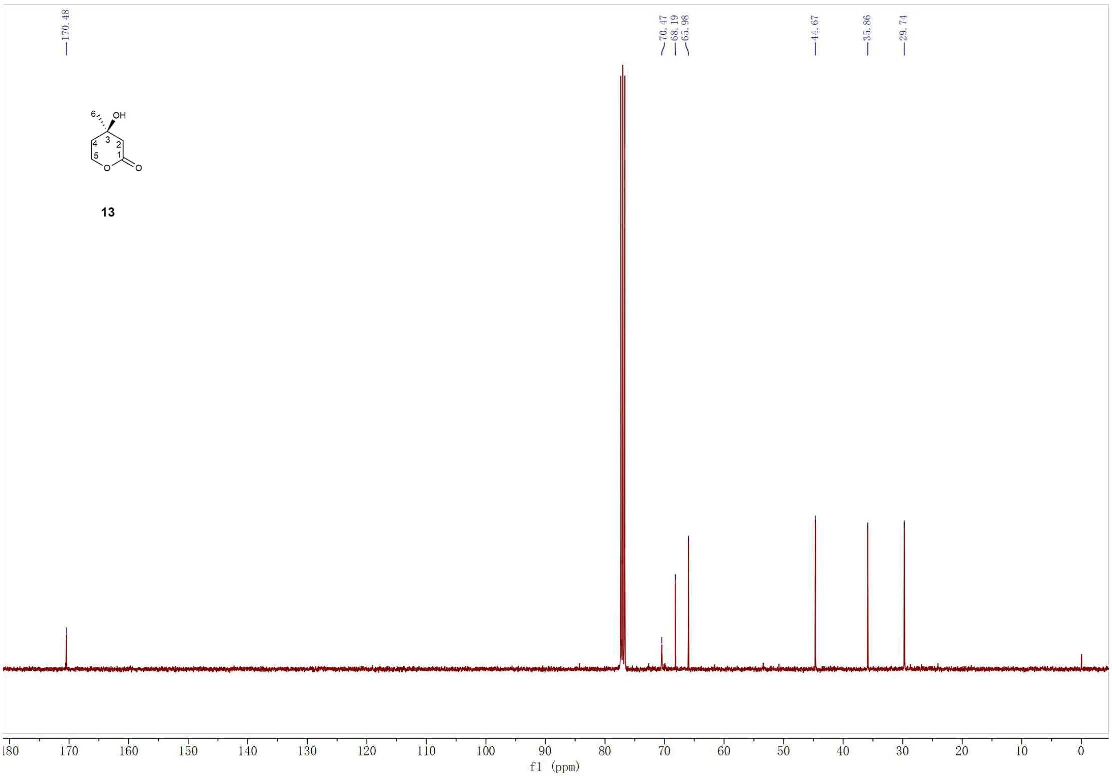

Figure S53. ^{13}C-NMR of (**13**) (400 MHz, $CDCl_3$)

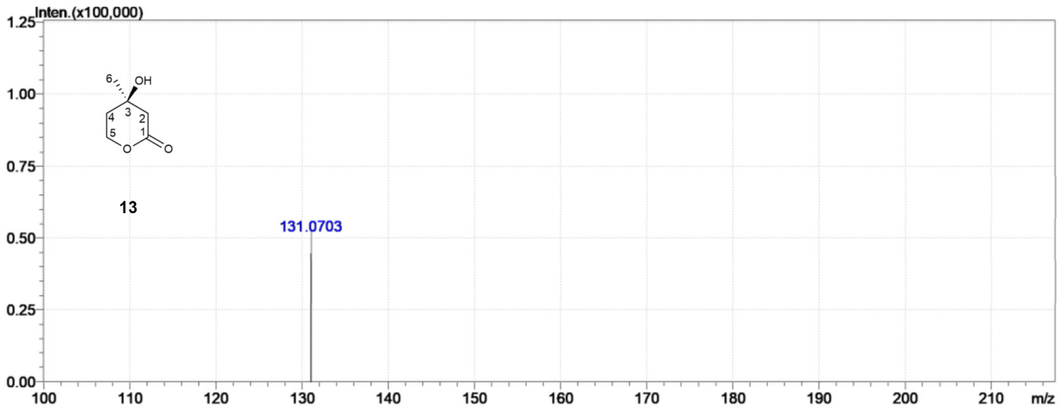

Figure S54. HR-ESI-MS of (**13**)

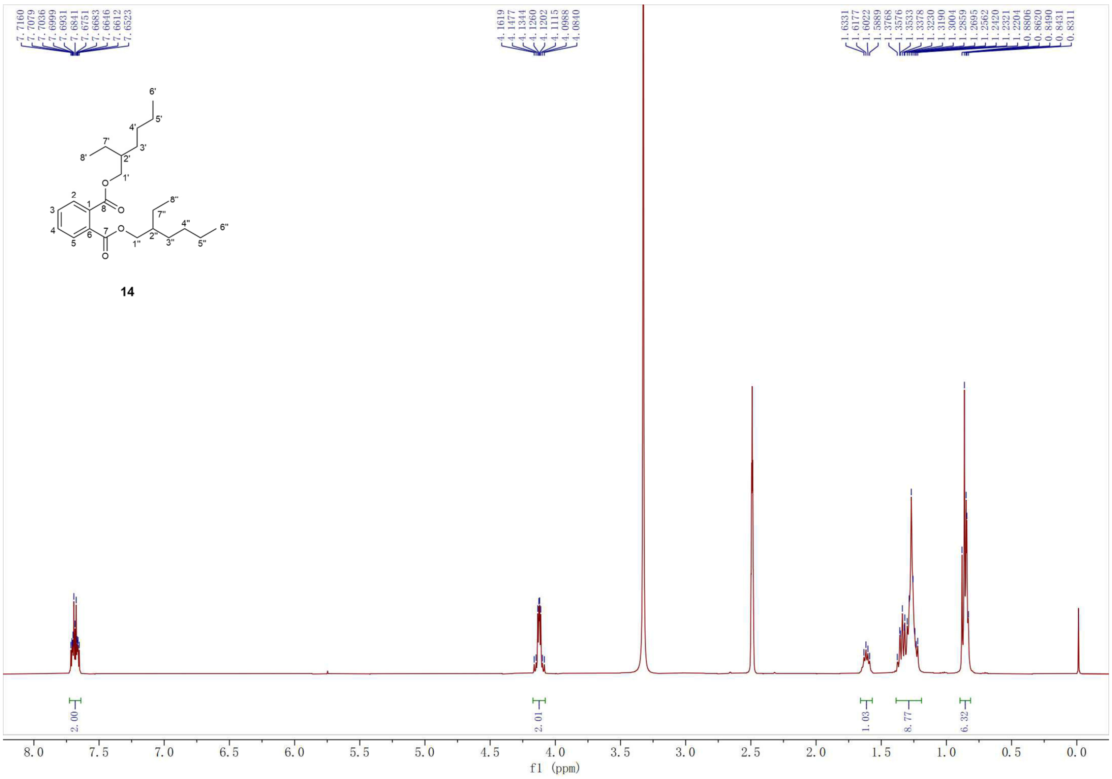

Figure S55. ^{1}H-NMR of (**14**) (400 MHz,DMSO)

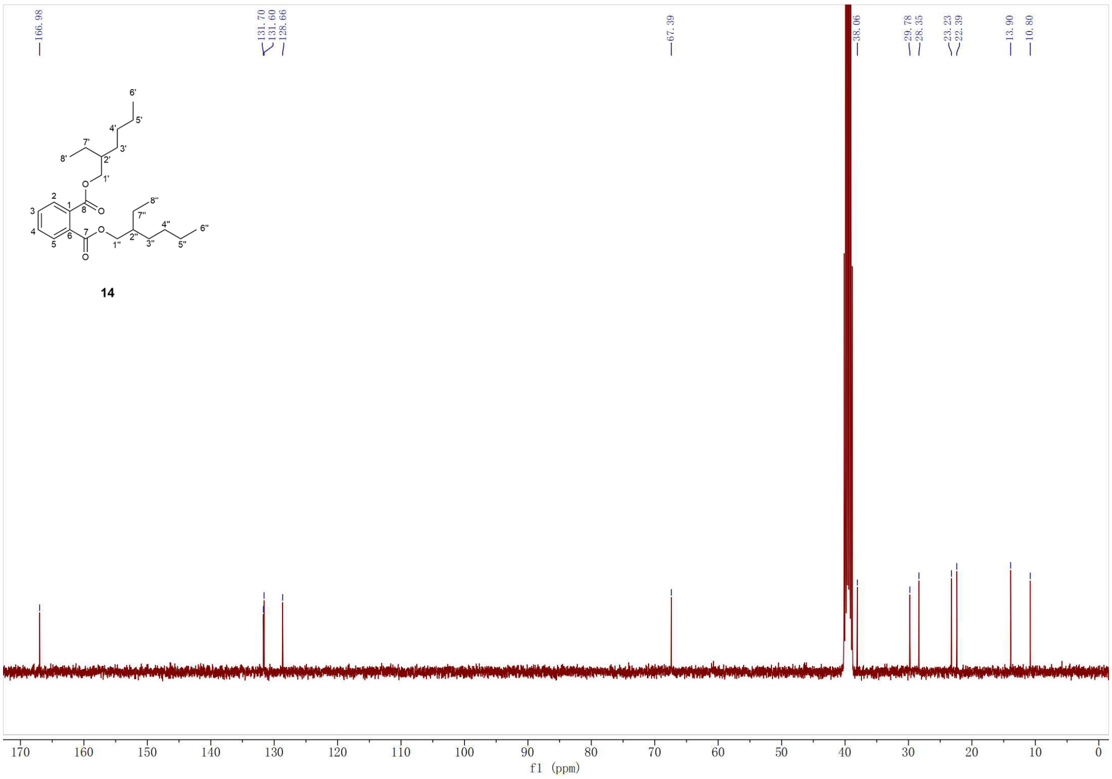

Figure S56. ^{13}C-NMR of (**14**) (400 MHz, DMSO)

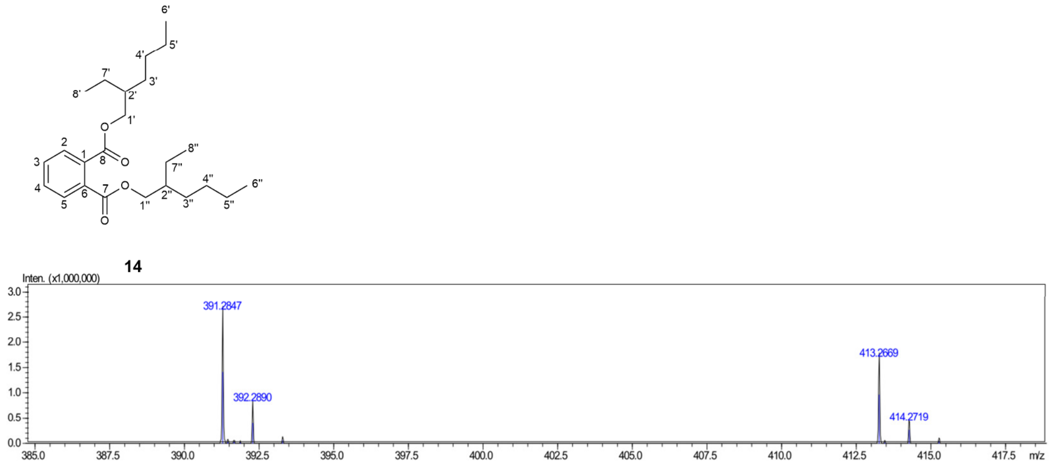

Figure S57. HR-ESI-MS of (**14**)

Table S1. Gibbs free energies[a] and equilibrium populations[b] of low-energy conformers of phomosterol C (**7**)

Conformers	In MeOH	
	G[a]	*P* (%)[b]
**7-1**	-851307.57415587	22.71
**7-2**	-851307.60427635	23.90
**7-3**	-851305.40171625	0.58

7-4	-851308.07365383	52.81

[a]B3LYP/6-31G(d,p), in kcal/mol. [b]From *G* values at 298.15K.

Table S2. Cartesian coordinates for the low-energy reoptimized MMFF conformers of phomosterol C (**7**) at B3LYP/6-31G(d,p) level of theory in gas

**7-1**		Standard Orientation (Ångstroms)			
Center number	Atomic number	Atomic Type	X	Y	Z
1.	6.	0.	6.063072	-1.614647	0.213802
2.	6.	0.	6.567810	-0.546186	-0.751418
3.	6.	0.	5.941446	0.813226	-0.425807
4.	6.	0.	4.441210	0.765091	-0.222181
5.	6.	0.	3.777587	-0.473567	0.477562
6.	6.	0.	4.539876	-1.763710	0.085690
7.	6.	0.	3.877779	2.081718	0.368291
8.	6.	0.	2.404136	2.144891	0.334197
9.	6.	0.	1.753436	1.050401	-0.104927
10.	6.	0.	2.626146	-0.122698	-0.565985
11.	6.	0.	0.283916	0.895123	-0.329325
12.	6.	0.	-0.274332	-0.478852	0.151093
13.	6.	0.	0.504731	-1.623626	-0.571419
14.	6.	0.	1.795832	-1.169663	-1.301024
15.	6.	0.	-0.713257	1.937533	0.184062
16.	6.	0.	-2.079513	1.198029	0.103725
17.	6.	0.	-1.780092	-0.297736	-0.237751
18.	6.	0.	-2.826025	-1.261520	0.376797
19.	6.	0.	-2.546416	-2.747816	0.067396
20.	6.	0.	-4.212553	-0.921470	-0.131824
21.	6.	0.	-5.341194	-0.706356	0.563833
22.	6.	0.	-6.691960	-0.460898	-0.116608
23.	6.	0.	-7.120439	1.043109	-0.041877
24.	6.	0.	-8.621313	1.226145	-0.318256
25.	6.	0.	-6.282168	1.961753	-0.942052
26.	6.	0.	-6.796305	-1.043131	-1.535258
27.	6.	0.	3.559595	-0.435144	1.988766
28.	6.	0.	-0.127899	-0.628632	1.679557
29.	8.	0.	7.988934	-0.500746	-0.634168
30.	8.	0.	4.593025	2.963150	0.820131
31.	8.	0.	3.662474	0.464588	-1.429053
32.	6.	0.	-5.412035	-0.729364	2.074937
33.	1.	0.	6.358839	-1.339171	1.234243
34.	1.	0.	6.556577	-2.565583	-0.012752
35.	1.	0.	6.281452	-0.841147	-1.773945
36.	1.	0.	6.393724	1.196467	0.495293
37.	1.	0.	6.164009	1.549096	-1.209823
38.	1.	0.	4.184113	-2.596833	0.702950
39.	1.	0.	4.326050	-2.025694	-0.953890
40.	1.	0.	1.914518	3.063236	0.643407
41.	1.	0.	0.173990	0.875618	-1.427389
42.	1.	0.	-0.135385	-2.111269	-1.313055
43.	1.	0.	0.763027	-2.400790	0.156512
44.	1.	0.	2.404859	-2.044258	-1.539946
45.	1.	0.	1.537858	-0.717931	-2.267024
46.	1.	0.	-0.478981	2.227005	1.213859
47.	1.	0.	-0.694330	2.853361	-0.413855
48.	1.	0.	-2.619348	1.272039	1.053243
49.	1.	0.	-2.732908	1.642551	-0.651749

50.	1.	0.	-1.834258	-0.416151	-1.330989
51.	1.	0.	-2.794814	-1.134894	1.464183
52.	1.	0.	-3.322470	-3.377582	0.512688
53.	1.	0.	-2.554064	-2.933192	-1.013176
54.	1.	0.	-1.580009	-3.079742	0.456818
55.	1.	0.	-4.278350	-0.915832	-1.219844
56.	1.	0.	-7.430533	-1.001712	0.495515
57.	1.	0.	-6.949676	1.363924	0.994518
58.	1.	0.	-8.922630	2.265188	-0.146600
59.	1.	0.	-8.877038	0.981844	-1.355079
60.	1.	0.	-9.229839	0.590848	0.335130
61.	1.	0.	-5.211702	1.823185	-0.765524
62.	1.	0.	-6.477731	1.776091	-2.003896
63.	1.	0.	-6.522450	3.012443	-0.747481
64.	1.	0.	-7.818447	-0.962146	-1.915623
65.	1.	0.	-6.142805	-0.532647	-2.248552
66.	1.	0.	-6.523406	-2.102538	-1.536704
67.	1.	0.	3.030684	0.457746	2.326385
68.	1.	0.	3.000508	-1.315182	2.323128
69.	1.	0.	4.527016	-0.457515	2.501982
70.	1.	0.	-0.668920	0.146931	2.229461
71.	1.	0.	-0.516696	-1.596226	2.011004
72.	1.	0.	0.918326	-0.576116	1.985691
73.	1.	0.	8.310910	0.194704	-1.223125
74.	1.	0.	-4.433825	-0.796912	2.553439
75.	1.	0.	-5.908562	0.166500	2.465668
76.	1.	0.	-6.009182	-1.585674	2.415640

**7-2**		Standard Orientation (Ångstroms)			
Center number	Atom number	Type	X	Y	Z
1.	6.	0.	6.017683	-1.661828	0.118168
2.	6.	0.	6.524299	-0.569405	-0.829657
3.	6.	0.	5.919209	0.783318	-0.466480
4.	6.	0.	4.423265	0.741135	-0.237249
5.	6.	0.	3.752635	-0.506237	0.442015
6.	6.	0.	4.491482	-1.794907	0.005954
7.	6.	0.	3.883234	2.047163	0.398115
8.	6.	0.	2.409764	2.130513	0.383355
9.	6.	0.	1.738972	1.055633	-0.072818
10.	6.	0.	2.590950	-0.115938	-0.575353
11.	6.	0.	0.264459	0.923529	-0.279400
12.	6.	0.	-0.303524	-0.455457	0.174809
13.	6.	0.	0.448745	-1.589975	-0.590587
14.	6.	0.	1.736809	-1.133797	-1.324410
15.	6.	0.	-0.711371	1.965468	0.275175
16.	6.	0.	-2.087394	1.242795	0.208280
17.	6.	0.	-1.812484	-0.244792	-0.183982
18.	6.	0.	-2.862679	-1.213588	0.415330
19.	6.	0.	-2.612472	-2.691755	0.048967
20.	6.	0.	-4.250864	-0.827170	-0.051255
21.	6.	0.	-5.356324	-0.596923	0.673973
22.	6.	0.	-6.701001	-0.304541	-0.010788
23.	6.	0.	-6.674993	0.986321	-0.877381

24.	6.	0.	-6.290068	2.212661	-0.035532
25.	6.	0.	-8.012014	1.243603	-1.590696
26.	6.	0.	-7.181736	-1.547346	-0.787546
27.	6.	0.	3.557663	-0.502620	1.956904
28.	6.	0.	-0.134439	-0.647573	1.695995
29.	8.	0.	7.939866	-0.407318	-0.758164
30.	8.	0.	4.613307	2.904084	0.871883
31.	8.	0.	3.621008	0.479437	-1.438746
32.	6.	0.	-5.416979	-0.647382	2.181401
33.	1.	0.	6.330755	-1.416633	1.141069
34.	1.	0.	6.488725	-2.621044	-0.134890
35.	1.	0.	6.223043	-0.833845	-1.856389
36.	1.	0.	6.393154	1.144035	0.452687
37.	1.	0.	6.145703	1.525586	-1.239248
38.	1.	0.	4.132633	-2.641550	0.602850
39.	1.	0.	4.262662	-2.023260	-1.038577
40.	1.	0.	1.936818	3.046909	0.722881
41.	1.	0.	0.138199	0.932349	-1.375817
42.	1.	0.	0.704756	-2.391752	0.111010
43.	1.	0.	-0.207842	-2.046607	-1.337780
44.	1.	0.	2.330892	-2.009902	-1.593831
45.	1.	0.	1.472703	-0.653864	-2.274976
46.	1.	0.	-0.453316	2.231135	1.305711
47.	1.	0.	-0.693872	2.892991	-0.304388
48.	1.	0.	-2.598878	1.291988	1.175258
49.	1.	0.	-2.758483	1.715427	-0.513704
50.	1.	0.	-1.884332	-0.328157	-1.279428
51.	1.	0.	-2.811577	-1.125130	1.506021
52.	1.	0.	-3.392189	-3.324734	0.483154
53.	1.	0.	-2.638490	-2.838269	-1.037296
54.	1.	0.	-1.645779	-3.052001	0.411552
55.	1.	0.	-4.343764	-0.780247	-1.138840
56.	1.	0.	-7.435593	-0.122692	0.787854
57.	1.	0.	-5.904355	0.851426	-1.650018
58.	1.	0.	-6.223001	3.110531	-0.659125
59.	1.	0.	-7.043805	2.406066	0.738059
60.	1.	0.	-5.325043	2.075130	0.459784
61.	1.	0.	-8.259330	0.459815	-2.311637
62.	1.	0.	-8.836181	1.309179	-0.869218
63.	1.	0.	-7.978758	2.192370	-2.136995
64.	1.	0.	-8.219584	-1.446858	-1.117646
65.	1.	0.	-6.560748	-1.725817	-1.672264
66.	1.	0.	-7.118946	-2.440860	-0.158356
67.	1.	0.	3.040924	0.386287	2.322535
68.	1.	0.	2.996406	-1.385754	2.279439
69.	1.	0.	4.532152	-0.543145	2.455540
70.	1.	0.	-0.653333	0.122299	2.274445
71.	1.	0.	-0.533770	-1.617099	2.009204
72.	1.	0.	0.917278	-0.619542	1.985888
73.	1.	0.	8.345481	-1.229648	-1.062959
74.	1.	0.	-4.462160	-0.891807	2.650592
75.	1.	0.	-5.755627	0.315400	2.585499
76.	1.	0.	-6.151944	-1.393762	2.510738

7-3		Standard Orientation (Ångstroms)			
Center number	Atom number	Type	X	Y	Z
1.	6.	0.	5.970020	-1.654838	0.224726
2.	6.	0.	6.479602	-0.600649	-0.753541
3.	6.	0.	5.864470	0.766956	-0.440715
4.	6.	0.	4.364739	0.732537	-0.230501
5.	6.	0.	3.694511	-0.493448	0.484909
6.	6.	0.	4.445232	-1.793384	0.103653
7.	6.	0.	3.813879	2.059657	0.348237
8.	6.	0.	2.340609	2.133568	0.319978
9.	6.	0.	1.679641	1.039560	-0.104807
10.	6.	0.	2.541416	-0.144680	-0.557732
11.	6.	0.	0.207766	0.893244	-0.320246
12.	6.	0.	-0.358487	-0.472345	0.174463
13.	6.	0.	0.407424	-1.628896	-0.543105
14.	6.	0.	1.700328	-1.192071	-1.279813
15.	6.	0.	-0.777846	1.947739	0.190743
16.	6.	0.	-2.150149	1.217927	0.129271
17.	6.	0.	-1.865569	-0.282100	-0.206254
18.	6.	0.	-2.914111	-1.235236	0.422183
19.	6.	0.	-2.645772	-2.725176	0.121948
20.	6.	0.	-4.303987	-0.892756	-0.075321
21.	6.	0.	-5.406777	-0.587018	0.628499
22.	6.	0.	-6.765736	-0.359245	-0.051155
23.	6.	0.	-6.953737	1.046953	-0.706702
24.	6.	0.	-6.089884	1.297913	-1.951208
25.	6.	0.	-6.768703	2.186247	0.306119
26.	6.	0.	-7.140516	-1.496902	-1.018452
27.	6.	0.	3.483419	-0.437370	1.996588
28.	6.	0.	-0.203825	-0.610902	1.703272
29.	8.	0.	7.901388	-0.564510	-0.641039
30.	8.	0.	4.537649	2.940514	0.787497
31.	8.	0.	3.578709	0.425671	-1.430941
32.	6.	0.	-5.444793	-0.492510	2.137089
33.	1.	0.	6.271627	-1.370759	1.241086
34.	1.	0.	6.455318	-2.611944	0.006616
35.	1.	0.	6.188023	-0.904560	-1.771977
36.	1.	0.	6.323387	1.157010	0.474228
37.	1.	0.	6.089270	1.492368	-1.233772
38.	1.	0.	4.085348	-2.617261	0.730843
39.	1.	0.	4.225390	-2.064468	-0.932346
40.	1.	0.	1.859496	3.058775	0.621979
41.	1.	0.	0.091660	0.865750	-1.417523
42.	1.	0.	-0.239440	-2.115212	-1.279784
43.	1.	0.	0.660543	-2.403861	0.188986
44.	1.	0.	2.301720	-2.073717	-1.512173
45.	1.	0.	1.443440	-0.747285	-2.249316
46.	1.	0.	-0.532819	2.245934	1.215514
47.	1.	0.	-0.757152	2.857327	-0.416581
48.	1.	0.	-2.678247	1.300812	1.084689
49.	1.	0.	-2.809026	1.663743	-0.620367
50.	1.	0.	-1.927821	-0.406191	-1.298353
51.	1.	0.	-2.873968	-1.100585	1.508142
52.	1.	0.	-3.424089	-3.346932	0.574588

53.	1.	0.	-2.658711	-2.918252	-0.957279
54.	1.	0.	-1.680167	-3.060842	0.509787
55.	1.	0.	-4.400198	-0.969152	-1.159138
56.	1.	0.	-7.513143	-0.390050	0.754478
57.	1.	0.	-8.004237	1.069146	-1.033599
58.	1.	0.	-6.343211	2.266184	-2.396212
59.	1.	0.	-5.025430	1.319005	-1.698163
60.	1.	0.	-6.239493	0.535246	-2.721460
61.	1.	0.	-7.413501	2.055863	1.182341
62.	1.	0.	-5.731370	2.242467	0.654340
63.	1.	0.	-7.016195	3.152543	-0.146386
64.	1.	0.	-8.142332	-1.337040	-1.433196
65.	1.	0.	-6.445422	-1.580437	-1.858609
66.	1.	0.	-7.139670	-2.460321	-0.499334
67.	1.	0.	2.961087	0.462110	2.326839
68.	1.	0.	2.920989	-1.310570	2.343038
69.	1.	0.	4.452989	-0.459698	2.505765
70.	1.	0.	-0.735600	0.172969	2.250428
71.	1.	0.	-0.597508	-1.572895	2.045000
72.	1.	0.	0.844555	-0.563652	2.002759
73.	1.	0.	8.226688	0.122007	-1.238592
74.	1.	0.	-4.470331	-0.630053	2.608680
75.	1.	0.	-5.839479	0.478049	2.459252
76.	1.	0.	-6.123919	-1.251616	2.547880

**7-4**		Standard Orientation (Ångstroms)			
Center number	Atom number	Type	X	Y	Z
1.	6.	0.	6.014492	-1.834152	0.459832
2.	6.	0.	6.619179	-0.923514	-0.614286
3.	6.	0.	6.088451	0.506528	-0.473442
4.	6.	0.	4.586848	0.590237	-0.287953
5.	6.	0.	3.820003	-0.504255	0.537302
6.	6.	0.	4.485023	-1.883832	0.314544
7.	6.	0.	4.118718	2.002245	0.144300
8.	6.	0.	2.656540	2.176109	0.060078
9.	6.	0.	1.928606	1.090710	-0.266291
10.	6.	0.	2.713274	-0.192448	-0.565909
11.	6.	0.	0.455516	1.024314	-0.509121
12.	6.	0.	-0.218699	-0.235985	0.112950
13.	6.	0.	0.477247	-1.514498	-0.451552
14.	6.	0.	1.813861	-1.252712	-1.193250
15.	6.	0.	-0.463433	2.194980	-0.147459
16.	6.	0.	-1.882684	1.558580	-0.168721
17.	6.	0.	-1.697244	0.015347	-0.334807
18.	6.	0.	-2.832377	-0.787299	0.350709
19.	6.	0.	-2.672846	-2.316319	0.219212
20.	6.	0.	-4.166348	-0.383372	-0.240449
21.	6.	0.	-5.266067	0.092111	0.366827
22.	6.	0.	-6.502979	0.426576	-0.467437
23.	6.	0.	-7.753050	-0.412384	-0.068312
24.	6.	0.	-7.458677	-1.919658	-0.104543
25.	6.	0.	-8.966285	-0.098091	-0.958910

26.	6.	0.	-6.784609	1.944762	-0.438165
27.	6.	0.	3.581207	-0.272243	2.028040
28.	6.	0.	-0.109756	-0.214967	1.651389
29.	8.	0.	8.043782	-0.950867	-0.612357
30.	8.	0.	4.892775	2.864841	0.531668
31.	8.	0.	3.803173	0.209393	-1.467294
32.	6.	0.	-5.366820	0.348418	1.853388
33.	1.	0.	6.303571	-1.466760	1.455805
34.	1.	0.	6.437459	-2.839064	0.357926
35.	1.	0.	6.336299	-1.316200	-1.597734
36.	1.	0.	6.548448	0.983875	0.401979
37.	1.	0.	6.379784	1.112144	-1.338447
38.	1.	0.	4.055006	-2.613317	1.010760
39.	1.	0.	4.270844	-2.244251	-0.695014
40.	1.	0.	2.236190	3.159073	0.249331
41.	1.	0.	0.362428	0.884536	-1.599991
42.	1.	0.	0.656605	-2.220945	0.366768
43.	1.	0.	-0.186273	-2.032037	-1.151134
44.	1.	0.	2.354473	-2.194393	-1.312155
45.	1.	0.	1.609678	-0.899986	-2.211760
46.	1.	0.	-0.220784	2.589581	0.844871
47.	1.	0.	-0.363554	3.026288	-0.851398
48.	1.	0.	-2.421172	1.778212	0.758769
49.	1.	0.	-2.496972	1.962910	-0.977445
50.	1.	0.	-1.738988	-0.220261	-1.409666
51.	1.	0.	-2.816676	-0.539709	1.417853
52.	1.	0.	-3.511013	-2.825674	0.704386
53.	1.	0.	-2.667830	-2.623739	-0.833155
54.	1.	0.	-1.748771	-2.679025	0.677916
55.	1.	0.	-4.222661	-0.531248	-1.321694
56.	1.	0.	-6.266029	0.159274	-1.507244
57.	1.	0.	-8.024214	-0.142639	0.962955
58.	1.	0.	-8.339082	-2.495786	0.199512
59.	1.	0.	-7.188825	-2.238040	-1.119069
60.	1.	0.	-6.630188	-2.193516	0.554274
61.	1.	0.	-9.297606	0.939364	-0.863810
62.	1.	0.	-8.734828	-0.281771	-2.015593
63.	1.	0.	-9.814276	-0.738050	-0.692740
64.	1.	0.	-7.506837	2.232744	-1.206585
65.	1.	0.	-7.188271	2.264719	0.528928
66.	1.	0.	-5.864792	2.509009	-0.621041
67.	1.	0.	3.118382	0.691756	2.245746
68.	1.	0.	2.948516	-1.061427	2.447430
69.	1.	0.	4.534645	-0.307616	2.566465
70.	1.	0.	0.932621	-0.203492	1.975133
71.	1.	0.	-0.576586	-1.105365	2.083391
72.	1.	0.	-0.599126	0.657988	2.092779
73.	1.	0.	8.340544	-0.534959	0.209176
74.	1.	0.	-5.625290	1.393585	2.059575
75.	1.	0.	-6.156668	-0.262153	2.307352
76.	1.	0.	-4.438562	0.128300	2.382676

Table S3. Gibbs free energies[a] and equilibrium populations[b] of low-energy conformers of phomopyrone E (**9**)

Conformers of **9**	In MeOH	
	ΔG^{a}	P (%) ^{b}

**9-1**	-457765.88310258	96.02
**9-2**	-457763.99869005	3.98

[a]ΔG, B3LYP/ 6-31g (d, p), in kcal/mol. [b] Boltzmann-population.

Table S4. Cartesian coordinates for the low-energy reoptimized MMFF conformers of phomopyrone E (**9**) at B3LYP/6-31G(d,p) level of theory in gas

1.2

Conformer **9-1**		Standard Orientation (Ångstroms)			
Center Number	Atom	Type	X	Y	Z
1.	6.	0.	0.456833	1.825835	-1.604021
2.	6.	0.	-0.453333	1.782363	-0.573197
3.	6.	0.	-0.406471	0.733155	0.407044
4.	6.	0.	0.551992	-0.220947	0.303178
5.	8.	0.	1.452632	-0.203848	-0.699505
6.	6.	0.	1.466948	0.800657	-1.704885
7.	8.	0.	2.325667	0.701771	-2.553682
8.	6.	0.	0.473862	2.888343	-2.667282
9.	8.	0.	-1.388097	2.764543	-0.525189
10.	6.	0.	-2.367065	2.758021	0.509244
11.	6.	0.	0.732139	-1.386728	1.241687
12.	6.	0.	0.447274	-2.727394	0.525745
13.	6.	0.	-1.001557	-2.880539	0.057670
14.	8.	0.	-1.947249	-2.730525	1.115447
15.	6.	0.	2.136380	-1.376317	1.873507
16.	1.	0.	-1.118732	0.671235	1.216785
17.	1.	0.	1.445667	3.392811	-2.690216
18.	1.	0.	-0.307506	3.628895	-2.496903
19.	1.	0.	0.332608	2.444426	-3.658824
20.	1.	0.	-2.993718	3.631127	0.324547
21.	1.	0.	-2.987721	1.855577	0.473575
22.	1.	0.	-1.908840	2.848547	1.500801
23.	1.	0.	-0.018563	-1.272507	2.030715
24.	1.	0.	0.691518	-3.545179	1.218357
25.	1.	0.	1.118215	-2.836127	-0.334799
26.	1.	0.	-1.252355	-2.100124	-0.667363
27.	1.	0.	-1.125614	-3.847887	-0.451927
28.	1.	0.	-1.822189	-3.470279	1.724889
29.	1.	0.	2.909660	-1.478859	1.107345
30.	1.	0.	2.318738	-0.443486	2.415841
31.	1.	0.	2.239877	-2.205559	2.580416

Conformer **9-2**		Standard Orientation (Ångstroms)			
Center Number	Atom	Type	X	Y	Z
1.	6.	0.	0.254352	1.777982	-1.600705
2.	6.	0.	-0.232199	1.924490	-0.322229
3.	6.	0.	-0.123400	0.857971	0.634514
4.	6.	0.	0.471860	-0.302536	0.260708
5.	8.	0.	0.955386	-0.471730	-0.984894
6.	6.	0.	0.877630	0.535415	-1.986345
7.	8.	0.	1.341435	0.254117	-3.069033

8.	6.	0.	0.177211	2.843591	-2.658030
9.	8.	0.	-0.815403	3.109138	-0.010185
10.	6.	0.	-1.337026	3.319111	1.297819
11.	6.	0.	0.690592	-1.505078	1.145308
12.	6.	0.	0.058351	-2.781701	0.548154
13.	6.	0.	-1.459217	-2.707958	0.364034
14.	8.	0.	-2.007082	-3.937555	-0.092852
15.	6.	0.	2.196001	-1.709549	1.411536
16.	1.	0.	-0.500262	0.954315	1.642560
17.	1.	0.	1.176069	3.074131	-3.043095
18.	1.	0.	-0.272916	3.755696	-2.266485
19.	1.	0.	-0.411709	2.496339	-3.514090
20.	1.	0.	-1.745601	4.330065	1.291525
21.	1.	0.	-2.138986	2.609867	1.532739
22.	1.	0.	-0.553777	3.253800	2.061830
23.	1.	0.	0.195975	-1.285436	2.100365
24.	1.	0.	0.284558	-3.628554	1.205707
25.	1.	0.	0.533677	-2.989809	-0.418876
26.	1.	0.	-1.945311	-2.507998	1.325962
27.	1.	0.	-1.724860	-1.878377	-0.309171
28.	1.	0.	-1.662315	-4.097450	-0.981421
29.	1.	0.	2.729274	-1.902860	0.476637
30.	1.	0.	2.638290	-0.824671	1.879108
31.	1.	0.	2.350404	-2.562768	2.078907
